# Structural Annotation of *Mycobacterium tuberculosis* Proteome

**DOI:** 10.1371/journal.pone.0027044

**Published:** 2011-10-31

**Authors:** Praveen Anand, Sandhya Sankaran, Sumanta Mukherjee, Kalidas Yeturu, Roman Laskowski, Anshu Bhardwaj, Raghu Bhagavat, OSDD Consortium, Samir K. Brahmachari, Nagasuma Chandra

**Affiliations:** 1 Department of Biochemistry and Bioinformatics Centre, Indian Institute of Science, Bangalore, India; 2 European Bioinformatics Institute, Wellcome Trust Genome Campus, Cambridge, United Kingdom; 3 Institute of Genomics and Integrative Biology (CSIR), New Delhi, India; 4 Council of Industrial and Scientific Research, New Delhi, India; 5 The Open Source Drug Discovery (OSDD) Consortium, Council of Scientific and Industrial Research, New Delhi, India; Inserm U869, France

## Abstract

Of the ∼4000 ORFs identified through the genome sequence of *Mycobacterium tuberculosis* (TB) H37Rv, experimentally determined structures are available for 312. Since knowledge of protein structures is essential to obtain a high-resolution understanding of the underlying biology, we seek to obtain a structural annotation for the genome, using computational methods. Structural models were obtained and validated for ∼2877 ORFs, covering ∼70% of the genome. Functional annotation of each protein was based on fold-based functional assignments and a novel binding site based ligand association. New algorithms for binding site detection and genome scale binding site comparison at the structural level, recently reported from the laboratory, were utilized. Besides these, the annotation covers detection of various sequence and sub-structural motifs and quaternary structure predictions based on the corresponding templates. The study provides an opportunity to obtain a global perspective of the fold distribution in the genome. The annotation indicates that cellular metabolism can be achieved with only 219 folds. New insights about the folds that predominate in the genome, as well as the fold-combinations that make up multi-domain proteins are also obtained. 1728 binding pockets have been associated with ligands through binding site identification and sub-structure similarity analyses. The resource (http://proline.physics.iisc.ernet.in/Tbstructuralannotation), being one of the first to be based on structure-derived functional annotations at a genome scale, is expected to be useful for better understanding of TB and for application in drug discovery. The reported annotation pipeline is fairly generic and can be applied to other genomes as well.

## Introduction

Tuberculosis continues to be a major burden, causing about 4500 deaths per day [1]. The problem is worsened due to the deadly synergy of *Mycobacterium tuberculosis* (TB) with HIV and the emergence of multi-drug resistant varieties. Clearly, there is an urgent need for a better understanding of the bacillus. The annotation of the genome sequence of *Mtb*H37Rv identified 4047 genes, which comprises of 3988 protein coding genes [2], [3]. From a protein function standpoint, a practical way to convert vast quantities of raw sequence data into meaningful information is through transfer of annotation from known proteins to homologues in the target genome [4]. Indeed, advances in sequence-based bioinformatics approaches have become more reliable in transferring functional annotation, integrating sequence and protein family classifications. However, a finer appreciation of the molecular mechanisms within the cell is possible only with the structural information. Where available, protein structures provide much better functional insight than their sequences alone, for two reasons: (a) they provide a much higher resolution of information and (b) a much more sensitive approach for detecting similarities among proteins.

Currently, structures for 312 *TB* proteins have been determined through experimental methods [5], including those from community-wide structural genomics efforts [6], [7], amounting to a coverage of only 0.8% of the proteome. On the other hand, methods for structure prediction have improved tremendously in applicability, speed and confidence, which can be used to bridge the wide gap between sequence and structure [8], [9]. Here we describe an effort to annotate the structures of the *TB* proteome. Annotation has been achieved through a pipeline that integrates a number of different computational approaches. Obtaining the models at a genome scale provides one of the first opportunities to view the structural profile of the proteins in the organism and understand the cellular functioning in terms of structural scaffolds that facilitate the underlying molecular recognition events.

## Results and Discussion

### The structural annotation pipeline

To obtain a structural proteome of *M. tuberculosis* H37Rv, an integrated structural annotation pipeline was developed. Each model is annotated with metrics rating its quality, high and low confidence regions in the model and a range of features suggestive of its function. High-quality molecular models for 2511 proteins were derived from Modbase [10] and an additional 54 were derived through remote homology detection methods. Put together with 312 crystal structures from PDB, structural models for 2877 proteins are thus available. Functional annotation procedures as applied to the 2877 models involved detection of conserved residues in the protein family, location of ligand or DNA binding site(s), similarity with enzyme active sites, and finally possible ligand associations. In all, 1728 ligand associations have been found, providing functional clues.

**Figure 1:**
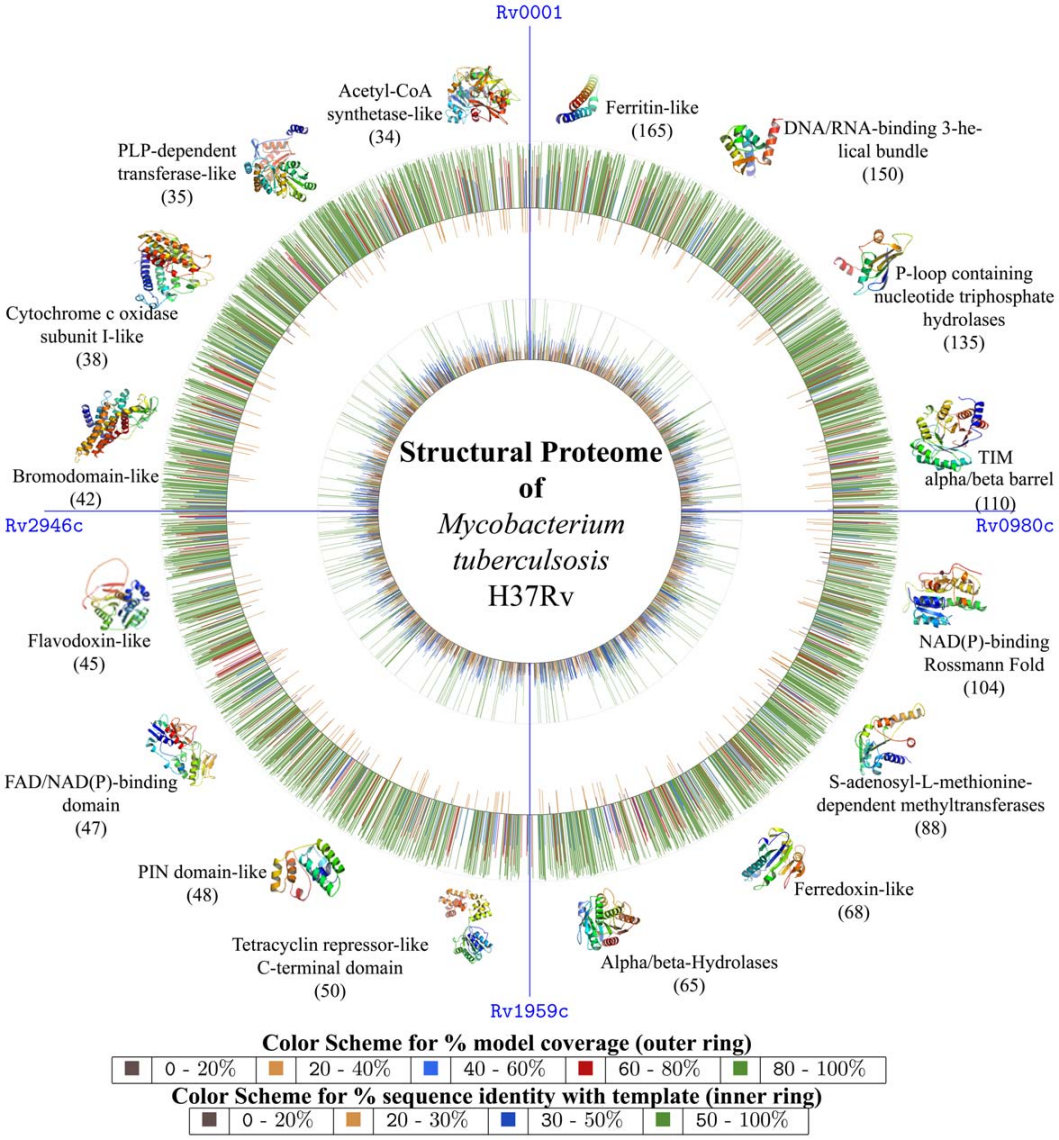
Schematic view of structural annotation. The figure corresponds to that of the circular map of the chromosome of *M. tuberculosis* H37Rv, similar to that reported earlier with its complete genome sequencing[2]. On both the outer and inner circles, radiating lines are drawn to indicate the parameters of the structural model for the corresponding protein in the genome view. The outer circle represents the model coverage in terms of the % of its polypeptide chain whereas the inner circle represents % sequence identity shared by each model with its corresponding template. The length of the lines in both cases are proportional to their values in %. The 100% mark is also shown for both the circles. In the outer circle, those models that had greater than 40% length coverage are drawn outside the circle where as those with a coverage of less than that are drawn inside the circle (for clarity). Length coverage is binned into 5 classes and color coded as indicated, while the levels of sequence identity are binned into 4 classes and color coded as indicated. The most commonly occurring folds in the modeled proteome are shown surrounding the outer circle and its frequency of occurrence in the modeled proteome is also mentioned.

Given the genome-wide nature of this study, two types of coverage are considered. First, the coverage of the genome or the number of different proteins modeled and second, the coverage of each protein or the number of residues in the individual polypeptides that could be modeled was analyzed. 2877 proteins, which is ∼70% of the proteome, have been modeled (Figure 1). 1427 of these showed complete length coverage, (>90%) (Figure S1), whereas 2233 models had length coverage of at least 50%. Typically coverage is lower for proteins in cell wall processes or insertion sequences categories. The proteins showing least coverage belonged to PE/ PPE and other membrane proteins, since in these, quite often only a domain could be modeled.

1500 unique structural templates from PDB were used for modeling the proteome, of which 1173 were from bacteria, 243 from eukaryotes including 44 from plants, 116 from archaea and 74 from viruses (Table S1).

### Confidence and Quality of the Structural Proteome

The procedures for modeling protein structures are now well established [11], [12], [13], [14], [15], most often resulting in models correct to an RMSD of <2 Å. As a validation exercise, crystal structures of proteins (312) that are solved have been compared with the corresponding models obtained through Modpipe workflow by excluding the crystal structures in the template search step. Folds of all the proteins were also obtained through 3D BLAST [16] and all the models indeed have the correct fold as that of the corresponding crystal structure, with an average RMSD of about 2 Å (Table S2), indicating high levels of confidence in the methodology used.

An important aspect of the annotation pipeline involved assessment of each model through different estimates of confidence. These include (a) statistical significance of alignments and extent of sequence similarity (b) geometry and stereochemistry (c) consistency in residue contacts and solvent accessibility profiles with high resolution crystal structures [17]. A large number of proteins (30%) could be modeled based on obvious homology to known structures, while another 33% could be modeled based on reasonable sequence identity of 20–30%, yet with statistically significant E-values (<0.0001 from PSI-Blast). Less than 30% of the models were derived from templates without significant sequence similarities, but based on high compatibility with the structural folds [18], [19], [20].

Criteria that were considered for evaluation included DOPE scores output by Modeller [11] and E-values obtained from BLAST [21] and PSI-BLAST [22], whereas the geometry of structure was assessed using Procheck [23]. The, geometric parameters of each model were within acceptable ranges for bond lengths, bond angles, planarity and dihedral angles and the ϕ, ψ dihedral angles of the peptide bonds were predominantly in highly favored regions of the Ramachandran plot. DOPE scores are found to be reliable since they are based on residue environments of the query sequence and structural compactness [18]. The correctness of the models in the last category was assessed independently through a fold prediction exercise using Genthreader [24]. Computation of inter-residue contacts, consistency with secondary structure predictions, solvent accessible surface area distribution and radius of gyration of the model was carried out using ProQ [17], [25] which uses a neural-network based method to test the quality of the protein. The quality of the protein models was also evaluated by ERRAT that works by analyzing the statistics of non-bonded interactions between different atom-types. Details of various criteria used are provided in Table S3.

### Fold Distribution in the Proteome

**Figure 2:**
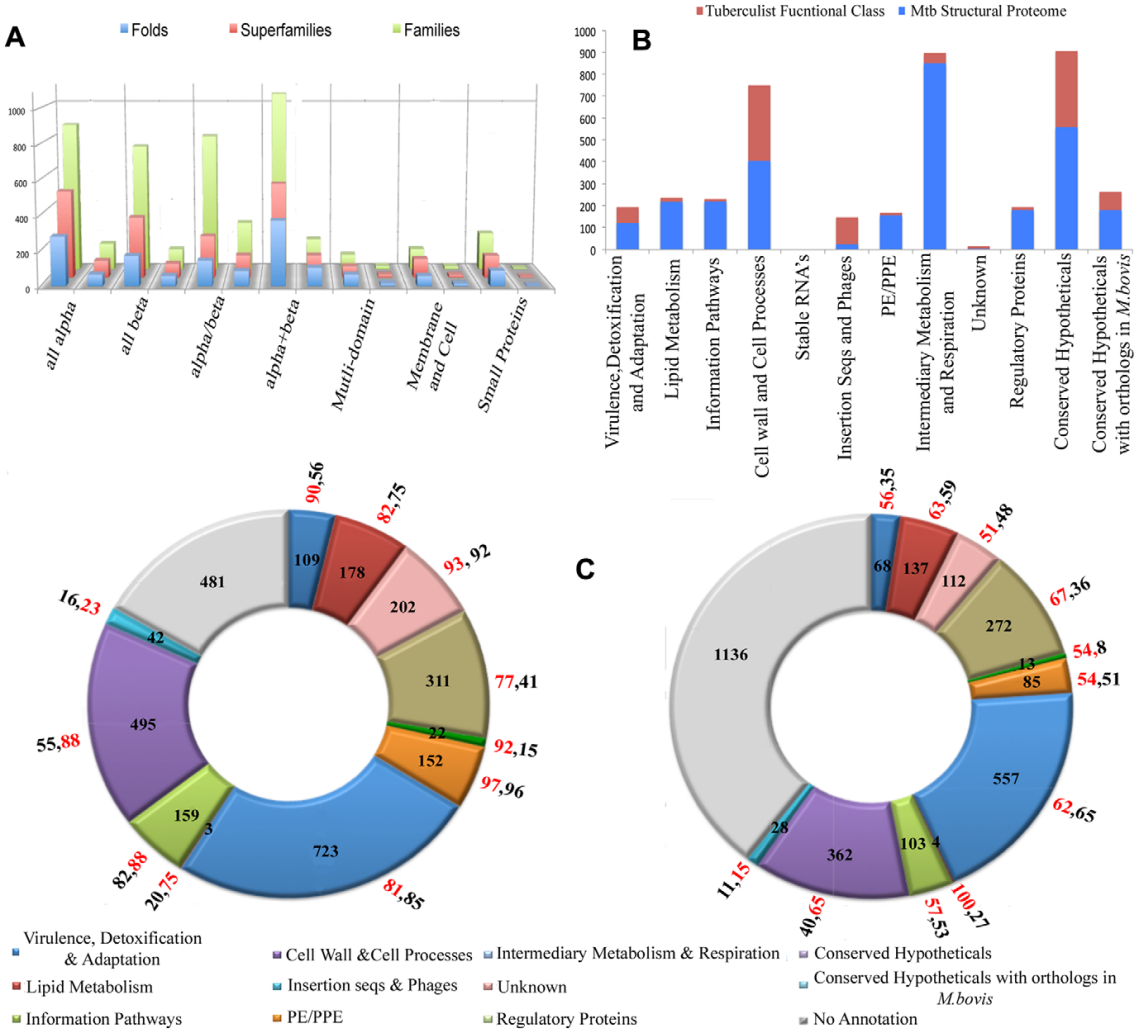
Structural Proteome Coverage. Coverage of models across (A) SCOP classes and (B) Tuberculist functional classes associated with the TB genome sequence (C) Function Characterization through fold association and ligand association. In (A), a histogram showing the distribution of folds, their super-families and families across the major SCOP classes are shown in the left for each class. A corresponding bar is shown on the immediate right to indicate the number of such occurrences in the modeled part of the TB proteome. In (B) the number of proteins in different categories of Tuberculist are shown as red bars and the numbers of proteins modeled in each category are highlighted as blue bars respectively. In (C) number of proteins that have been characterized through fold association and ligand association through different categories of tuberculist are shown. The values in % of proteins in modeled proteome have been highlighted in red, and % in total genome is highlighted in black and shown radially across each category.

The availability of the structural models spanning the complete genome of TB gives us the opportunity to analyze the fold content and fold preferences of this organism. From another perspective, it can help identify the set of folds that are sufficient for sustaining life in the bacterium. Conversely, useful insights can be drawn from folds that are not represented in the genome. Assessments involving the SCOP database [26] show that all the seven major structural classes are observed in the modeled proteome (Figure 2A). Of the 1195 known folds, 419 (∼30% of all known folds) are seen in the proteome, with a preference for domains from α/β class (36.5%). Similarly, 20% and 24% of all folds at the family and super-family levels are observed. It must be noted, that the methodology used for obtaining the structural models could not have captured novel folds in TB. However, we anticipate that this may not be a high number since the coverage of the genome is significant. For the proteins whose structures could not be determined through above methods, a threading exercise assigned an existing fold with high confidence for 143 of 1211 proteins [27] (Table S4). This suggests that more proteins not included in the current dataset, may in fact exhibit one of the known folds.

The top folds (Figure 1) (full list in Figure S2 & Table S5) in the modeled TB proteome include ferritin-like, DNA/RNA-binding 3-helical bundle, P-loop containing nucleoside triphosphate hydrolases, TIM beta/alpha barrel, NAD(P)-binding Rossmann-fold, SAM-dependent methyl transferases, ferredoxin-like and alpha/beta-hydrolase folds. Together, these constitute 887 proteins (∼30% of all modeled structures in the proteome). A power-law distribution of the folds has been reported in many genomes [28], consistent with the trend seen here in terms of some folds occurring many more times than most other folds. Interestingly, a majority of the most common folds in the TB proteome are involved in metabolism, consistent with the trends of distribution of protein fold categories in prokaryotes [29].

Although folds associated with metabolism rank highly in TB, the topmost occurrence is a ferritin-like fold adopted by the N-terminus of the PE and PPE proteins. This is not surprising, since nearly 180 proteins of these families are known to exist in TB. The high representation of this fold, which is known to be associated with iron storage, can perhaps explain the adaptation mechanism of the microorganism to overcome oxidative stress [30] and protect it from iron–dependent killing by hydrogen peroxide in macrophages.

Analysis of the distribution of models across the 12 Tuberculist [31] functional categories indicates that all classes are covered, although to varying extents (Figure 2B).The least modeled category corresponds to the class of insertion sequences and phages (16%), while the most covered categories are those involved in metabolism (94.7%) and information pathways (95.2%). About 262 fold types are associated with enzymes and hence with metabolism, 47 fold types with regulation and 87 fold types with information pathways. Since the coverage of proteins involved in metabolism is almost complete, it is significant to note that merely 206 different fold types are sufficient to propel the organism's metabolism while others are involved in different types of regulation. 741 proteins were associated with DNA-binding folds. Of the remaining 1121 ORFs that could not be modeled at this time, 600 are annotated as conserved hypotheticals, while another 314 are associated with cell wall processes. A sequence analysis based prediction of trans-membrane helices [32], indicated that 850 proteins contained at-least one membrane-associated segment. Of these, structural models are available for 251, many of which participate in cell-wall processes. Further, of the membrane associated proteins, the cytochrome C oxidase subunitI-like fold occurs most often (38 occurrences), followed by glycerol-3-phosphate transporter and SNF like folds, (15 occurrences each). Among the least commonly occurring folds, 9 folds are associated with extracellular processes such as toxin-anti toxin system (YefM-like) and that are implicated in cell adhesion.

### Inferring protein function from structures

Functional annotation has been carried out by broadly three different approaches: first through known fold-function associations, second through identification of known sub-structural motifs and third through binding site identification, comparison with sites of known ligands and a subsequent ligand association. Fold to function assignments have been possible for 2832 different proteins, using the classification scheme reported earlier [33], [34]. Seven broad functional categories associated to 50 different functions have been linked to the models of TB. Validation for this part of the annotation pipeline has been carried out on the 312 protein test set. Binding site prediction, comparison and ligand association computed using PocketDepth and PocketMatch were compared with that in their corresponding crystal structures (Table S6), which indicated that ligand associations and hence annotations were in most cases correct, when the detected similarity was atleast moderately high (above the threshold of 0.4 P_max_ PocketMatch Score). Similarly a comparison between the fold-based function annotation and the GO terms, also indicated broad agreement (Table S7).

While the annotations and a broad functional category were available in the databases for many proteins based on literature and sequence analyses, several new associations have been possible through modeling and structural analyses, some examples are described later. Of the 639 conserved proteins annotated as hypothetical proteins in the TB genome, 560 proteins can now be associated with fold-based function annotation through the pipeline reported here. While 282 of these 639 proteins have an association with one or more functional categories of COG, our protocol has provided an annotation for 560 proteins through structural analysis.

A list of weakly annotated proteins with terms such as “PROBABLE” or “POSSIBLE” and largely derived through sequence comparisons are available in Tuberculist [31]. Such proteins have not been experimentally characterized. This includes around 1134 with “PROBABLE” functional term associated and 386 proteins with “POSSIBLE” functional term associated with its function in the database. Structural studies performed on such proteins have increased the confidence of annotation. The mode and level of annotation is highlighted in the database.

Modeled proteins when queried against Pfam database show the presence of 1165 different Pfam domains. Scanning for various structural motifs using ProFunc [35], showed the presence of 441 known ligand binding motifs, 741 DNA binding motifs, and 647 patterns of enzyme active sites. Surface clefts and binding pockets were computed in all the models, using multiple methods. The predicted sites when compared with a representative set of binding pockets from PDB structures in a massive computational exercise involving 3,92,75,635 comparisons, resulted in 771 ligand associations for 1728 proteins. Associating a ligand (Figure 2C) through prediction of recognition capability at the structural level is extremely useful in confirming putative functional associations for proteins and can also provide new functional clues. Highly conserved residues in each protein family, have been identified through sequence analysis, and made available in the database as heat maps.

**Figure 3:**
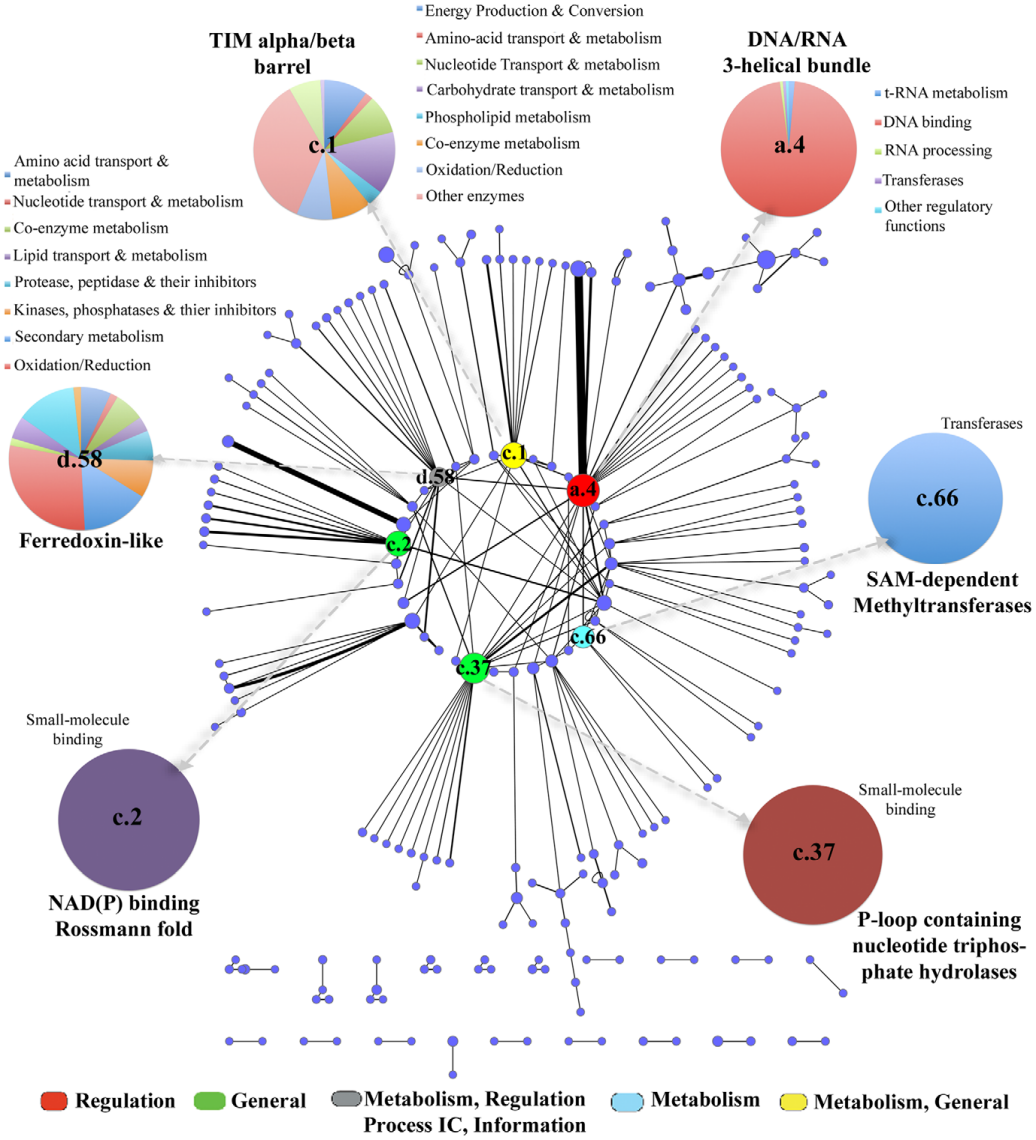
Fold combinations observed in modeled proteome. A weighted undirected network representing fold associations in the modeled part of the TB proteome. Each node (circle) represents a fold which is weighted based on the frequency of occurrence in the modeled proteome whereas an edge (line) represents the co-occurrence of those fold types in various proteins. The thicker the edge, the more number of times the pair of domains co-occur. The coloring scheme is based on the function assignment for topmost occurring folds (as indicated in the color key). Distribution of super-families in each of the 5 topmost occurring folds are also shown as labeled pie-charts.

489 modeled proteins were observed to be in the ‘multi-domain’ category, since they contained more than one fold in their polypeptide chains, as judged from their corresponding templates. An analysis of SCOP domain associations indicated that certain domain combinations were often re-used. A network constructed to visualize the fold associations (Figure 3), has 207 nodes (fold types) and 228 edges (fold combinations). P-loop containing nucleoside-triphosphate hydrolases was the most highly associated fold in the network, while the C-terminal domain of tetracylin repressor-like fold associated with DNA/RNA-binding 3-helical bundle fold, was the most highly recurring fold pair (full list in Table S8). Tetracycline repressor proteins are known to play an important role in ribosomal protection and help in regulation of various efflux proteins [36], [37].

### Broad classification of functional annotation into four categories

**Figure 4:**
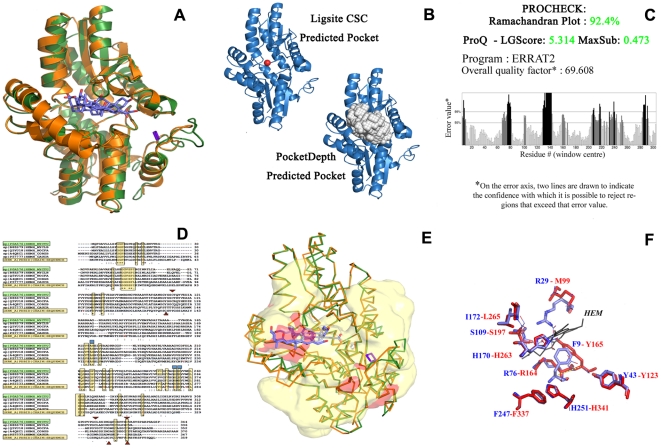
Aspects of Annotation. Example of Rv1485 showing different kinds of analysis carried out to derive the annotations. (A) Superposition of Rv1485 with its template 1HRK_A. (B) Binding Site prediction using LigsiteCSC and PocketDepth. (C) Errat output of the Rv1485 showing overall quality factor of ∼70% by evaluating the non-bonded interactions between different atom types. (D) Multiple sequence alignment with selected sequence neighbors, highlighting conserved catalytic site residues (in triangles) (E) Predicted ligand binding pockets in red surface, the expected ligand binding site as determined by superposing the template shown as sticks and (F) Association of the heme ligand to the predicted binding site (residues in red) based on high similarity to a known heme binding site by searching against PDB pockets (blue).

An example annotation of well characterized protein Rv1485 is illustrated in Figure 4. Rv1485 (P0A576: 344 residues), is annotated as a Ferrochelatase that is involved in Porphyrin metabolism. This enzyme (4.99.1.1), catalyzes Ferrous insertion into Protophyrin IX to form Proto-heme [38]. While well characterized in animals, bacterial ferrochelatases were discovered much later and seen to differ from animal homologues. Eukaryotic ferrochelatases, typically possess three regions, an N-terminal organelle targeting region that is proteolytically cleaved, a second core region of 330 residues sharing homology with bacterial ferrochelatases and a C-terminal region that contains the dimerization motif as also three of the four cysteine ligands of the 2Fe-2S cluster [39]. It is suggested that mycobacterial ferrochelatases differ from their eukaryotic counterparts in that they are monomers that are not membrane-associated. Rv1485, was hence selected as an ideal test case for whom annotation through our structural pipeline was determined and compared with existing information. Firstly, 1HRK (A chain, human ferrochelatase, 359 residues) was selected as a template to model Rv1485 (Figure S3). The generated model could be superposed on the template with less than 0.9 Angstrom RMSD (Figure 4A). Further, other quality checks were performed to asses the quality of the model using ProCheck, ProQ and ERRAT (Figure 4C). Multiple sequence alignments of Rv1485 and homologues from other mycobacteria, Caulobacter crescentus (a bacterial ferrochelatase that is dimeric), S. pombe and human ferrochelatases showed a high conservation of residues in the protein core (Figure 4D). The alignments show that like the eukaryotic ferrochelatases, such heme synthases also possess a C-terminal region with some of the Cysteine ligands of the 2Fe-2S clusters. The alignments show that S. pombe ferrochelatase contains cysteines analogous to the [2Fe–2S] four cluster-ligating cysteines that are found in animal ferrochelatases. However, C. crescentus ferrochelatase does not possess cysteines in these same position and mycobacterial ferrochelatases possess four-cysteine ligation residues involving C158, C332, C339, and C341 (Figure 4D). Examination of the substrate-free and bound forms of the template enzyme show an open active site pocket that is closed through conformation change in the substrate-bound enzyme. Indeed, studies have shown that the active site pocket is closed around the porphyrin macrocycle with a number of active site residues that have reoriented side chains. An important role for a hydrogen bond network involving H263, H341, and E343 has been suggested in the reorganization of active site side chains. Interestingly, a similar network of residues is also seen in the mycobacterial ferrochelatase. PocketDepth [40] and LigsiteCSC [41] predictions, made on the modeled protein identified two pockets that overlap with the template pockets harboring the 2Fe–2S cluster and the co-crystallized ligand (cholic acid, 1HRK_A). These sites are also detected by the cleft predictions of the ProFunc [35] server (Figure 4E). An overlap of the binding sites show an independent comparison of the pocket overlap with a known binding sites (Figure 4F). These comparisons further show an extensive overlap with the binding pocket of other ferrochelatases.

**Figure 5:**
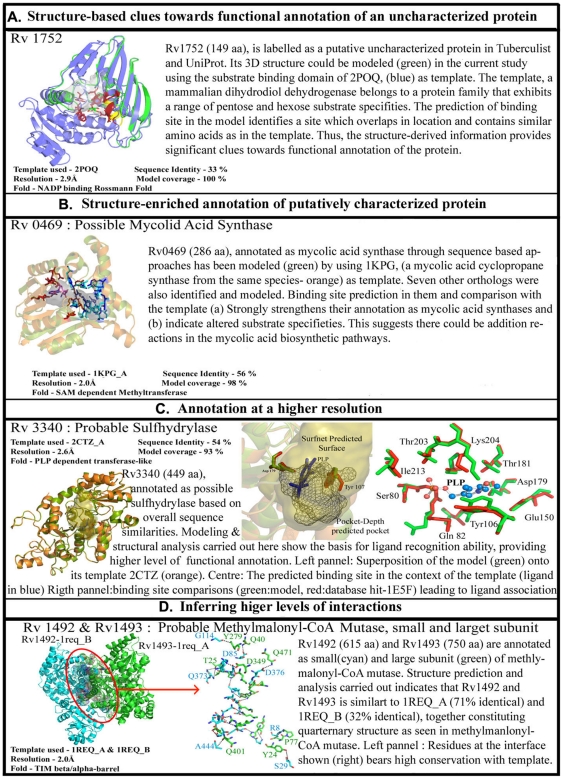
Different facets of annotation. Different levels of annotations are shown in the table, from assigning the putative function to uncharacterized protein (Rv1752), to increasing the confidence of putative and uncharacterized proteins (Rv0469,Rv2503c), assigning the binding sites of ligand to most proteins(Rv3340) and predicting the quaternary structure of proteins as well(Rv1492-Rv1493).

Such annotations lead to insights about protein functions at different levels (Figure 5). Some enrich available information of the protein while others provide finer detail such as identifying substrate specificities. More importantly, new annotations of function for completely uncharacterized proteins are obtained in some cases. In some, functional regions in the proteins are identified, while in some others higher order interactions and assemblies can be inferred. Some examples to illustrate these are listed in Figure 5. Most of the proteins from a genome sequence get their initial identification through an automated process by transferring annotation from a sequence homologue with known function. There can be several instances where sequences similarities on the whole are significantly high, but where the function has diverged considerably. A typical example would be α-lactalbumin, which is similar to lysozyme, in sequence and overall fold, but differs from the latter in function, due to mutation of key residues at the binding site [42]. A structure-based confirmation of the annotation, especially by comparing the binding sites and ligand binding ability, is therefore more meaningful.

Thus the broad categories of annotation are:

- Augmenting confidence of sequence-based annotation- confirming existing annotation: Rv0469 (Figure S4), a possible mycolic acid **synthase**, was modelled in Modbase with 1KPG_A as template. 1KPG (P0C5C2) is a cyclopropane-fatty-acyl-phospholipid synthase1. Mycolic acids are major components of the cell wall of *Mycobacterium tuberculosis*. Several studies indicate that functional groups in the acyl chain of mycolic acids are important for pathogenesis and persistence. There are at least three mycolic acid cyclopropane synthases (PcaA, CmaA1, and CmaA2) that are responsible for these site-specific modifications of mycolic acids [43]. Each enzyme acts at specific positions in the proximal or distal end of the mycolic acid fatty acyl chain. All three enzymes are structurally similar and have a seven-stranded α/β fold, similar to other methyltransferases, with the location and interactions with the cofactor S-adenosyl-l-methionine conserved. The structures of ternary complexes demonstrate the position of the mycolic acid substrate-binding site. Comparison of the structures also suggests that these enzymes catalyze methyl transfer via a carbocation mechanism in which the bicarbonate ion acts as a general base. 1kpgA also adopts a seven-stranded alpha/beta fold similar to other methyltransferases with the location and interactions of cofactor S-adenosyl-l-methionine conserved. The crystal structure of 1KPG_A is available with cetyl trimethylammonium bromide (CTAB) bound in the acyl-lipid binding pocket of the enzyme [43]. The active site pocket of 1kpgA is known to be lined with hydrophobic residues and interactions between the lipid and the protein are described as entirely hydrophobic in nature. Therefore, the ligand and cofactor-binding pockets in 1kpgA were compared with the predicted binding pockets of modeled Rv0469. Further, earlier experiments have suggested that the carbocation intermediate of the reaction mechanism may be stabilized by cation-π interactions during catalysis, specifically by the aromatic ring of the Tyr-33 in 1kpgA. Multiple sequence alignments of Rv0469 with other known mycolic acid cyclopropane synthases such as *cmaA2* (P0A5P0), *pcaA* (Q7D9R5), *mmA2* (Q79FX6), *mmA3* (P72027), *mmA4* (Q79FX8) and *cmaA1* (1KPG_A) P0C5C2, show a high conservation of residues involved in binding S-adenosyl methioinine (cofactor), as also residues involved in ligand interactions. Ligand binding-site predictions using PocketDepth [40] and cleft predictions in the ProFunc [35] server when applied to the modeled Rv0469, identify potential substrate binding and co-factor binding sites with high confidence. Indeed 86% of the cofactor-binding residues in 1kpgA are seen at topologically equivalent positions in Rv0469. E124 that shows hydrogen bonds with N6 of SAH is replaced with D123 in Rv0469. Assessments of the substrate-binding pocket show 85% of the ligand-binding residues in 1kpgA are also conserved in Rv0469. Further, cation-π interactions mediated by Y33 to stabilize the carbocation intermediate are conserved (Y32) in Rv0469 as well. Since the ligand-binding pocket is 9 Å from the surface, it has been suggested that longer chain length mycolic acids can also be accommodated into the pocket. Indeed, more detailed assessments involving the actual substrate (mycolic acids of varying chain length) within the predicted ligand binding pockets of Rv0469 are required to shed further light on the mode of action of Rv0469 and nature of interactions.
- Providing higher resolution information to support annotation-details in terms of binding site residues, presence or absence of motifs: More detailed annotation with better resolution can be obtained in certain cases. For example, the model of Rv3340 (O55390) was obtained by considering 2ctz_A as template having 54% sequence identity. 2CTZ_A is a probable o-acetyl homoserine sulfhydralase, which has PLP bound to it. The active site information was obtained from CSA [44] shows that Tyr107 and Asp180 are important residues involved in catalysis and the corresponding residues in the model are also conserved and occupied the same 3D co-ordinates. The co-factor used by this enzyme as also the template is PLP. The residues that constituted the PLP binding site were conserved, these residues (within 4 Å radius of PLP) were extracted and compared to all known binding sites of ligands (residues within 4 Å of the ligand atom) in PDB using PocketMatch [45], in this case the topmost scoring site was PLP binding site of 1e5f which is a methionine gamma-lyase from *Trichomonas vaginalis.* The alignment of the binding site residues can be visualized in Figure 5C and MSA in Figure S5.
- New annotation: There are still a number of proteins in the TB database, for which no functional information is available. The strategy used here for the functional annotation can be applied to such proteins to gain new insights. Rv1752 (O06789), being one such putative uncharacterized protein that was modeled, based on PSI-BLAST searches against the PDB database, with a mammalian dimeric dihydrodiol dehydrogenase (DD) as template (2poq: Macaca fascicularis: 7e −30, 33% identity, 53% positives). The structure of DD, an oxidoreductase, shows two domains, an N-terminal co-enzyme (NADPH)-binding domain and a C-terminal substrate-binding domain. Earlier investigations have shown that DD, identical to NADP+-dependent D-xylose dehydrogenase, is a close homologue of prokaryotic NADP(H)-dependent glucose-fructose oxidoreductase (GFO) and 1,5-anhydro-D-fructose reductase (AFR), although these enzymes differ in their coenzyme-binding affinities [46]. Further, it has been suggested that the C-terminal domain of DD shows striking resemblance to bacterial oxidoreductases and conserves a specific sequence motif GGX3DX3Y in its catalytic domain. DD is known to oxidize a broad range of substrates (pentoses, hexoses, 3-deoxyglucasone *etc.,*) We find that alignments of Rv1752 and DD involve only the C-terminal substrate binding domain of DD and exclude its N-terminal co-enzyme binding (NADPH-binding) domain. Interestingly, gene-neighborhood studies and searches in PFAM database show that Rv1751 (O65936) is a putative monoxygenase that potentially binds FAD. Figure 5A shows a superposition of the C-terminal domains of DD and modeled Rv1752. The superposition shows a conservation of the catalytic residue Y180 in topologically equivalent positions in the two proteins. F99, D2, Y6 and F43 of Rv1752 are also seen to be equivalent to the substrate-binding residues F279, D1176, Y180 and Y217 of DD. Other residues that line the inhibitor-binding pocket of DD and implicated in determining its broad substrate specificity include Trp125, Phe154, Trp254 and Phe279 [46]. We have compared the predicted clefts from ProFunc [35], the top–five predicted pockets from PocketDepth[40] and inhibitor-bound template crystal structure and show that these regions overlap appreciably. Our findings suggest that Rv1752 is perhaps an oxidoreductase whose co-enzyme binding requirements are met by its gene-neighbor Rv1751, a potential FAD-binding monoxygenase. Further, multiple sequence alignments of Rv1752 with homologues identified in searches in Uniprot and PDB show high similarities with other proteins from Mycobacteria (Figure S4). Also, the sequence motif conserved in DD and related homologue (GFO and AFR) is seen to be partially conserved in Rv1752.
- Functional assemblies: In some cases, a protein-protein assembly mimicking the quarternary state of the protein in another species, can also be obtained from the structural proteome. Rv1493 (P65487) has been functionally annotated as a probable methylmalonyl-CoA mutase large subunit. Methymalonyl-CoA mutase catalyses the isomerization of the succinyl-CoA to methylmalonyl-CoA during synthesis of propionate from tricarboxylic acid-cycle intermediates using adenosylcobalamin as co-factor. Through our annotation pipeline we can improve the confidence in the function assignment and also gain insights in the active site of the enzyme along with ligand and co-factor binding sites. This protein has no derived crystal structure; hence the model generated would be of value as methylonyl-CoA enzymes are known to be important due to their interaction with the metabolite succinyl-CoA and methymalonyl-CoA that are utilized in many essential pathways. The modeled structure available would help us in computer aided drug design and might prove to be a useful target as no human homologue was detected in BLAST searches against UniProt [47] database.

The modeled structure having a sequence coverage of 98.27% was generated by using the A chain of Methylmalonyl CoA Mutase[48] from *Propionibacterium freudenreichii* (1REQ) as template (71% sequence identity). The template used also has adenosyl cobalamin (Vitamin B12) along with Desulfocoenzyme A (DCA) bound to it. This would help us map the binding site residues on our protein of interest. The active site residues of the template was obtained from CSA (Catalytic Site Atlas) [49] that includes Tyr89, His244, Lys604, Asp608, His610 which aligns well with the modeled protein residues Tyr98, His253, Lys616, Asp620 and His622 respectively using MUSTANG[50] (Figure S6). After superposing the model generated with the template, it was seen that cobalamin binding site was clearly predicted from PocketDepth [40] Algorithm and perfectly overlaps with SURFNET [51] predicted highly conserved cleft. The active site residues are also possess the same projection as their template.

1REQ, the template used here was found to be a heterodimeric protein [48] with alpha and beta chain. Rv1493 here represents the alpha chain. For it to be functional and stable it would need its corresponding beta chain. Since in prokaryotes, the genes which are tightly linked functionally end up being in the same operon, a gene neighborhood analysis was performed and Rv1492 (P65845) was seen to be present in the upstream. Interestingly, the model of Rv1492 (with no known crystal structure till date) was generated using B chain of 1req protein itself. And hence even quaternary structure of essential proteins can be determined through such studies to get interface interacting residues which would be of very high importance as sometimes protein-protein interface can also be targeted [52] to disrupt the functioning of the enzyme. Although no experimental studies have been done to confirm the binding of cobalamin to this protein or show the presence of heterodimer, such structural studies improves our confidence in prediction. Further the assembly structure of Rv1492 and Rv1493 was built and evaluated using *Protein Interaction Calculator* [53] to obtain the list of interacting residues at the interface as seen in Figure 5D.

### Conclusion

In summary, the structural annotation reported here covers a significant portion of the TB proteome. The resource, providing functional clues for about 2877 proteins is likely to be very useful to tuberculosis, genomics and structural biology researchers. The annotation has provided insights about the folds that predominate, common fold combinations and more interestingly indicate that cellular metabolism can be achieved with only 219 folds.

The large-scale annotation pipeline used here to derive biological insights about *M. tuberculosis* is fairly generic and can be readily applied for other organisms as well. High confidence molecular models can now be obtained for several proteins, which together with the experimental structures available for that species can provide a first glimpse of the structural proteome of that organism. The MODBASE database already has genome scale protein structures for several organisms including that of human. The structural model of the protein can then be used for identifying possible function at different levels. Starting with fold-based function annotation, analysis can be followed up by sub-structure searches, binding-site detection through PocketDepth and ligand associations through PocketMatch and ProFunc, which can be applied for all the proteins to gain a deeper level of understanding about functioning of the organism. Besides providing new functional associations through modeling and structural analyses, our pipeline augments confidence of sequence-based annotations. More importantly, it provides annotation at a higher resolution by identifying key residues and motifs in the functional sites of protein molecules. Thus genome-scale structural modeling and analyses can be widely used for higher-resolution genome scale annotation.

## Materials and Methods

### Pipeline for Structure Annotation

**Figure 6:**
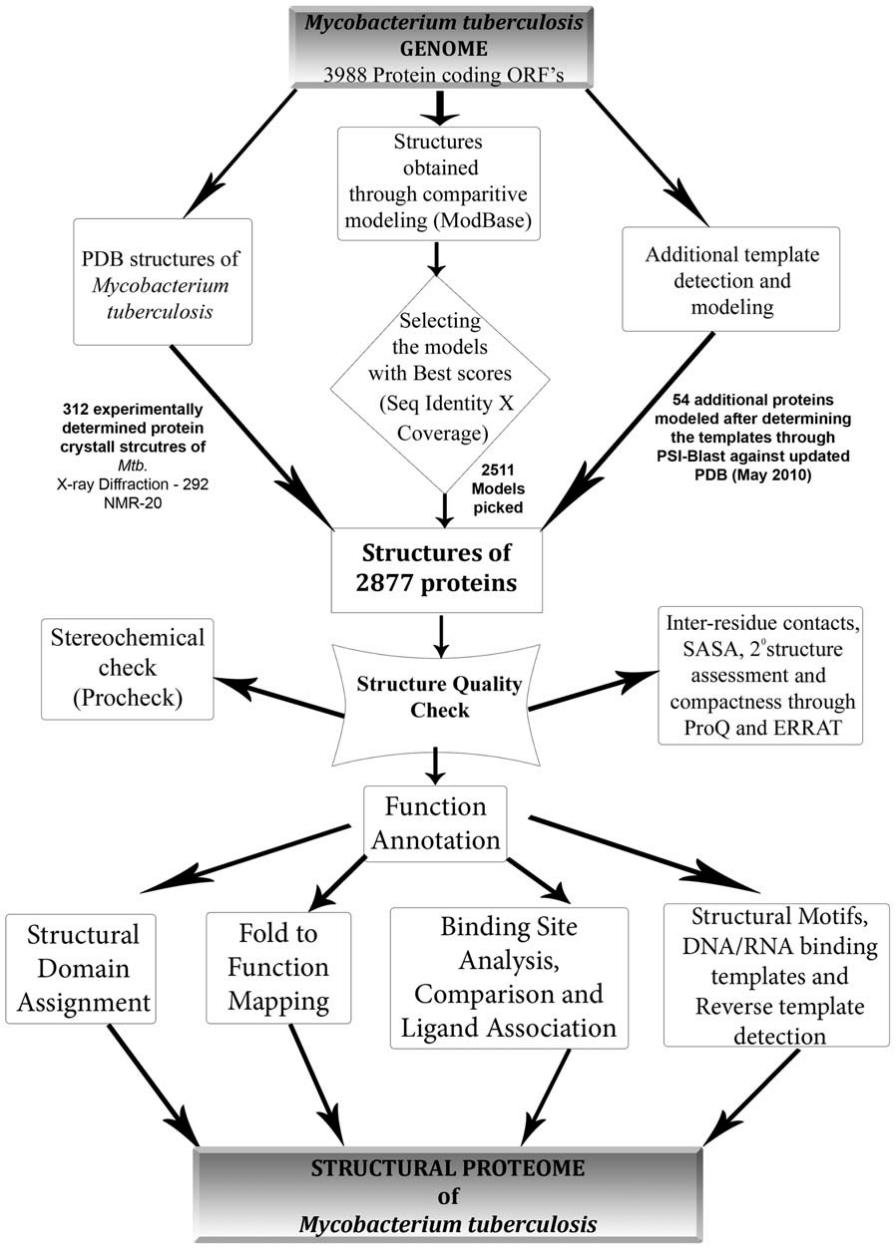
Structural annotation pipeline. Pipeline for annotation that was used for the individual proteins in the dataset, showing different steps of quality check followed by various techniques used to annotate the protein.

The pipeline is illustrated in Figure 6. Broadly, it starts with obtaining structural models of the individual proteins, followed by subsequent steps of structure validation. The models are then subjected to different types of analyses as a means to obtain functional annotation. The individual steps are described in detail.

### Obtaining Models

Of the 3998 predicted protein coding genes in *Mycobacterium tuberculosis,* experimentally determined structures [54] are available for 312 proteins. Structural models were hence derived for the remaining proteins using Modpipe (Figure S7), a widely used software suite and its associated database ModBase. The latest version of MODBASE (ftp://salilab.org/databases/modbase/projects/tdi/models/) included 5913 models (2808 *TB* proteins) for TB [10]. Since multiple models were available, they were ranked considering sequence identity and query length coverage (ranking score (Z) = product of % sequence identity and % length of query sequence in the alignment), DOPE score [18], [19] (Discrete Optimized Protein Energy) and MPQS (ModPipe Quality Score). For proteins where no models were available in Modbase, templates were identified for through remote homology detection techniques using PSI-BLAST [22] searches against the PDB, with E and H-values of 0.0001 and up to five iterations. The hits identified in the searches were further filtered for alignment coverage of at least 50% of the query length or a minimum domain length of seventy residues. In each case, the topmost hit was selected as a template for each query and modeled using modeler 9v7. Each such model was subjected to quality checks, as described in the earlier section. Modpipe was also used to assign the structures through sequence-profile search for these proteins wherein the templates obtained matched with the hits obtained from protocol mentioned above.

### Quality estimation of Protein Structures

The proteins for which crystal structures became available eventually (312 proteins) were selected and modeled separately through Modpipe 2.2 (care was taken to rule out the possibility of solved crystal structure being used as template itself). The models obtained were compared to actual crystal structures using DALI. The fold of the crystal structures and the model structures was evaluated using 3D-BLAST which searches for longest common substructure called SAHSPs (structural alphabet high-scoring segment pair). In 290 cases the fold obtained matched with the corresponding crystal structure (Table S2).

Each model was subjected to several quality checks. First, gross errors in the modeling process were ruled out, superimposing each model onto its corresponding template using CE [55]. The main criterion used to judge the quality of the protein model is the normalized Z-dope score, where a negative score always represents a better model. Almost all the models included have negative normalized Z-DOPE [18], [19] score with an average value of -0.89. Z-dope is based on score obtained from statistical potential. There are various other criteria to judge the quality of the protein model, for example ProQ [17] is a neural network based predictor depending upon number of structural features to predict the quality of the protein model. ProQ reports two scores – LGscore and MaxSub score, both having an average of 3.16 and 0.29, which can be considered to be good. ERRAT [56] is another such program that works by analyzing the statistics of non-bonded interactions between different atom types, an average quality factor of 64.931%. Moreover PROCHECK [23] algorithm used helps to check the stereochemical parameters of the protein models and for all the models more than 90% of the residues were found to be in the allowed region of the Ramachandran Plot (Figure S8). Though there is a possibility that one of these quality estimation methods gives a low score when compared to others, but none of the models are excluded, the scores of each model obtained from these programs are highlighted in appropriate color and appropriately displayed in the database with their guiding values (Table S3).

### Binding Site Identification and comparison

Potential ligand and DNA-binding sites in the various models were identified through a consensus ranking of PocketDepth [40] (Figure S9), a geometry-based pocket identification method and LIGSITEcsc [57], that analyses conserved surface residues and predicts the functional site located to be around a point without giving any boundary definition to pocket unlike PocketDepth (Figure 4B).All the pockets predicted by PocketDepth within 5 Å zone of LigsiteCSC predicted site were selected. Further, the predicted binding sites were compared to known sites in PDB using PocketMatch [45]. This algorithm, also developed recently by us, compares the binding sites in a frame invariant manner by representing and comparing each site by 90 lists of sorted distances capturing shape and chemical nature of the site. An alignment score between a pair of sites is the net average of the number of matching distance elements in the 90 lists as a fraction of total number of distance elements in the bigger set, for a chosen threshold *τ*, as shown where |*S*| indicates cardinality of the set. 
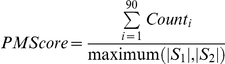



*Count_i_* represents the number of distance elements matched in the 90 lists and represents maximum (|*S*_1_|, |*S*_2_|) the number of distance elements in the larger site (either *S*_1_or *S*_2_) is followed by a ligand assignment to each pocket and hence to a protein, when a high scoring matching pocket is found.

### Structural Motif searches

Sub-structure searches were performed using the ProFunc server's local 3D template searches [58]. The templates are defined as the 3D conformations of 3–6 neighboring amino acid residues. ProFunc has three precompiled databases of templates (Figure S10): first is a manually curated set of enzyme catalytic residues obtained from the Catalytic Site Atlas [49] (CSA); second is an automatically compiled set of templates consisting of groups of three residues interacting with ligands in protein-ligand complexes in the PDB; and the third is the same, but for residues interacting with DNA or RNA. In addition to these searches, ProFunc also returns the best-matching “reverse template” hits. These templates are compiled from the model protein itself and are then searched against a representative subset of PDB structures.

### Quaternary structure

Quaternary structure information for the 1637 templates used for modeling the proteome in this study, were obtained through PQS [59]. Residues present at the interfaces in the templates were compared in the corresponding models. Different types of interactions are calculated in example cases using the PIC server [53]. 35 potential quaternary structures were identified in the modeled proteome as sequential gene-products.

## Supporting Information

Figure S1 (A) Plot showing the distribution of coverage of all protein models. The plot shows majority of proteins having coverage of 90-100%. (B) Distribution of template resolution structures that have been used for modeling, around 80% of the templates have resolution below 3 Å.
(TIF)

Figure S2 (A) Distribution of RMSD between modeled structure and its template is shown. (B) CE Z-score values between the modeled structure and its template has been plotted with majority of them showing score of more than 6. (C) Plot showing the distribution of various folds in the modeled proteome. The number of different folds observed the modeled database is arranged alphabetically on the x-axis, where the y-axis represents the frequency of the corresponding folds.
(TIF)

Figure S3 Modeling and Functional Characterization of Rv1485, Ferrochelatase HEMZ. The homology model was built by utilizing (PDB 1HRK) Human ferrochelatase, chain A (E.C. 4.99.1.1) as template having a sequence identity of 27%. Given (A) is structure based sequence alignment obtained through MUSTANG between the template and the model. Surface cleft analysis is shown (B), the coloring of the surface is based on the volume of the cleft in Å^3^. Residues conserved in the protein are shown on right (C), the residues colored in red are highly conserved and the ones in grey are least conserved.
(TIF)

Figure S4 (A) A superposition of the modeled Rv1752 (in green) and 2 poq (in blue) shows an alignment spanning the C-terminal substrate-binding domain. Residues lining the active site pocket in 2 poq and Rv1752 (in sticks, red in color) are seen to lie in structurally equivalent positions. Conserved sequence motifs in DD and related homologs are partially conserved in Rv1752 (highlighted in yellow). PocketDepth predictions suggest a binding pocket (in gray) that overlaps with the template binding pocket. (B) A superposition of modeled Rv0469 (in green) and 1 kpgA (in orange) shows an appreciable conservation (∼85%) of both substrate binding residues (in blue) and co-factor binding residues (in red). PocketDepth predictions (in grey surface) overlap with the bound ligand in the crystal structure.
(TIF)

Figure S5 MSA of query protein Rv3340 (O5339O) with the top most blast hit against Uniprot. The template 2 ctz A (Q5SK88) is highlighted in red. The CSA residues are marked as circles and the important residues at the binding site are marked as square.
(TIF)

Figure S6 (A) Binding site of Rv1492 (shown as red sticks) predicted (depicted as surface) as the residues that are known to interact with the cofactor Vitamin B12 in the template 1REQ_B are also conserved (shown in green). (B) The residues that are conserved at the interface in the template (green) and the modeled structures (blue) are shown here (i). The superposition of Rv1492 with 1req_b chain residues at the interface. (ii). Superposition of Rv1493 with 1req_A chain residues at the interface. (C) Different types of interaction found at the interface. Sticks in magenta represent the residues from Rv1492 and in blue from Rv1493.(i) Salt-bridges, (ii) Main chain – Main chain interactions, (iii) Side chain - Side chain interactions & (iv) Main chain – Side chain interaction.
(TIF)

Figure S7Flowchart describing the method of comparative modeling used. The DOPE score expression had been mentioned wherein 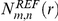 and 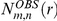 are the numbers of atom type pairs (m,n) at a distance r within [r, r+Δr] for the “interacting” real system and the “non-interacting” reference state respectively.(TIF)

Figure S8 Procheck details along with Ramachandran Plot of all the models in the structural proteome and the various plots obtained from Procheck are shown below as example for Rv0001.
(TIF)

Figure S9 Flow chart depicting the basic idea behind the PocketDepth algorithm. The algorithm involves construction of grid having cell dimension of 1 Å to enclose the query protein. The constructed grid-cells are then labeled to distinguish between internal, external and surface grid cells. Grid bars are constructed across surface cells after finding connected list of surface cells and defining putative boundary atoms. Depth-factor values are calculated for each cell depending upon number of grid-bars that pass across them, later the cells are clustered using DBSCAN algorithm to report the clusters of predicted binding site.
(TIF)

Figure S10 A schematic representation of the residue template-matching methods used in ProFunc. At the top are the three precompiled template databases. The Catalytic Site Atlas (CSA) templates correspond to catalytic residues found in enzymes. These templates are manually curated and, currently, the database contains templates corresponding to 584 different E.C. numbers. The ligand- and DNA-binding templates are automatically derived from protein/small-molecule and protein/DNA complexes in the PDB, respectively. There are over 96,000 of the former and over 3,500 of the latter. The red rings and joining lines represent template matches. The bottom half of the figure shows the principle behind the ‘reverse template’ method. These 3-residue templates are generated from the query structure and then scanned against a representative set of PDB structures. To filter out random hits returned by any of the above template methods, the template structure is superposed on the matching structure by fitting the template and matching residues. The quality of the match is assessed according to the numbers of identical and similar residues that are superposed in the neighborhood of the match.
(TIF)

Table S1 Taxonomic distribution of template protein structures used to model the proteome of TB.
(CSV)

Table S2 List of protein models compared to its corresponding experimentally determined structure through structural superposition (DALI). The RMSD between the structures along with z-score, sequence identity of model with its template and coverage of the model is reported. The superposition of structures are also shown, modeled protein is cyan in color whereas corresponding crystal structure is green in color. The models and corresponding crystal structures were also checked for their fold assignments by using 3D-BLAST.
(PDF)

Table S3 The various criteria and cut-offs used in the pipeline have been mentioned along with the statistics.
(DOC)

Table S4 Genome Threader results for the entire proteome reporting the confidence, PDB template ID and SCOP association.
(CSV)

Table S5 Frequency of different folds observed in the modeled proteome is shown.
(CSV)

Table S6 Of the 312 crystal structures taken as the test set for validation, 105 of them are complexes with biologically relevant ligands, forming 179 different binding sites. 154 of these sites in 97 of the proteins were predicted correctly by PocketDepth (as shown in Column 2) and 46 ligand associations that could be predicted using PocketMatch alone (above the threshold used) were all found to be correct. There were of course many other ligand associations that appear correct, but fall below the stringent thresholds chosen in this study and hence have not been counted.
(PDF)

Table S7 The fold information of the crystal structures were obtained from 3D-BLAST. The fold based function annotation for each of the chains of the protein structure was obtained through superfamily database and compared against existing GO functional terms through manual inspection. In the 60% of the cases both the annotation matched and in the remaining 40% of the cases either of the annotations had no detailed functional term associated and hence could not be compared readily.
(XLS)

Table S8 Different co-occurences of domains occurring in the modeled proteome. Frequency of different domain combination observed in the modeled proteome is reported.
(CSV)
