# Supplementary figures and images for: Structural Annotation of Mycobacterium tuberculosis Proteome
Source: PLoS One. 2011 Oct 31;6(10):e27044. doi: 10.1371/journal.pone.0027044 (PMC3205055; doi:10.1371/journal.pone.0027044)

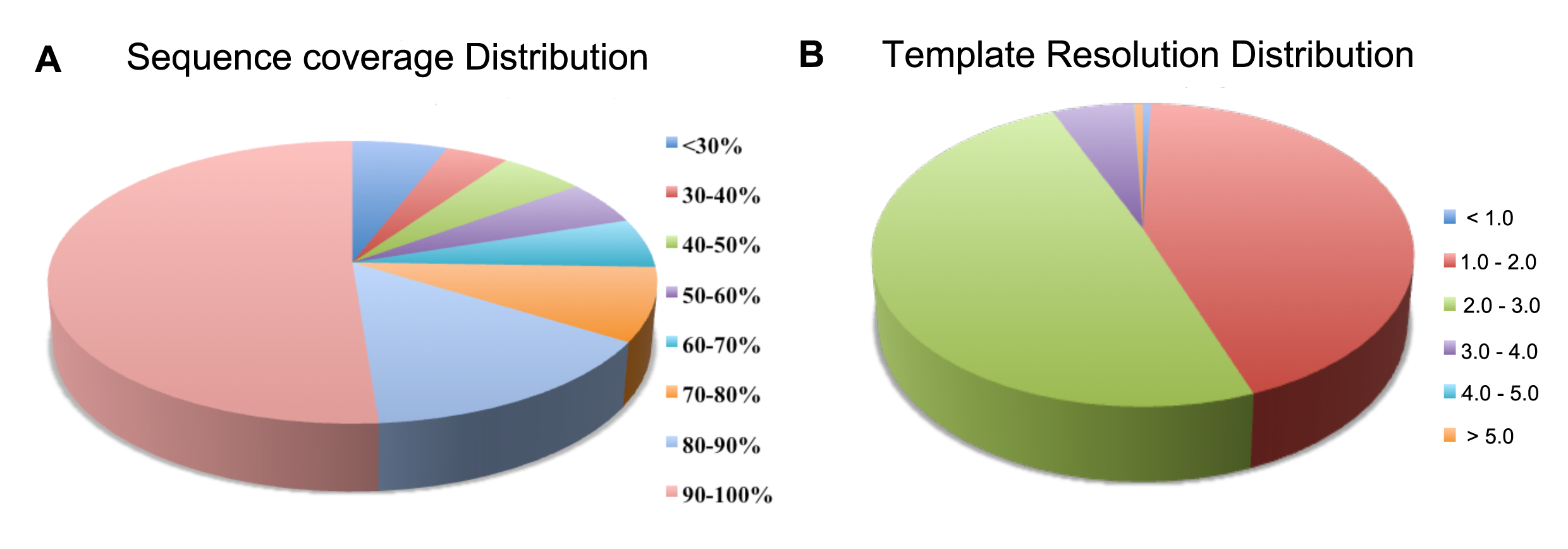

Supplement: Figure S1 — (A) Plot showing the distribution of coverage of all protein models. The plot shows majority of proteins having coverage of 90-100%. (B) Distribution of template resolution structures that have been used for modeling, around 80% of the templates have resolution below 3 Å. (TIF) [file pone.0027044.s001.tif]

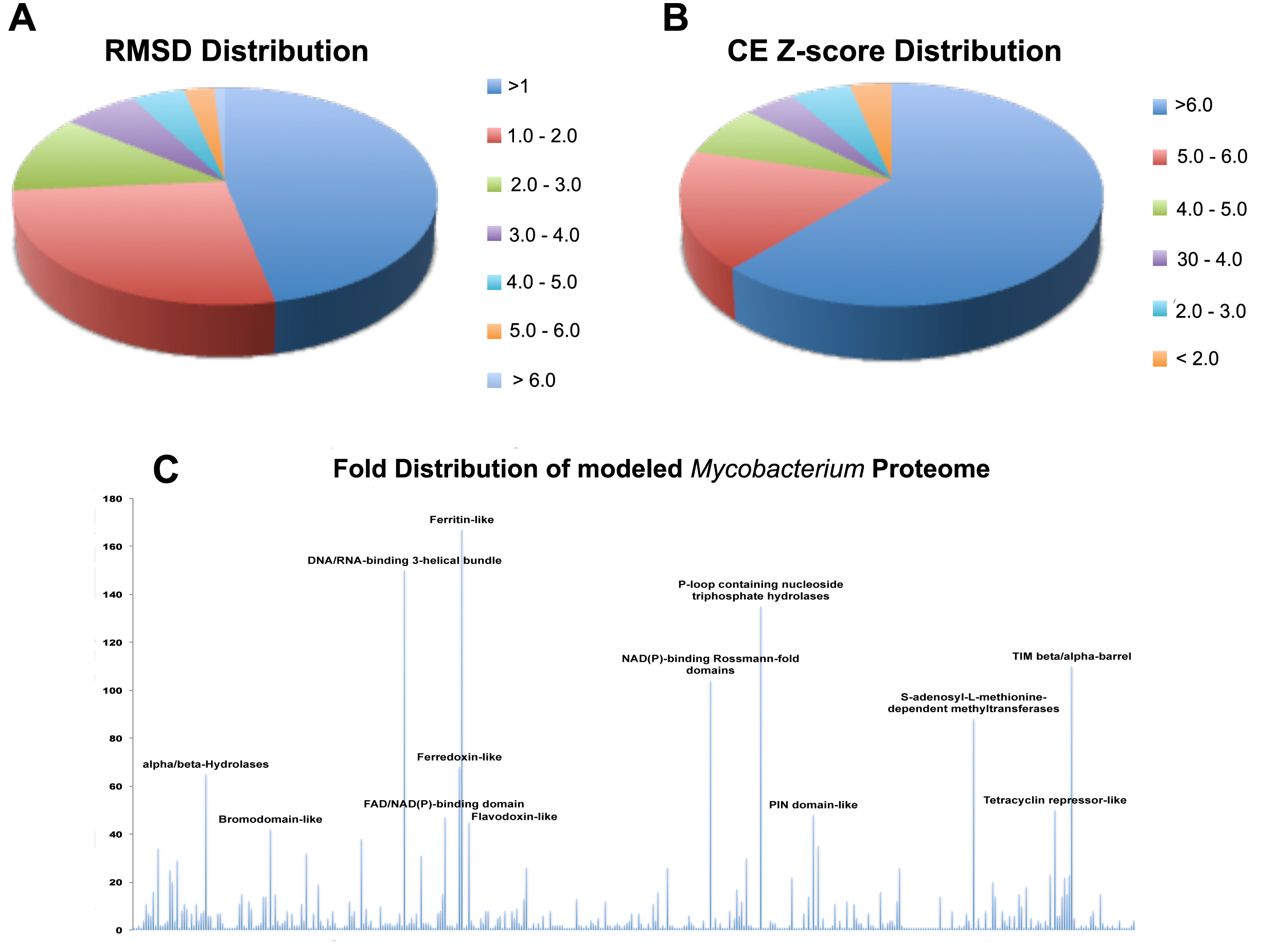

Supplement: Figure S2 — (A) Distribution of RMSD between modeled structure and its template is shown. (B) CE Z-score values between the modeled structure and its template has been plotted with majority of them showing score of more than 6. (C) Plot showing the distribution of various folds in the modeled proteome. The number of different folds observed the modeled database is arranged alphabetically on the x-axis, where the y-axis represents the frequency of the corresponding folds. (TIF) [file pone.0027044.s002.tif]

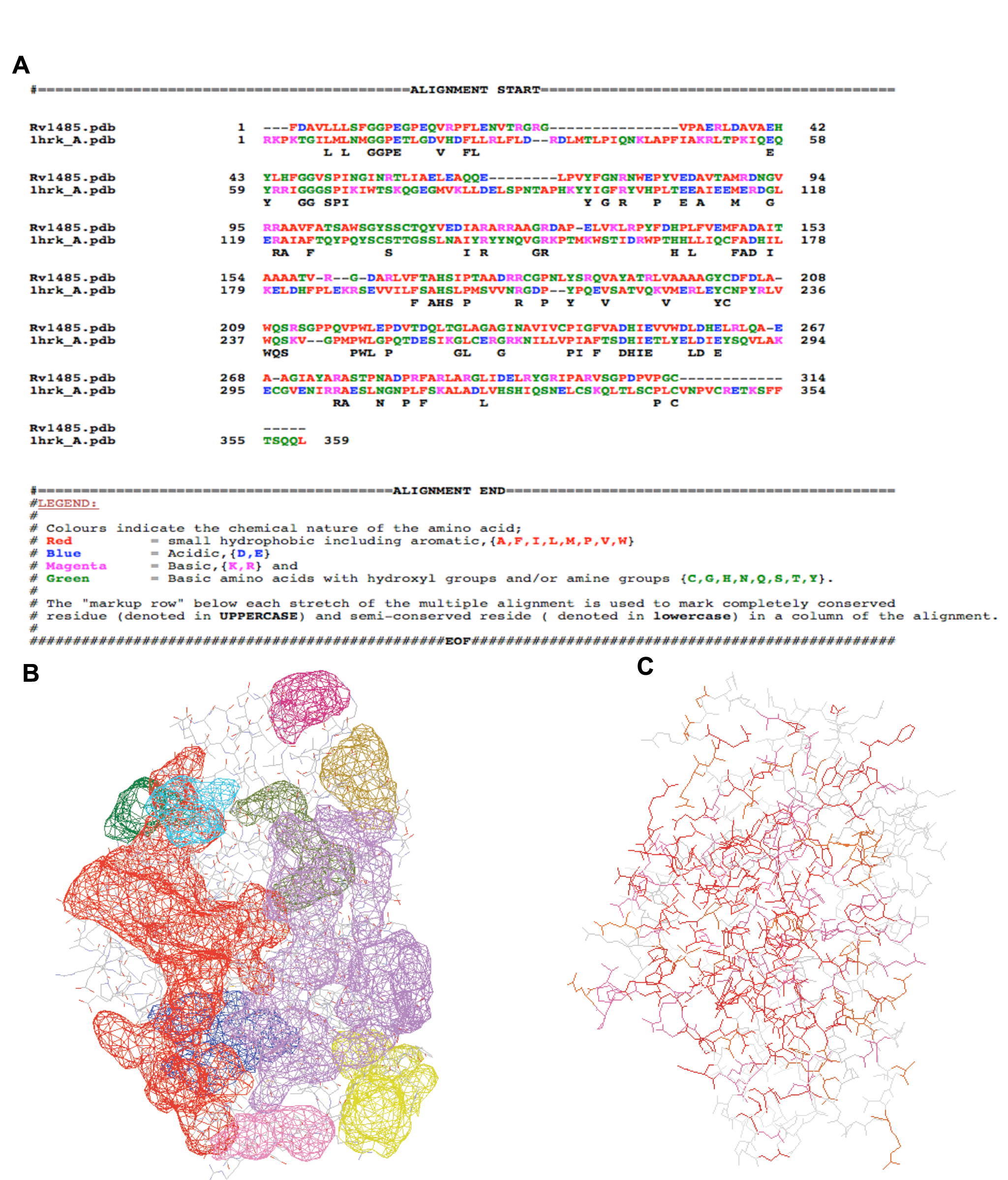

Supplement: Figure S3 — Modeling and Functional Characterization of Rv1485, Ferrochelatase HEMZ. The homology model was built by utilizing (PDB 1HRK) Human ferrochelatase, chain A (E.C. 4.99.1.1) as template having a sequence identity of 27%. Given (A) is structure based sequence alignment obtained through MUSTANG between the template and the model. Surface cleft analysis is shown (B), the coloring of the surface is based on the volume of the cleft in Å3. Residues conserved in the protein are shown on right (C), the residues colored in red are highly conserved and the ones in grey are least conserved. (TIF) [file pone.0027044.s003.tif]

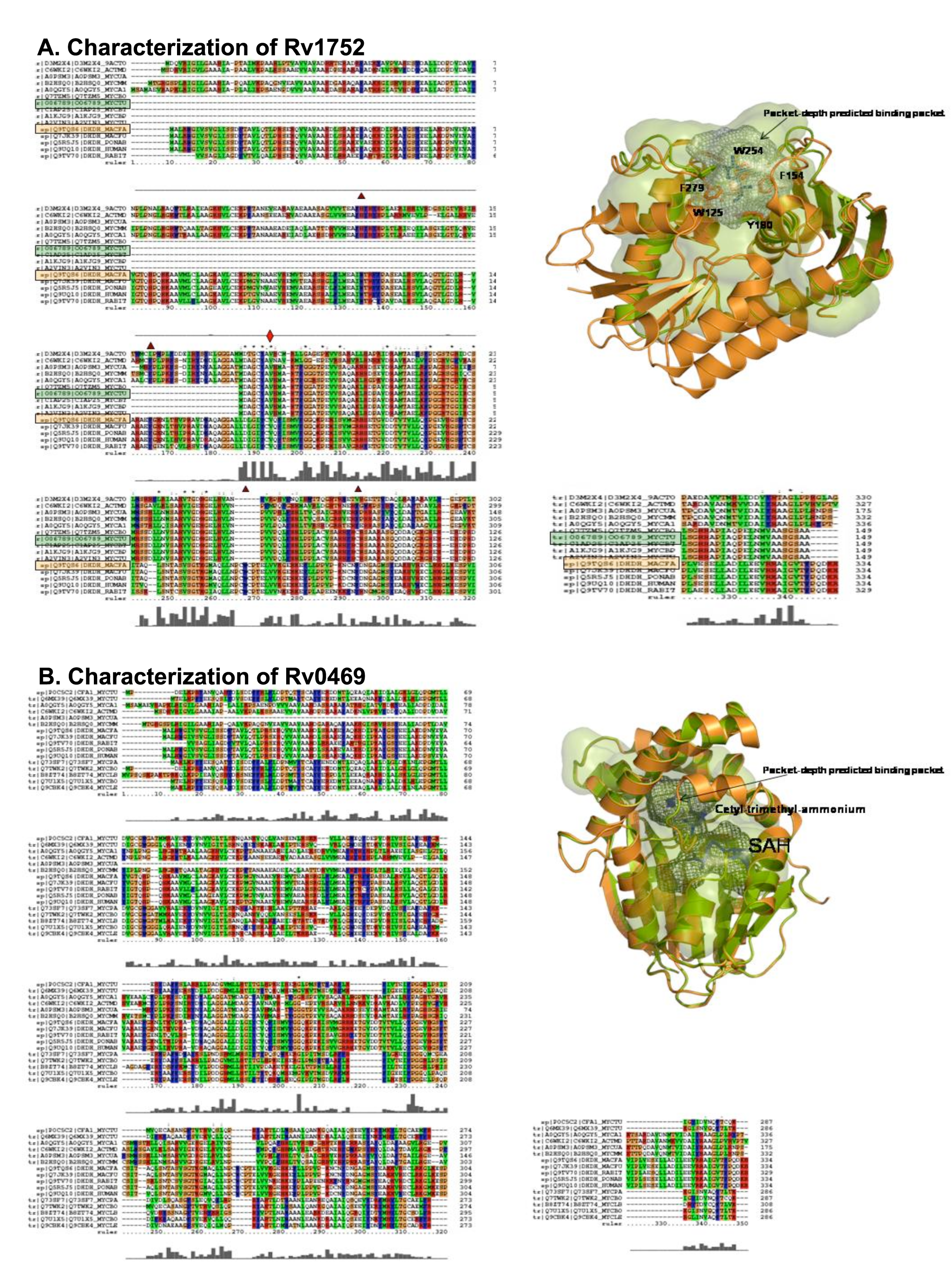

Supplement: Figure S4 — (A) A superposition of the modeled Rv1752 (in green) and 2 poq (in blue) shows an alignment spanning the C-terminal substrate-binding domain. Residues lining the active site pocket in 2 poq and Rv1752 (in sticks, red in color) are seen to lie in structurally equivalent positions. Conserved sequence motifs in DD and related homologs are partially conserved in Rv1752 (highlighted in yellow). PocketDepth predictions suggest a binding pocket (in gray) that overlaps with the template binding pocket. (B) A superposition of modeled Rv0469 (in green) and 1 kpgA (in orange) shows an appreciable conservation (∼85%) of both substrate binding residues (in blue) and co-factor binding residues (in red). PocketDepth predictions (in grey surface) overlap with the bound ligand in the crystal structure. (TIF) [file pone.0027044.s004.tif]

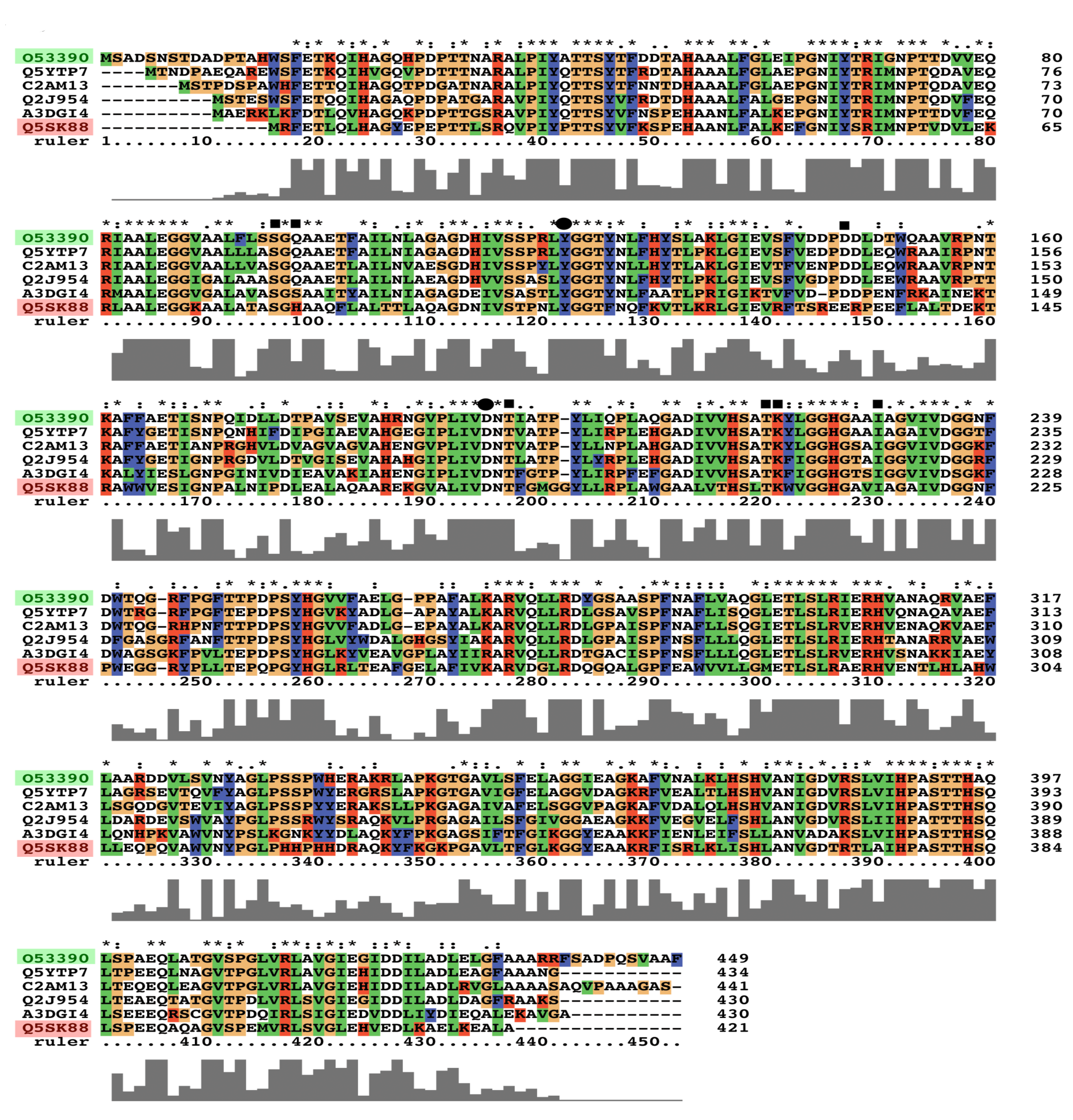

Supplement: Figure S5 — MSA of query protein Rv3340 (O5339O) with the top most blast hit against Uniprot. The template 2 ctz A (Q5SK88) is highlighted in red. The CSA residues are marked as circles and the important residues at the binding site are marked as square. (TIF) [file pone.0027044.s005.tif]

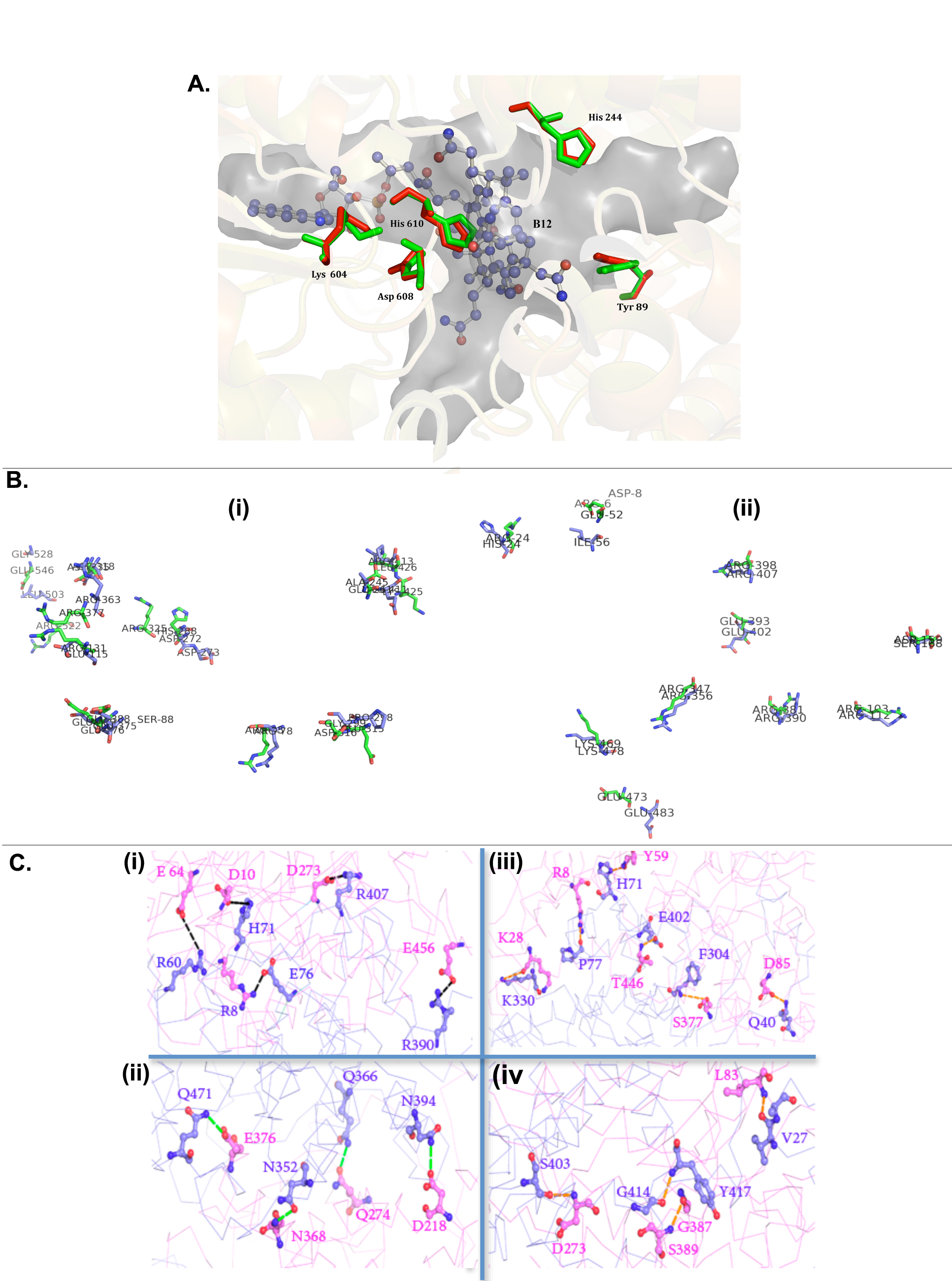

Supplement: Figure S6 — (A) Binding site of Rv1492 (shown as red sticks) predicted (depicted as surface) as the residues that are known to interact with the cofactor Vitamin B12 in the template 1REQ_B are also conserved (shown in green). (B) The residues that are conserved at the interface in the template (green) and the modeled structures (blue) are shown here (i). The superposition of Rv1492 with 1req_b chain residues at the interface. (ii). Superposition of Rv1493 with 1req_A chain residues at the interface. (C) Different types of interaction found at the interface. Sticks in magenta represent the residues from Rv1492 and in blue from Rv1493.(i) Salt-bridges, (ii) Main chain – Main chain interactions, (iii) Side chain - Side chain interactions & (iv) Main chain – Side chain interaction. (TIF) [file pone.0027044.s006.tif]

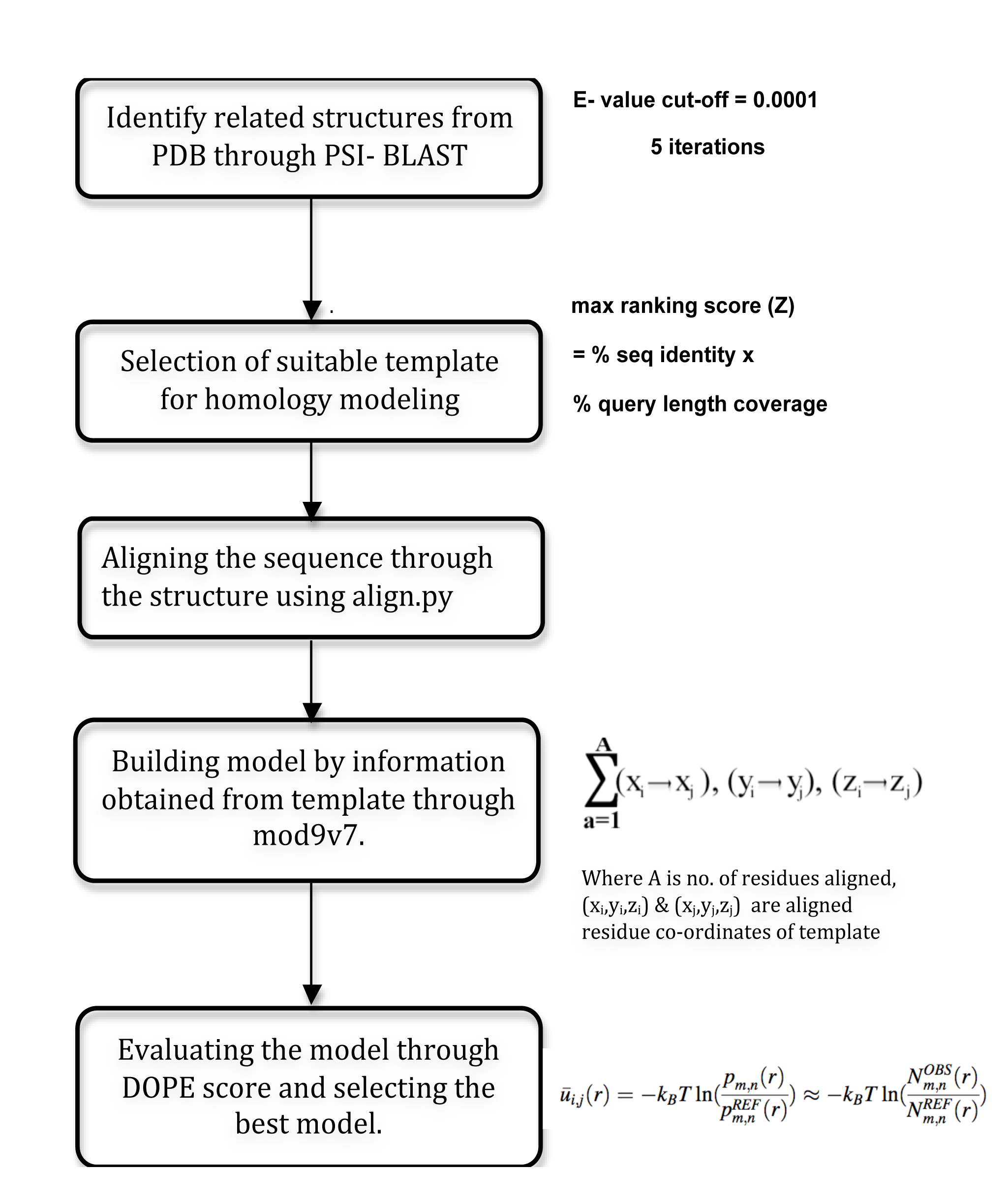

Supplement: Figure S7 — Flowchart describing the method of comparative modeling used. The DOPE score expression had been mentioned wherein and are the numbers of atom type pairs (m,n) at a distance r within [r, r+Δr] for the “interacting” real system and the “non-interacting” reference state respectively. (TIF) [file pone.0027044.s007.tif]

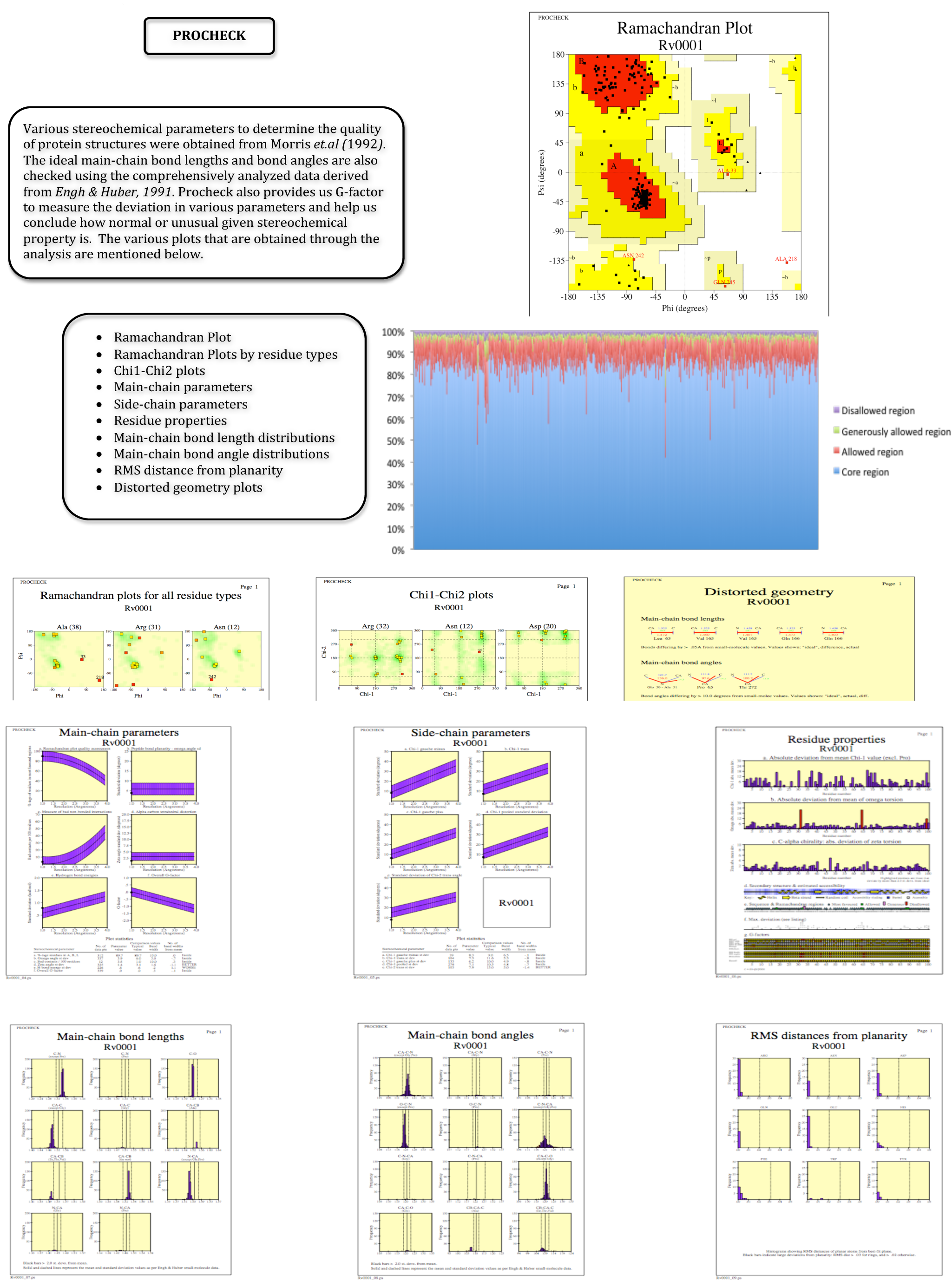

Supplement: Figure S8 — Procheck details along with Ramachandran Plot of all the models in the structural proteome and the various plots obtained from Procheck are shown below as example for Rv0001. (TIF) [file pone.0027044.s008.tif]

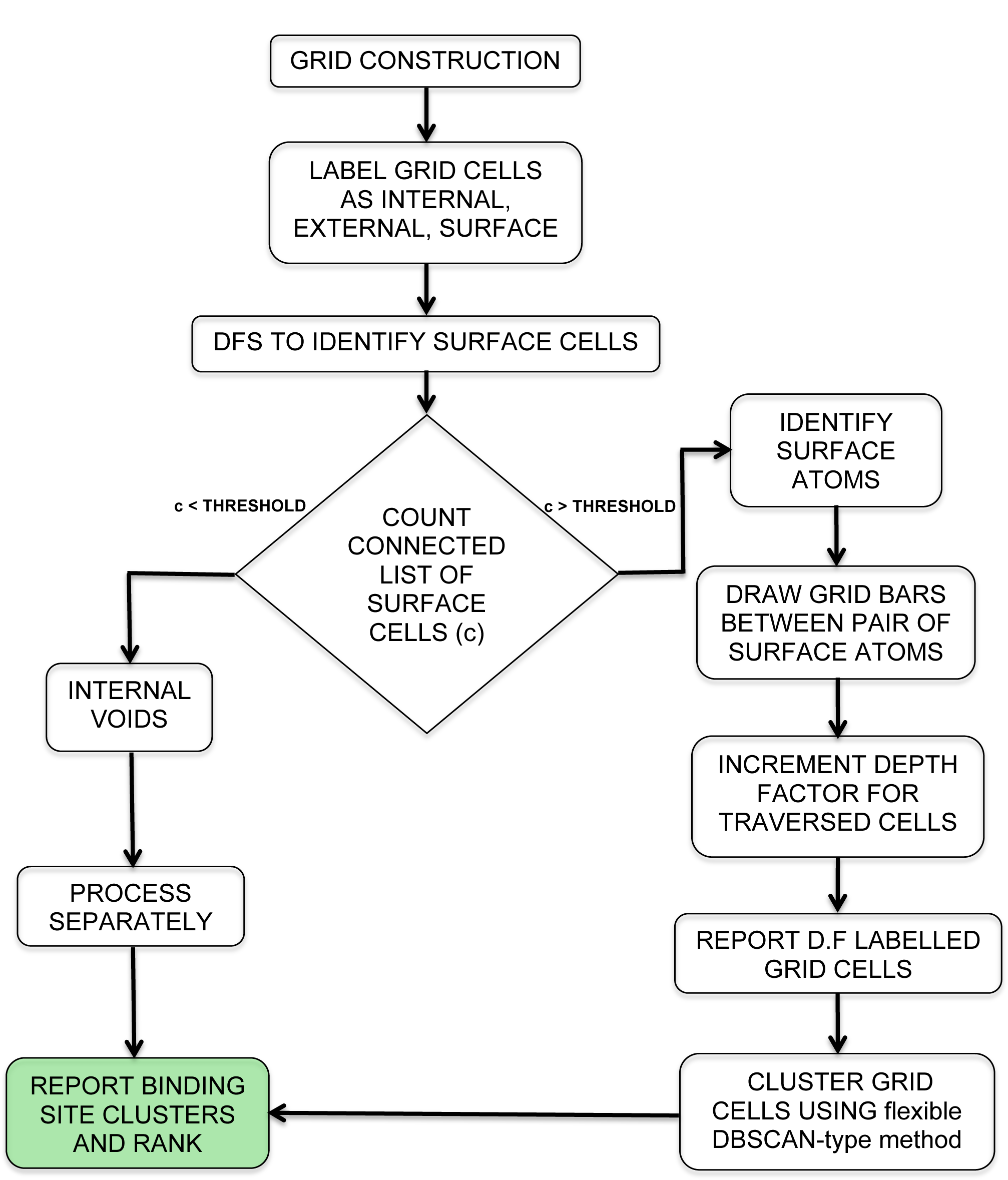

Supplement: Figure S9 — Flow chart depicting the basic idea behind the PocketDepth algorithm. The algorithm involves construction of grid having cell dimension of 1 Å to enclose the query protein. The constructed grid-cells are then labeled to distinguish between internal, external and surface grid cells. Grid bars are constructed across surface cells after finding connected list of surface cells and defining putative boundary atoms. Depth-factor values are calculated for each cell depending upon number of grid-bars that pass across them, later the cells are clustered using DBSCAN algorithm to report the clusters of predicted binding site. (TIF) [file pone.0027044.s009.tif]

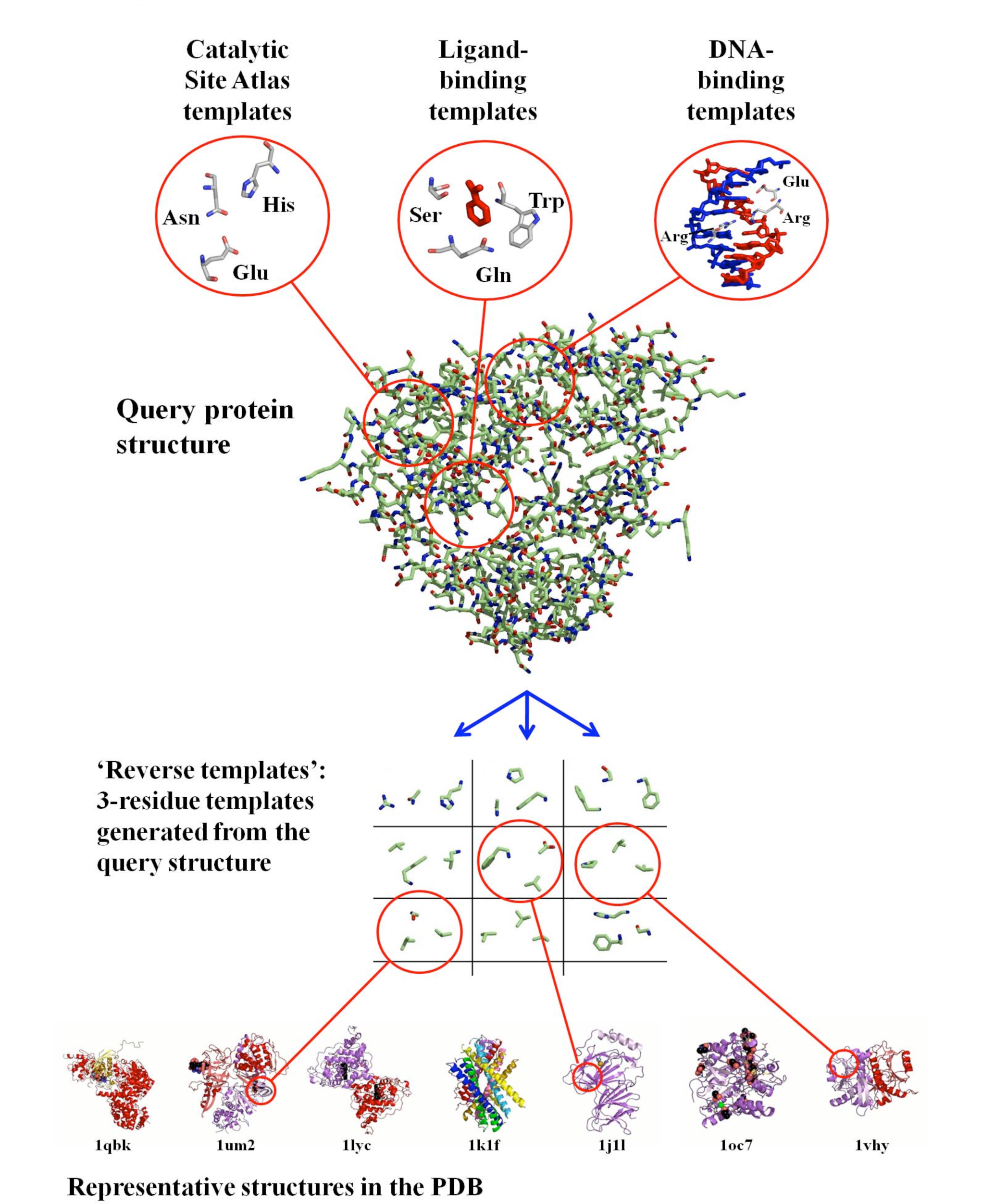

Supplement: Figure S10 — A schematic representation of the residue template-matching methods used in ProFunc. At the top are the three precompiled template databases. The Catalytic Site Atlas (CSA) templates correspond to catalytic residues found in enzymes. These templates are manually curated and, currently, the database contains templates corresponding to 584 different E.C. numbers. The ligand- and DNA-binding templates are automatically derived from protein/small-molecule and protein/DNA complexes in the PDB, respectively. There are over 96,000 of the former and over 3,500 of the latter. The red rings and joining lines represent template matches. The bottom half of the figure shows the principle behind the ‘reverse template’ method. These 3-residue templates are generated from the query structure and then scanned against a representative set of PDB structures. To filter out random hits returned by any of the above template methods, the template structure is superposed on the matching structure by fitting the template and matching residues. The quality of the match is assessed according to the numbers of identical and similar residues that are superposed in the neighborhood of the match. (TIF) [file pone.0027044.s010.tif]
