# Supplementary material for: Structural Annotation of Mycobacterium tuberculosis Proteome
Source: PLoS One. 2011 Oct 31;6(10):e27044. doi: 10.1371/journal.pone.0027044 (PMC3205055; doi:10.1371/journal.pone.0027044)
Supplement: Table S2 — List of protein models compared to its corresponding experimentally determined structure through structural superposition (DALI). The RMSD between the structures along with z-score, sequence identity of model with its template and coverage of the model is reported. The superposition of structures are also shown, modeled protein is cyan in color whereas corresponding crystal structure is green in color. The models and corresponding crystal structures were also checked for their fold assignments by using 3D-BLAST. (PDF) [file pone.0027044.s012.pdf]

Sl. No	Gene Name	Superpose	Z-score	RMSD(Å)	% Seq ID	% Covrg	Fold
1	Rv0006	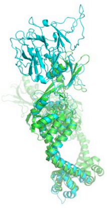	38.7	3.6	34	86.4	✓ fold:e.11.1.1; e-value:e-106
2	Rv0016c	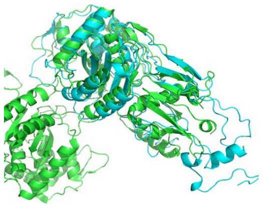	36	2.6	19	93.89	✓ fold:e.3.1.1; e-value:e-124
3	Rv0033	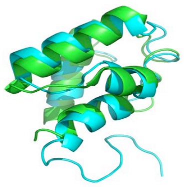	8.6	2.1	30	95.4	✓ fold:a.28.1.1; e-value:1e-33
4	Rv0054	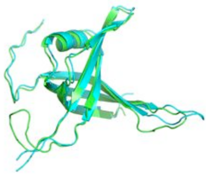	18	1.4	97	71.34	✓ fold:b.40.4.3; e-value:3e-40
5	Rv0098	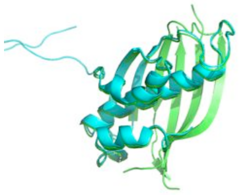	15.5	0.2	84.47	67%	
6	Rv0130	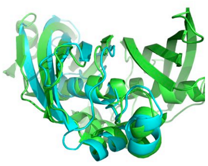	14.7	2.2	30	89.4	✓ fold:d.38.1.4; e-value:7e-51

**7**	**Rv0223c**	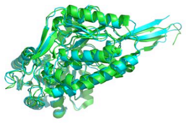	**54**	**1.8**	**38**	**98.36**	✓ fold:c.82.1.1; e-value:0.0
**8**	**Rv0233**	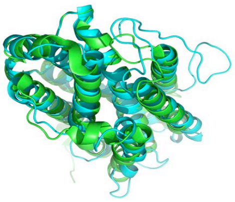	**27.1**	**2.1**	**22**	**89.17**	✓ fold:a.25.1.2; e-value:e-103
**9**	**Rv0287**	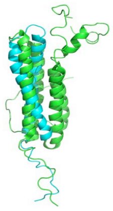	**2.7**	**4.6**	**35**	**87.63**	
**10**	**Rv0288**	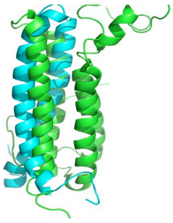	**3.4**	**5.6**	**21**	**97.92**	
**11**	**Rv0321**	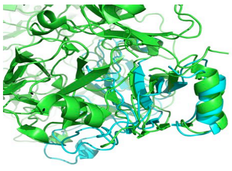	**20.6**	**3.3**	**44**	**96.84**	✓ fold:b.85.4.1; e-value:4e-86
**12**	**Rv0363c**	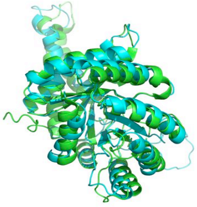	**40.1**	**1.9**	**41**	**97.09**	✓ fold:c.1.10.2; e-value:e-155

13	Rv0371c	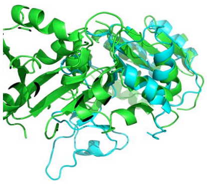	13.9	2.5	19	97.46	✔ fold:c.68.1.5; e-value:1e-18
14	Rv0391	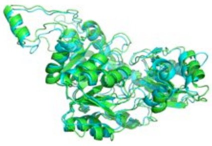	50	1.6	40	94.58	✔ fold:c.67.1.3; e-value:0.0
15	Rv0407	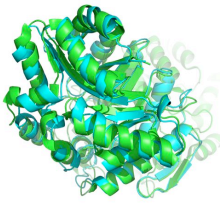	45.3	1.7	34	98.21	✔ fold:c.1.16.3; e-value:e-177
16	Rv0410c	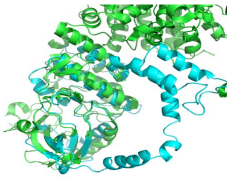	16.6	3.5	23	45.33	✔ fold:d.144.1.7; e-value:4e-66
17	Rv0429c	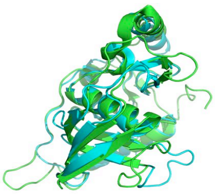	20.7	1.8	41	89.34	✔ fold:d.167.1.1; e-value:2e-69
18	Rv0554	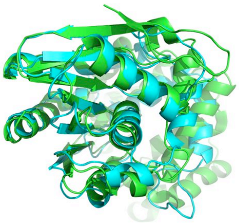	28.4	2.2	28	98.09	✔ fold:c.69.1.10; e-value:5e-59

19	Rv0627	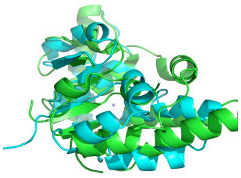	12.4	3.1	20	97.78	
20	Rv0735	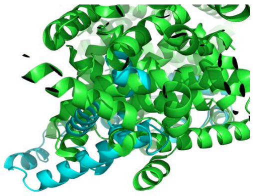	9.1	2	19	96.05	
21	Rv0802c	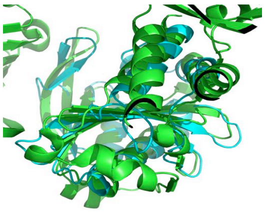	17.4	2.5	25	81.65	✓ fold:d.108.1.1; e-value:3e-62
22	Rv0805	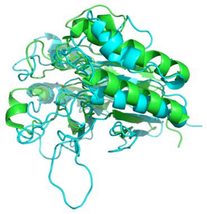	18.8	3	18	93.08	✓ fold:d.159.1.11; e-value:7e-12
23	Rv0813c	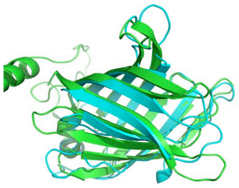	16.8	2.3	33	72.12	✓ fold:b.60.1.8; e-value:3e-45
24	Rv0815c	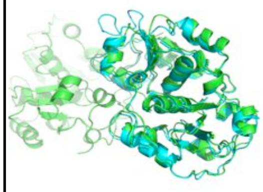	38.6	1.3	51		✓ fold:c.46.1.2; e-value:4e-79

**25**	**Rv0844c**	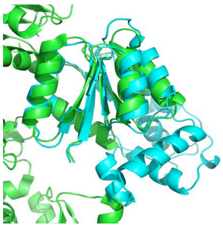	**16.9**	**1.8**	**31**	**96.3**	✓<br>fold:c.23.1.1;<br>e-value:7e-56
**26**	**Rv0855**	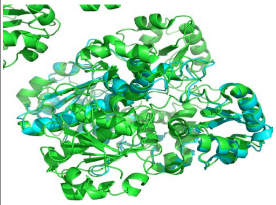	**39.9**	**1.4**	**59**	**95.82**	✓<br>fold:c.123.1.1;<br>e-value:0.0
**27**	**Rv0858c**	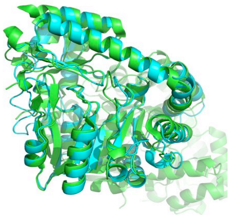	**49.3**	**1.8**	**45**	**96.47**	✓<br>fold:c.67.1.1;<br>e-value:e-162
**28**	**Rv0865**	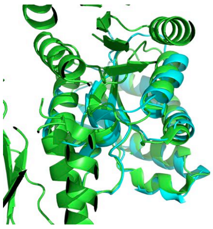	**24.5**	**1.5**	**36**	**95.63**	✓<br>fold:c.57.1.1;<br>e-value:4e-69
**29**	**Rv0884c**	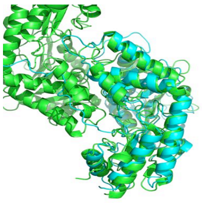	**39**	**2.3**	**20**	**99.2**	✓<br>fold:c.67.1.4;<br>e-value:1e-46
**30**	**Rv0899**	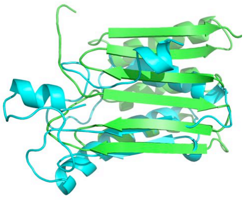	**1.1**	**3**	**36**	**34.05**	

31	Rv0905	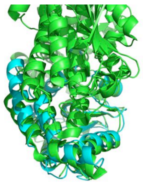	25.6	2.1	33	98.35	✓ fold:c.14.1.3; e-value:3e-91
32	Rv0946c	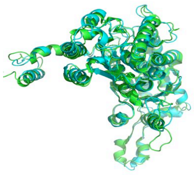	57.6	1.5	50	96.56	✓ fold:c.80.1.2; e-value:0.0
33	Rv0948c	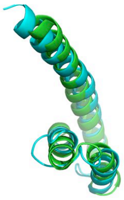	8.2	2.2	29	71.43	✓ fold:a.130.1.1; e-value:1e-16
34	Rv0956	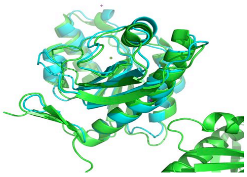	26.8	1.9	35	89.77	✓ fold:c.65.1.1; e-value:e-100
35	Rv1014c	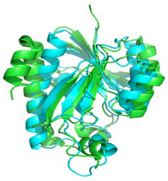	19.4	2.9	43	92.67	✓ fold:c.56.3.1; e-value:4e-96
36	Rv1018c	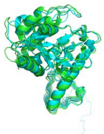	43.9	1.3	43	91.52	✓ fold:c.68.1.5; e-value:e-134
37	Rv1070c	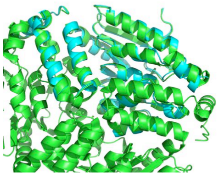	38.3	0.9	50	98.83	✓ fold:c.14.1.3; e-value:e-134

38	Rv1086	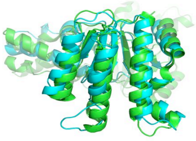	25.5	2.3	34	85.11	✓ fold:c.101.1.1; e-value:1e-90
39	Rv1093	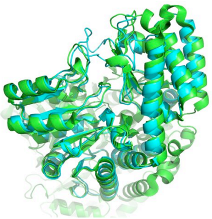	54	1.3	58	95.07	✓ fold:c.67.1.4; e-value:0.0
40	Rv1098c	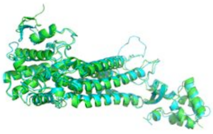	51.7	1.9	50	95.99	✓ fold:a.127.1.1; e-value:0.0
41	Rv1131	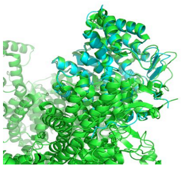	56.4	1.4	60	92.62	✓ fold:a.103.1.1; e-value:0.0
42	Rv1201c	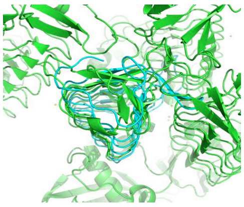	8.3	3.2	22	43.22	✓ fold:b.81.1.2; e-value:2e-28
43	Rv1207	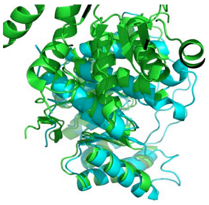	25.5	1.9	34	86.48	✓ fold:c.1.21.1; e-value:e-139
44	Rv1208	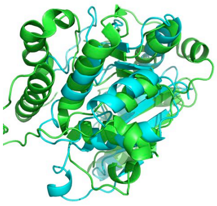	13	3.4	15	59.88	

45	Rv1256c	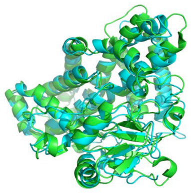	42	2.5	31	98.52	✔ fold:a.104.1.1; e-value:e-104
46	Rv1295	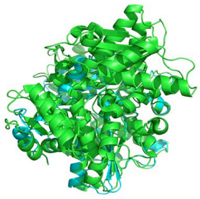	49.6	1.9	59	96.11	✔ fold:c.79.1.1; e-value:0.0
47	Rv1326c	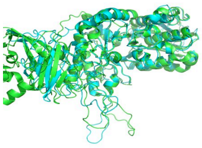	43.8	1.3	52	83.17	✔ fold:c.1.8.1; e-value:0.0
48	Rv1335	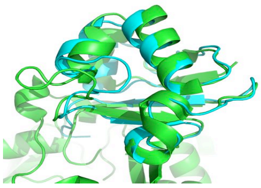	14.1	2	30	95.7	
49	Rv1336	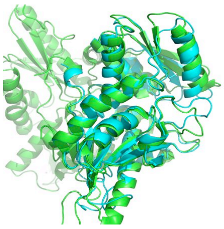	41	1.8	42	92.88	
50	Rv1340	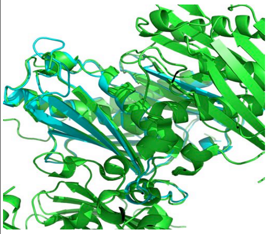	36.9	1.2	57	91.89	✔ fold:d.14.1.4; e-value:2e-77
51	Rv1404	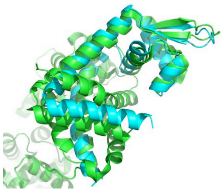	14.5	2.9	23	89.38	✔ fold:a.4.5.28; e-value:7e-60

**52**	**Rv1411c**	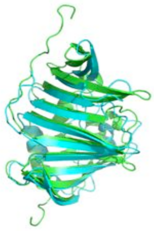	**17.6**	**2.8**	**32**	**81.36**	✓ fold:b.125.1.3; e-value:6e-66
**53**	**Rv1438**	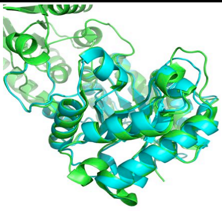	**38.4**	**1.4**	**50**	**96.55**	✓ fold:c.1.1.1; e-value:e-101
**54**	**Rv1445c**	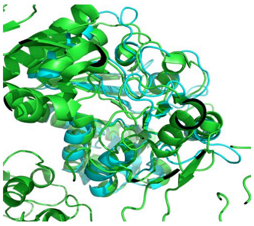	**29.3**	**2.1**	**32**	**96.76**	✓ fold:c.124.1.1; e-value:4e-49
**55**	**Rv1496**	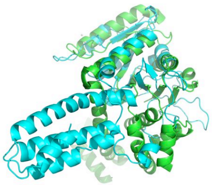	**7.8**	**3.5**	**21**	**95.81**	✓ fold:c.37.1.10; e-value:3e-10
**56**	**Rv1568**	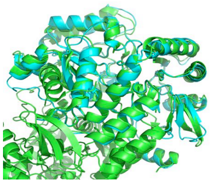	**60.2**	**1.2**	**50**	**96.8**	✓ fold:c.67.1.4; e-value:0.0
**57**	**Rv1570**	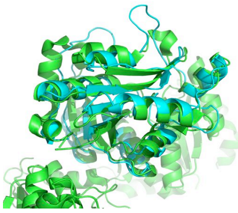	**24.6**	**2.4**	**26**	**96.9**	✓ fold:c.37.1.10; e-value:4e-76
**58**	**Rv1612**	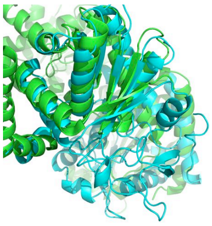	**21.8**	**5**	**58**	**90.76**	✓ fold:c.79.1.1; e-value:0.0

**59**	**Rv1652**	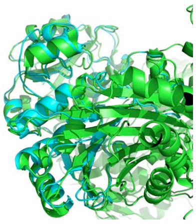	**46.1**	**1.6**	**39**	**97.16**	✓ fold:d.81.1.1; e-value:1e-85
**60**	**Rv1653**	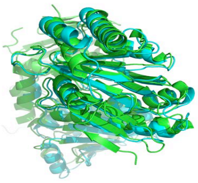	**21.1**	**1.7**	**34**	**94.31**	✓ fold:d.154.1.2; e-value:e-173
**61**	**Rv1656**	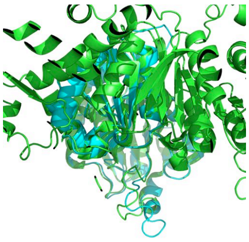	**44.5**	**1.4**	**50**	**98.05**	✓ fold:c.78.1.1; e-value:3e-87
**62**	**Rv1657**	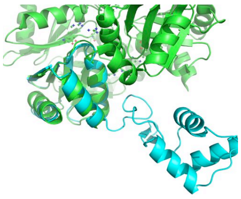	**12.9**	**1.7**	**33**	**88.82**	✓ fold:d.74.2.1; e-value:5e-28
**63**	**Rv1689**	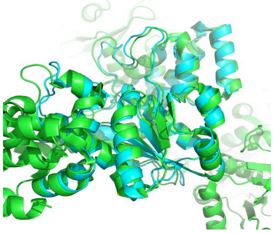	**37.4**	**1.7**	**55**	**74.53**	✓ fold:c.26.1.1; e-value:e-161
**64**	**Rv1826**	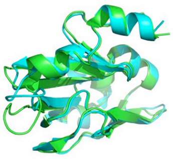	**23.4**	**1.1**	**53**	**97.01**	✓ fold:b.84.1.1; e-value:8e-67
**65**	**Rv1827**	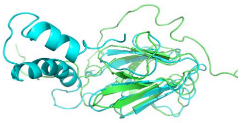	**8.6**	**2.6**	**26**	**91.36**	

66	Rv1846c	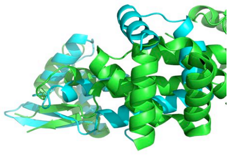	11.7	6.3	21	85.51	✓ fold:a.4.5.39; e-value:2e-45
67	Rv1876	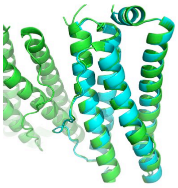	25.5	0.7	48	96.86	
68	Rv1938	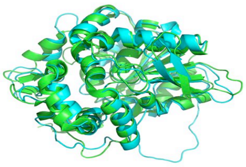	39.2	2	35	97.75	
69	Rv1941	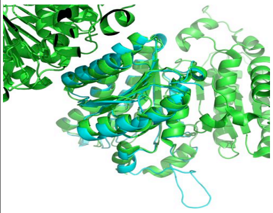	35.4	1.5	37	97.27	✓ fold:c.2.1.2; e-value:e-140
70	Rv1985c	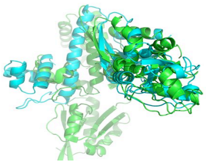	13.2	4.8	21	93.73	✓ fold:c.94.1.1; e-value:3e-94
71	Rv1994c	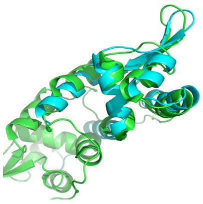	10.9	2	31	77.97	
72	Rv2027c	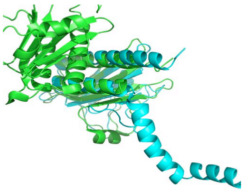	17	2.2	19	32.46	✓ fold:d.110.2.1; e-value:2e-65

**73**	**Rv2043c**	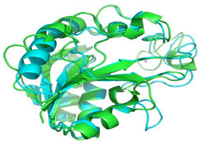	**24.4**	**2**	**39**	**96.77**	✓ fold:c.33.1.3; e-value:7e-77
**74**	**Rv2069**	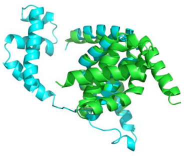	**13.7**	**1.4**	**30**	**95.68**	✓ fold:a.177.1.1; e-value:4e-45
**75**	**Rv2158c**	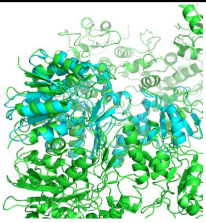	**41**	**2.5**	**38**	**86.36**	
**76**	**Rv2202c**	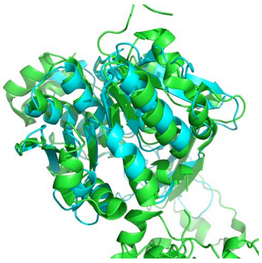	**29**	**2.7**	**23**	**94.75**	✓ fold:c.72.1.1; e-value:e-103
**77**	**Rv2210c**	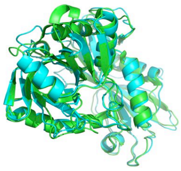	**40.5**	**1.8**	**39**	**95.38**	✓ fold:e.17.1.1; e-value:e-167
**78**	**Rv2243**	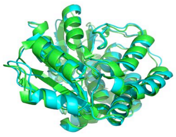	**45.6**	**1.3**	**54**	**99.67**	✓ fold:c.19.1.1; e-value:3e-62
**79**	**Rv2266**	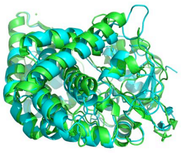	**44.8**	**2.1**	**32**	**94.63**	✓ fold:a.104.1.1; e-value:e-110

**80**	**Rv2334**	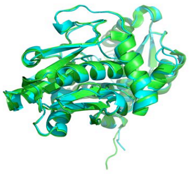	**48.5**	**0.8**	**58**	**97.42**	✓ fold:c.79.1.1; e-value:e-166
**81**	**Rv2359**	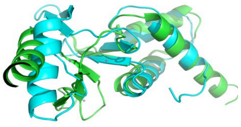	**11.8**	**4.7**	**34**	**90.77**	✓ fold:a.4.5.42; e-value:3e-53
**82**	**Rv2361c**	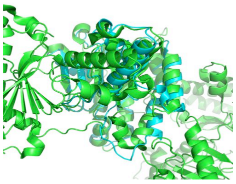	**26.4**	**1.9**	**38**	**76.69**	✓ fold:c.101.1.1; e-value:e-108
**83**	**Rv2386c**	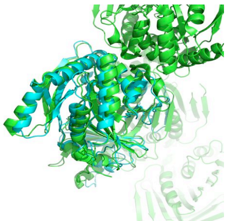	**47.3**	**2.3**	**38**	**92**	✓ fold:d.161.1.1; e-value:0.0
**84**	**Rv2436**	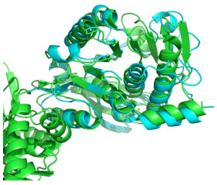	**37.8**	**1.7**	**38**	**94.41**	✓ fold:c.72.1.1; e-value:e-117
**85**	**Rv2447c**	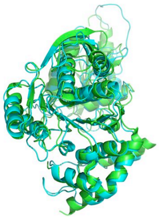	**44.2**	**2.8**	**34**	**93.84**	✓ fold:c.72.2.2; e-value:e-132

**86**	**Rv2495c**	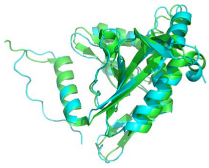	**29.6**	**2**	**36**	**60.05**	fold:c.43.1.1; e-value:e-114
**87**	**Rv2523c**	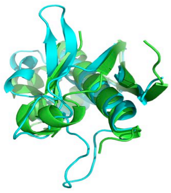	**10.4**	**3.1**	**31**	**96.92**	fold:d.150.1.2; e-value:4e-44
**88**	**Rv2579**	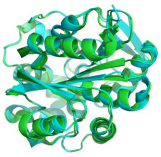	**52.6**	**0.5**	**69**	**98.67**	fold:c.69.1.8; e-value:0.0
**89**	**Rv2593c**	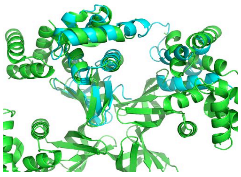	**23.1**	**2.7**	**81**	**99.49**	fold:b.40.4.2; e-value:1e-29
**90**	**Rv2623**	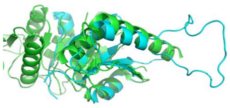	**11.6**	**2.1**	**30**	**48.82**	fold:c.26.2.4; e-value:1e-34
**91**	**Rv2701c**	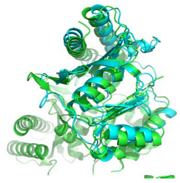	**29.8**	**2**	**32**	**93.45**	fold:e.7.1.1; e-value:e-112
**92**	**Rv2704**	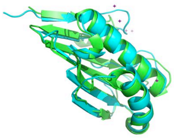	**18.4**	**1.8**	**29**	**85.21**	fold:d.79.1.1; e-value:1e-54

93	Rv2726c	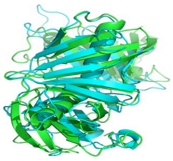	25.7	3.4	26	97.23	✓ fold:d.21.1.1; e-value:6e-58
94	Rv2737c	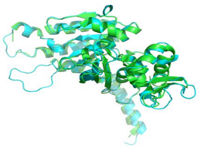	42.3	0.8	81	42.53	✓ fold:c.37.1.11; e-value:e-126
95	Rv2780	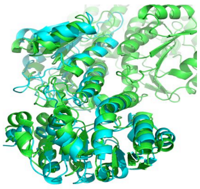	46.2	3	49	96.77	✓ fold:c.23.12.2; e-value:9e-78
96	Rv2844	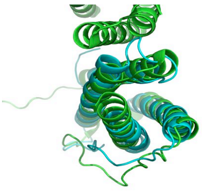	15.8	2.3	21	79.63	✓ fold:a.25.1.9; e-value:7e-23
97	Rv2865	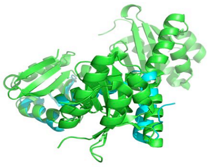	8	2.2	26	84.95	✓ fold:d.306.1.1; e-value:9e-30
98	Rv2866	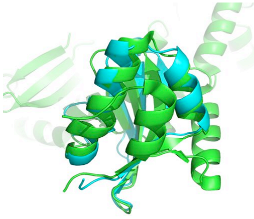	10.8	2.2	26	95.4	
99	Rv2874	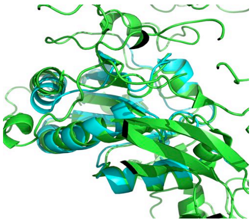	17.5	2.1	24	20.86	✓ fold:c.47.1.10; e-value:1e-59

100	Rv2883c	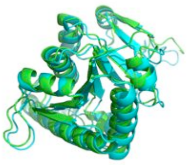	35.6	1.4	89.27	55	✓ fold:c.73.1.3; e-value:e-123
101	Rv2919c	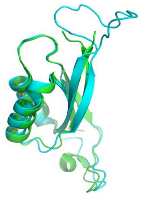	14.8	1.5	62	99.11	✓ fold:d.58.5.1; e-value:3e-58
102	Rv2933	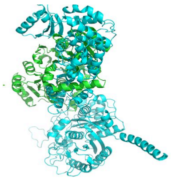	1.4	4.3	43	41.41	
103	Rv2941	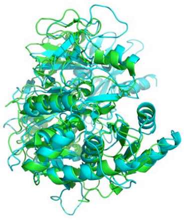	32.9	2.8	19	98.45	✓ fold:e.23.1.1; e-value:e-180
104	Rv2976c	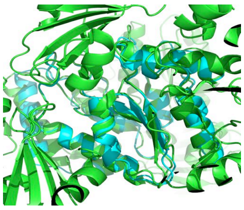	32.4	1.4	49	93.83	✓ fold:c.18.1.1; e-value:4e-77
105	Rv2981c	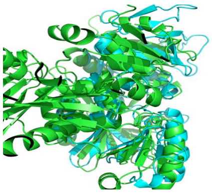	32.1	3.3	36	95.71	✓ fold:d.142.1.1; e-value:e-100
106	Rv2986c	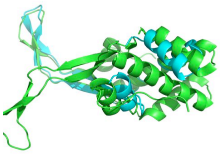	10.1	2.5	44	41.59	✓ fold:a.55.1.1; e-value:8e-38

**107**	**Rv2987c**	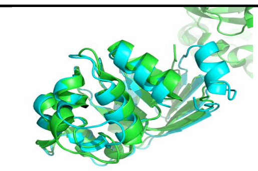	**24**	**1.7**	**41**	**84.34**	✓<br>fold:c.8.2.1;<br>e-value:2e-26
**108**	**Rv2992c**	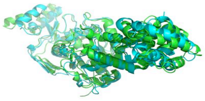	**38.7**	**3.5**	**37**	**97.55**	✓<br>fold:c.26.1.1;<br>e-value:e-155
**109**	**Rv3019c**	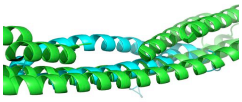	**1.8**	**3.4**	**24**	**97.92**	
**110**	**Rv3020c**	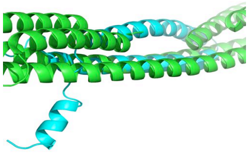	**2.2**	**4**	**31**	**97.94**	
**111**	**Rv3132c**	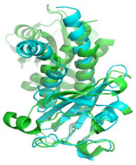	**16.3**	**2.4**	**24**	**28.89**	✓<br>fold:d.110.2.1;<br>e-value:4e-68
**112**	**Rv3248c**	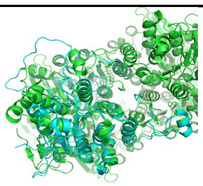	**49.7**	**1.8**	**60**	**96.57**	✓<br>fold:c.23.12.3;<br>e-value:5e-62
**113**	**Rv3250c**	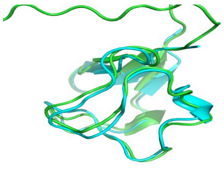	**8.8**	**1**	**51**	**86.67**	

114	Rv3279c	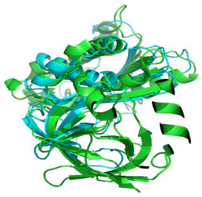	26.3	2.2	32	96.24	
115	Rv3283	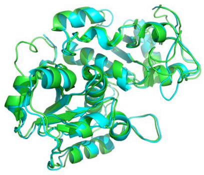	39.6	1.3	52	93.27	✓ fold:c.46.1.2; e-value:1e-80
116	Rv3290c	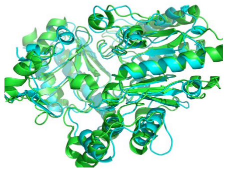	43.8	2.2	29	95.55	✓ fold:c.67.1.4; e-value:e-176
117	Rv3291c	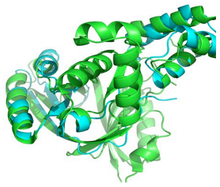	16.3	2.6	27	96.67	✓ fold:a.4.5.32; e-value:6e-24
118	Rv3315c	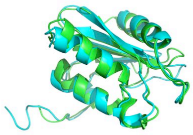	19.3	1.4	40	92.48	✓ fold:c.97.1.1; e-value:9e-59
119	Rv3357	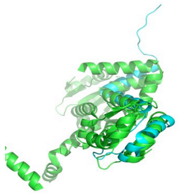	7.7	3.4	45	86.81	✓ fold:d.306.1.1; e-value:1e-27
120	Rv3389c	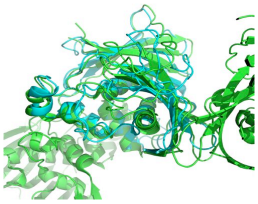	31.5	2.5	39	96.9	✓ fold:d.38.1.4; e-value:5e-60

**121**	**Rv3462c**	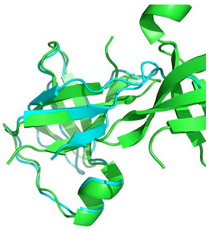	**11.5**	**1.5**	**66**	**94.52**	✓ fold:b.40.4.5; e-value:2e-29
**122**	**Rv3472**	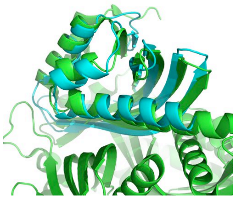	**14.7**	**2.7**	**12**	**74.4**	✓ fold:d.17.4.25; e-value:2e-25
**123**	**Rv3526**	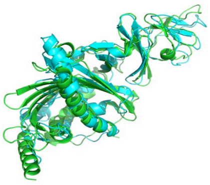	**19.4**	**3.4**	**22**	**81.09**	✓ fold:b.33.1.2; e-value:1e-46
**124**	**Rv3529c**	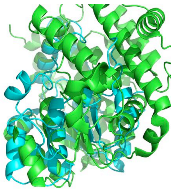	**8.7**	**3.6**	**13**	**56.51**	
**125**	**Rv3545c**	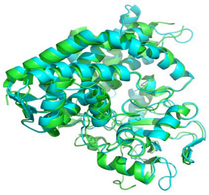	**44**	**2.1**	**29**	**89.38**	✓ fold:a.104.1.1; e-value:0.0
**126**	**Rv3568c**	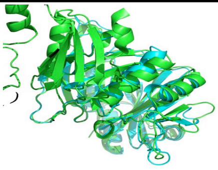	**40.4**	**2**	**39**	**97.67**	✓ fold:d.32.1.3; e-value:1e-78
**127**	**Rv3569c**	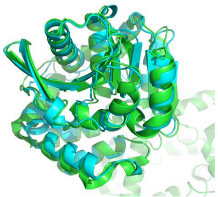	**39.3**	**1.9**	**39**	**97.25**	✓ fold:c.69.1.10; e-value:e-108

128	Rv3570c	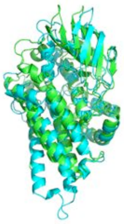	28.9	2.8	18	97.21	✓ fold:e.6.1.1; e-value:6e-88
129	Rv3592	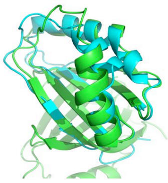	12.6	2	37	91.43	✓ fold:d.58.4.5; e-value:2e-38
130	Rv3676	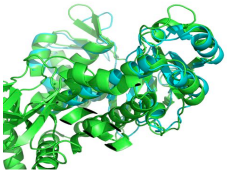	25.9	2.3	26	99.55	✓ fold:b.82.3.2; e-value:2e-58
131	Rv3778c	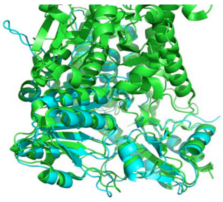	46.1	1.7	26	94.97	✓ fold:c.67.1.3; e-value:e-158
132	Rv3804c	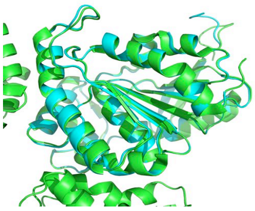	50.5	0.6	82	83.43	✓ fold:c.69.1.3; e-value:e-169
133	Rv3914	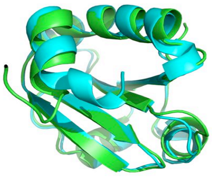	19.6	1.2	57	89.66	✓ fold:c.47.1.1; e-value:7e-51
134	Rv2861c	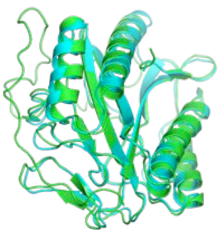	38.4	1.1	51	85.26	✓ fold:d.127.1.1; e-value:1e-70

135	Rv3602c	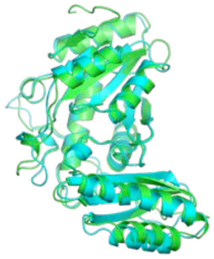	36.8	1.6	42	89.00	
136	Rv3588c	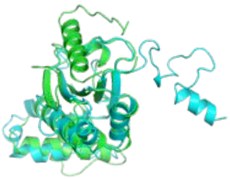	19.5	1.8	29	95.17	✓ fold:c.53.2.1; e-value:6e-60
137	Rv2697c	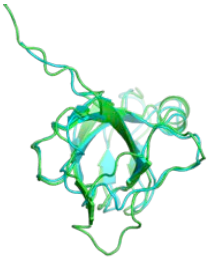	15.1	1.4	31	72.08	✓ fold:b.85.4.1; e-value:2e-57
138	Rv1542c	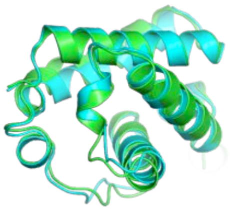	20.3	1	36	84.56	✓ fold:a.1.1.1; e-value:9e-52
139	Rv1092c	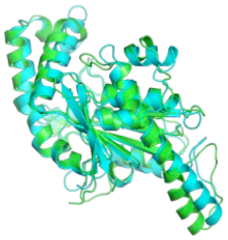	37.6	2.1	53	97.44	✓ fold:c.37.1.6; e-value:e-123
140	Rv2074	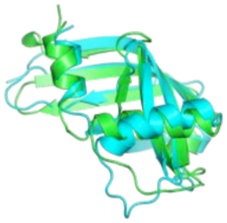	14.4	2.4	13	89.05	✓ fold:b.45.1.1; e-value:2e-21

**141**	**Rv0967**	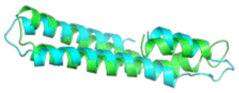	**9.3**	**2.8**	**43**	**66.39**	
**142**	**Rv0644c**	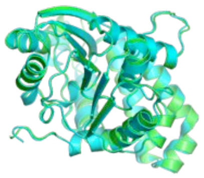	**46.8**	**1.3**	**74**	**99.30**	✓ fold:c.66.1.18; e-value:e-172
**143**	**Rv0674**	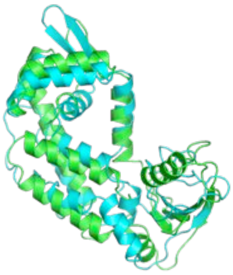	**10.5**	**4.3**	**17**	**93.75**	
**144**	**Rv2220**	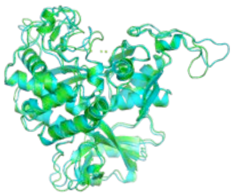	**53.1**	**1.3**	**51**	**99.16**	✓ fold:d.128.1.1; e-value:0.0
**145**	**Rv1264**	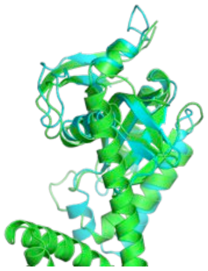	**19.9**	**2.4**	**23**	**41.06**	✓ fold:d.58.29.1; e-value:6e-63
**146**	**Rv3608c**	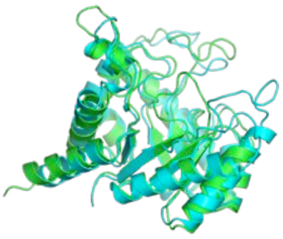	**32.6**	**1.7**	**41**	**96.07**	✓ fold:c.1.21.1; e-value:e-138

**147**	**Rv0432**	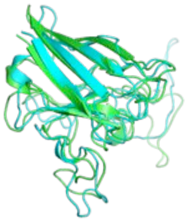	**18.8**	**2.9**	**37**	**70.00**	
**148**	**Rv1411c**	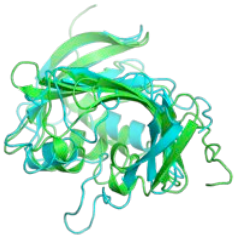	**17.6**	**3**	**33**	**80.93**	
**149**	**Rv1155**	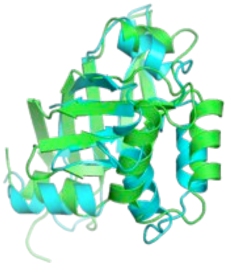	**11.9**	**2.7**	**22**	**90.48**	✓ fold:b.45.1.1; e-value:2e-18
**150**	**Rv2391**	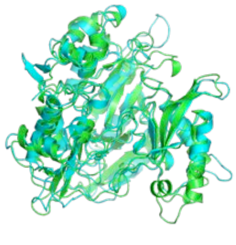	**40.9**	**2.2**	**29**	**94.59**	✓ fold:d.134.1.1; e-value:4e-67
**151**	**Rv0931c**	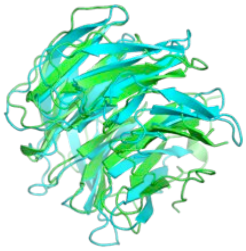	**21.6**	**3.7**	**15**	**35.84**	
**152**	**Rv1389**	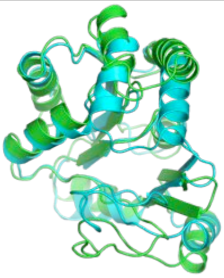	**18.8**	**4.2**	**43**	**86.06**	✓ fold:c.37.1.1; e-value:5e-80

**153**	**Rv2438c**	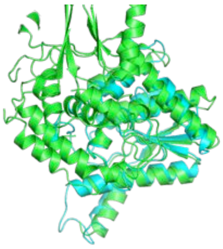	**21.6**	**2.1**	**27**	**32.84**	✓ fold:d.160.1.2; e-value:1e-34
**154**	**Rv3418c**	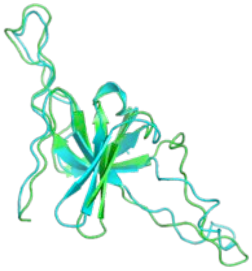	**12**	**3.2**	**60**	**96.00**	✓ fold:b.35.1.1; e-value:1e-23
**155**	**Rv2870c**	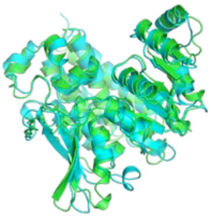	**52.5**	**2.1**	**42**	**91.53**	✓ fold:d.81.1.3; e-value:5e-46
**156**	**Rv2539c**	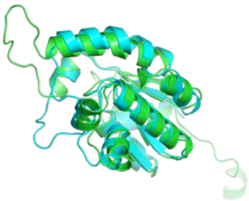	**20.7**	**2**	**37**	**93.75**	✓ fold:c.37.1.2; e-value:7e-18
**157**	**Rv3570c**	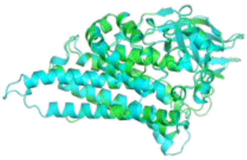	**55.2**	**1.2**	**78**	**98.22**	✓ fold:e.6.1.1; e-value:4e-43
**158**	**Rv2109c**	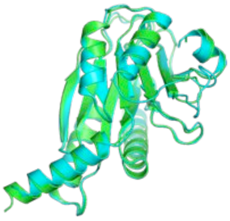	**36.8**	**0.7**	**72**	**90.73**	✓ fold:d.153.1.4; e-value:2e-72

**159**	**Rv2428**	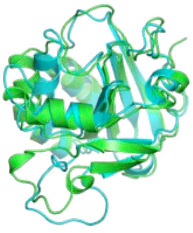	**22.4**	**2.6**	**40**	**84.10**	✔ fold:c.47.1.10; e-value:6e-67
**160**	**Rv2607**	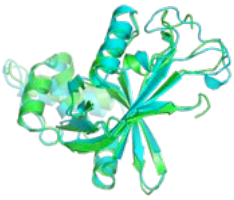	**25**	**1.7**	**40**	**94.20**	✔ fold:b.45.1.1; e-value:1e-94
**161**	**Rv0391**	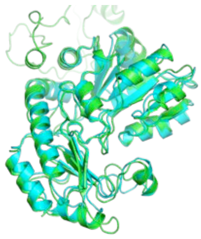	**42.6**	**1.5**	**37**	**81.53**	✔ fold:c.67.1.3; e-value:3e-38
**162**	**Rv2882c**	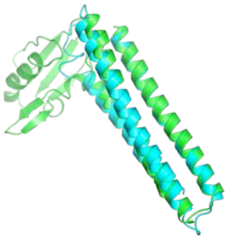	**15**	**2**	**22**	**63.78**	✔ fold:d.67.3.1; e-value:2e-94
**163**	**Rv3607c**	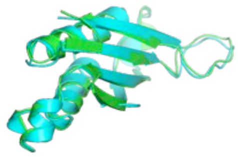	**13.5**	**2.5**	**42**	**87.97**	
**164**	**Rv2878c**	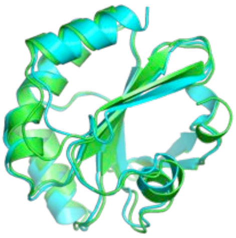	**23.6**	**1.1**	**57**	**75.72**	✔ fold:c.47.1.10; e-value:1e-60

**165**	**Rv2121c**	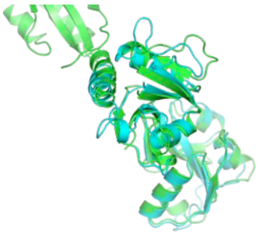	**22.4**	**2.1**	**34**	**72.89**	✓ fold:c.94.1.1; e-value:1e-95
**166**	**Rv1695**	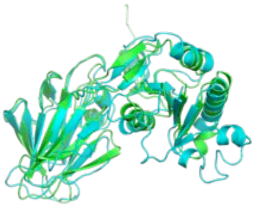	**26.5**	**2.5**	**27**	**95.44**	✓ fold:e.52.1.1; e-value:2e-65
**167**	**Rv2002**	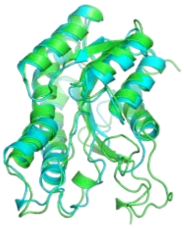	**29.3**	**1.4**	**32**	**77.31**	✓ fold:c.2.1.2; e-value:e-113
**168**	**Rv0938**	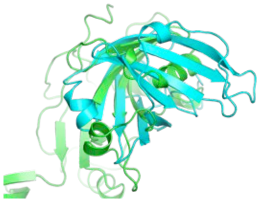	**0.4**	**2.9**	**43**	**17.65**	✓ fold:d.142.2.1; e-value:4e-30
**169**	**Rv3809c**	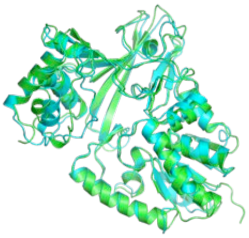	**44.8**	**1.9**	**45**	**94.74**	✓ fold:c.4.1.3; e-value:3e-23
**170**	**Rv2996c**	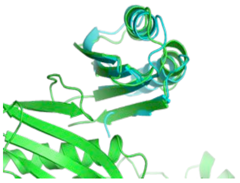	**7.7**	**2.1**	**12**	**13.83**	✓ fold:c.2.1.4; e-value:2e-75

**171**	**Rv0902c**	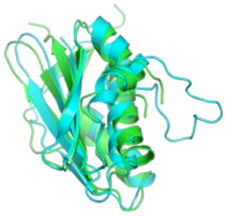	**16.9**	**1.8**	**28**	**31.61**	fold:d.122.1.3; e-value:4e-26
**172**	**Rv2246**	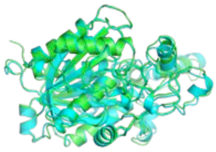	**66.4**	**1.3**	**66**	**98.80**	fold:c.95.1.1; e-value:9e-83
**173**	**Rv1284**	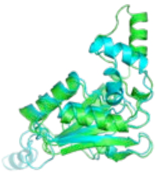	**16.6**	**4.2**	**34**	**97.55**	fold:c.53.2.1; e-value:3e-34
**174**	**Rv2234**	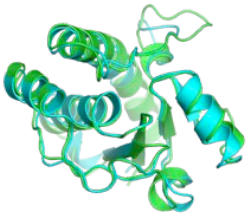	**24.7**	**1.6**	**43**	**90.80**	fold:c.44.1.1; e-value:1e-68
**175**	**Rv2533c**	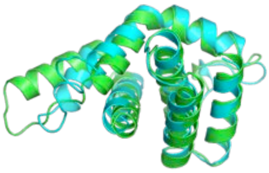	**19**	**2.5**	**37**	**82.69**	fold:a.79.1.1; e-value:4e-25
**176**	**Rv3227**	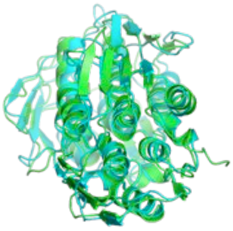	**52.4**	**1.8**	**31**	**91.78**	fold:d.68.2.2; e-value:e-179

177	Rv2122c	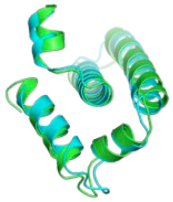	14.1	1.2	25	90.32	
178	Rv2192c	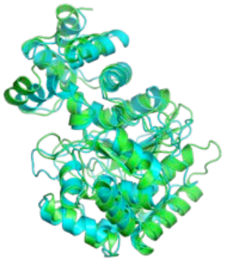	39.5	2	42	91.89	✓ fold:c.27.1.1; e-value:4e-86
179	Rv2540c	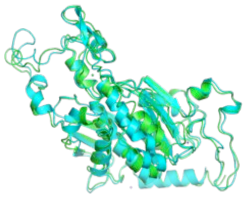	49.4	1.8	45	96.76	✓ fold:d.258.1.1; e-value:e-118
180	Rv0062	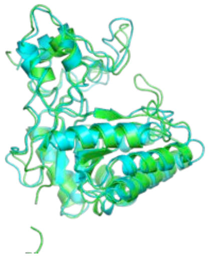	39	1.6	41	74.74	
181	Rv3106	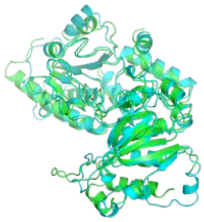	47.8	2	40	98.68	✓ fold:c.3.1.1; e-value:e-107
182	Rv0262c	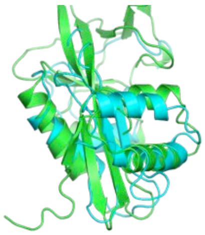	8.5	2.8	17	80.66	✓ fold:d.108.1.1; e-value:9e-23

**183**	**Rv0046c**	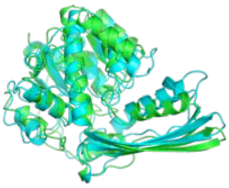	**29.3**	**2.7**	**30**	**94.82**	✓ fold:c.2.1.3; e-value:1e-45
**184**	**Rv0129c**	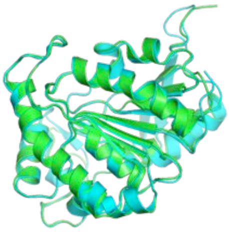	**47.2**	**1.2**	**70**	**84.12**	✓ fold:c.69.1.3; e-value:6e-09
**185**	**Rv1143**	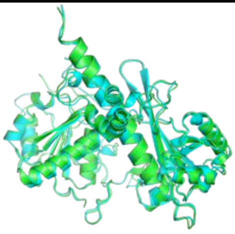	**40.1**	**1.3**	**58**	**95.83**	✓ fold:c.123.1.1; e-value:e-120
**186**	**Rv3214**	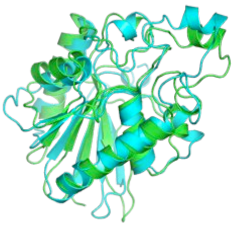	**20.4**	**2.6**	**26**	**97.54**	✓ fold:c.60.1.1; e-value:5e-63
**187**	**Rv0793**	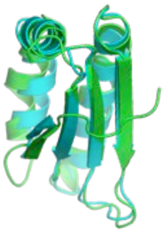	**14.9**	**1.1**	**18**	**89.11**	✓ fold:d.58.4.11; e-value:4e-26
**188**	**Rv0533c**	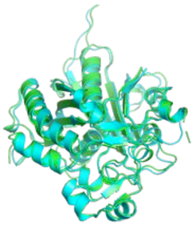	**49.1**	**1.2**	**46**	**97.91**	✓ fold:c.95.1.2; e-value:3e-77

189	Rv1626	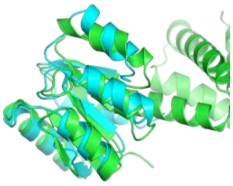	18	1.9	32	57.56	✓ fold:c.23.1.1; e-value:3e-27
190	Rv2763c	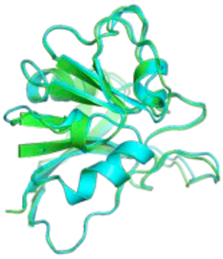	29.4	0.7	72	98.74	✓ fold:c.71.1.1; e-value:3e-34
191	Rv2445c	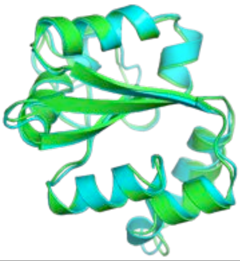	25	0.9	52	97.06	✓ fold:d.58.6.1; e-value:5e-63
192	Rv2537c	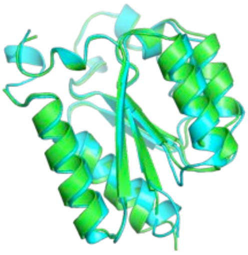	23.8	1.2	41	99.32	✓ fold:c.23.13.1; e-value:5e-81
193	Rv2238c	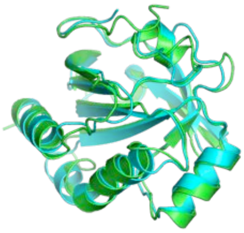	24.1	1.7	30	100.00	✓ fold:c.47.1.10; e-value:1e-44
194	Rv0503c	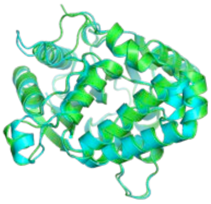	44.6	0.9	57	96.03	✓ fold:c.66.1.18; e-value:8e-15

195	Rv1483	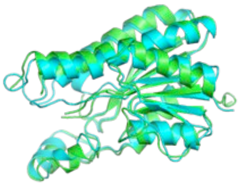	30.9	1.5	34	93.12	✓ fold:c.2.1.2; e-value:2e-60
196	Rv2461c	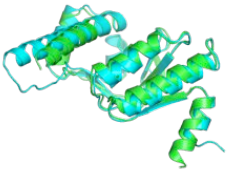	28.1	1.4	55	88.00	
197	Rv0390	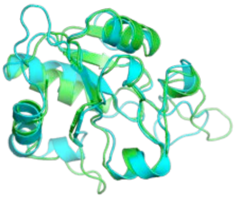	16.4	2	28	99.29	✓ fold:c.46.1.3; e-value:5e-06
198	Rv3361c	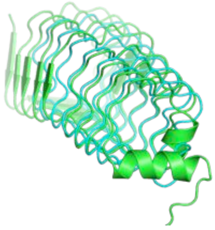	19.3	1.8	23	81.42	✓ fold:b.80.8.1; e-value:8e-61
199	Rv1009	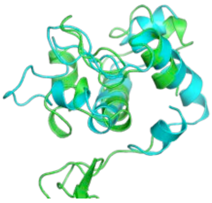	6	3.1	19	25.97	
200	Rv2429	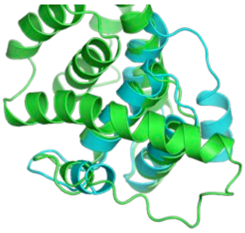	5	2.2	27	42.37	✓ fold:a.152.1.1; e-value:2e-20

**201**	**Rv3048c**	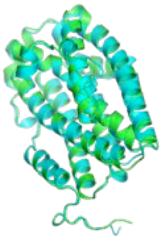	**46.2**	**0.9**	**74**	**87.65**	✔ fold:a.25.1.2; e-value:e-153
**202**	**Rv3875**	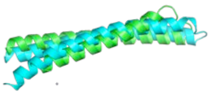	**8**	**2.9**	**17**	**91.58**	
**203**	**Rv1170**	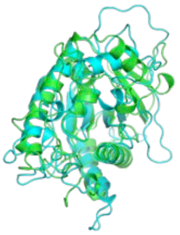	**17.2**	**2.7**	**30**	**94.06**	
**204**	**Rv1372**	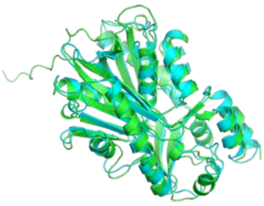	**45.5**	**2.1**	**31**	**90.59**	✔ fold:c.95.1.2; e-value:5e-89
**205**	**Rv1596**	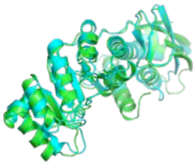	**30.4**	**1.6**	**33**	**96.14**	✔ fold:c.1.17.1; e-value:3e-58
**206**	**Rv2717c**	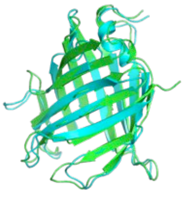	**22.2**	**1.5**	**32**	**96.95**	✔ fold:b.60.1.8; e-value:8e-76

**207**	**Rv1416**	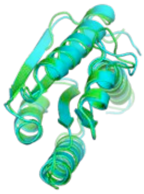	**26.4**	**1**	**47**	**91.25**	✓ fold:c.16.1.1; e-value:9e-59
**208**	**Rv0983**	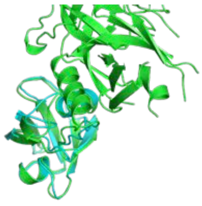	**13.7**	**1.4**	**18**	**19.83**	
**209**	**Rv3247c**	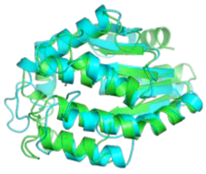	**21.1**	**2.3**	**26**	**96.73**	✓ fold:c.37.1.1; e-value:1e-50
**210**	**Rv0462**	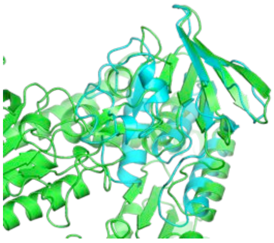	**5.7**	**2.8**	**20**	**34.48**	✓ fold:c.3.1.5; e-value:6e-62
**211**	**Rv0470c**	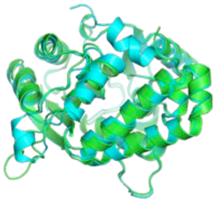	**41.9**	**1**	**73**	**98.61**	✓ fold:c.66.1.18; e-value:e-172
**212**	**Rv1837c**	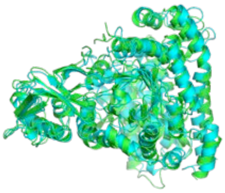	**31.4**	**2.9**	**58**	**97.71**	✓ fold:c.1.13.1; e-value:0.0

**213**	**Rv2110c**	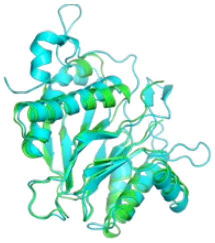	**29**	**1.6**	**67**	**87.63**	✓ fold:d.153.1.4; e-value:e-128
**214**	**Rv0137c**	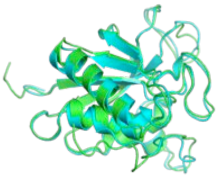	**26.1**	**1.7**	**43**	**90.11**	✓ fold:d.58.28.1; e-value:1e-76
**215**	**Rv0058**	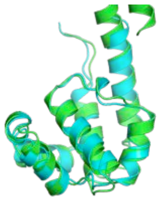	**20.8**	**1.1**	**40**	**12.81**	
**216**	**Rv1314c**	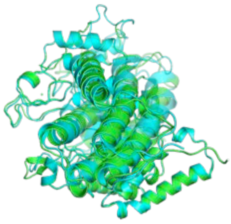	**21.6**	**1.8**	**37**	**79.27**	✓ fold:a.25.2.2; e-value:3e-34
**217**	**Rv1636**	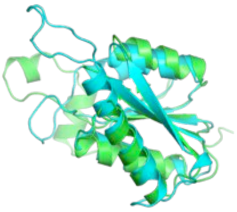	**12.6**	**1.5**	**25**	**95.89**	
**218**	**Rv1885c**	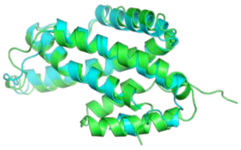	**15.2**	**2.4**	**23**	**64.82**	✓ fold:a.130.1.4; e-value:9e-33

**219**	**Rv3710**	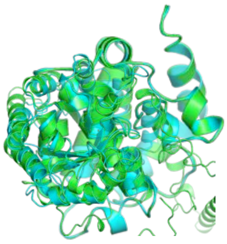	**33.5**	**2.1**	**25**	**44.88**	✓ fold:c.1.10.5; e-value:3e-71
**220**	**Rv3280**	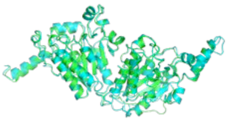	**55.6**	**0.9**	**69**	**96.35**	✓ fold:c.14.1.4; e-value:e-150
**221**	**Rv1848**	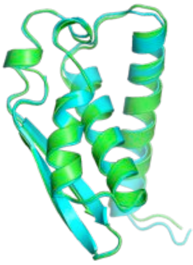	**19.1**	**0.9**	**65**	**99.00**	✓ fold:d.8.1.1; e-value:3e-34
**222**	**Rv2965c**	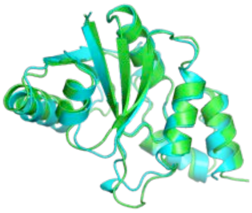	**25**	**1.6**	**42**	**96.89**	✓ fold:c.26.1.3; e-value:6e-62
**223**	**Rv1710**	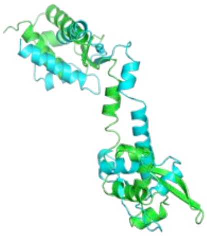	**6**	**3.5**	**31**	**68.40**	✓ fold: a.4.5.60; e-value: 5e-32
**224**	**Rv1098c**	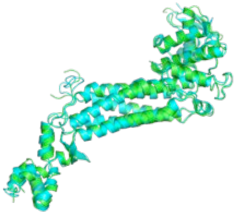	**53**	**1.6**	**51**	**95.99**	✓ fold:a.127.1.1; e-value:4e-62

**225**	**Rv2754c**	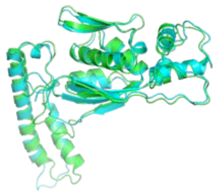	**28.4**	**2.2**	**64**	**97.20**	✓ fold:d.207.1.1; e-value:7e-45
**226**	**Rv2626c**	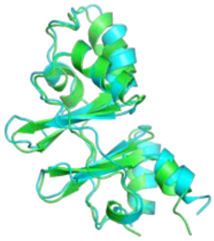	**17.2**	**2.1**	**19**	**86.01**	✓ fold:d.37.1.1; e-value:2e-12
**227**	**Rv1654**	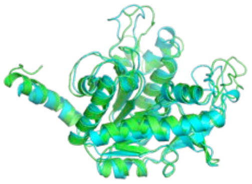	**38.4**	**2**	**45**	**95.58**	✓ fold:c.73.1.2; e-value:e-142
**228**	**Rv1677**	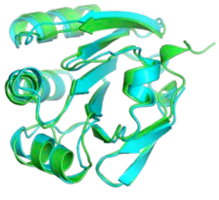	**23.2**	**1.2**	**56**	**69.78**	
**229**	**Rv1625c**	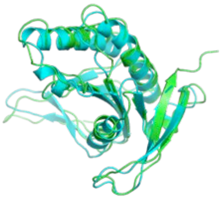	**18.4**	**2.5**	**24**	**39.50**	
**230**	**Rv2883c**	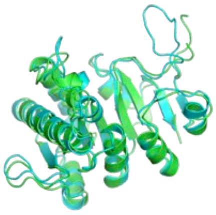	**34.6**	**1.6**	**46**	**88.51**	✓ fold:c.73.1.3; e-value:2e-48

231	Rv1886c	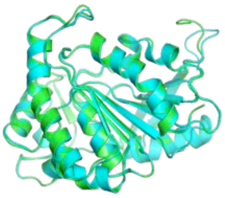	50.7	0.8	82	87.69	✓ fold:c.69.1.3; e-value:3e-20
232	Rv0736	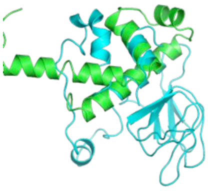	1.5	2.6	26	64.00	
233	Rv2841c	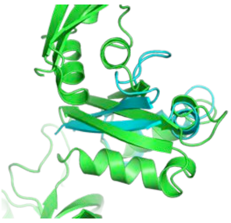	1.5	3.3	24	15.85	✓ fold:d.202.1.1; e-value:2e-34
234	Rv3803c	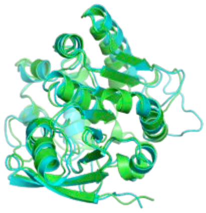	37.4	1.7	42	87.63	✓ fold:c.69.1.3; e-value:5e-14
235	Rv0934	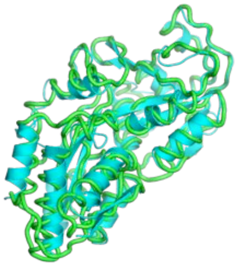	41	2	32	85.83	
236	Rv3853	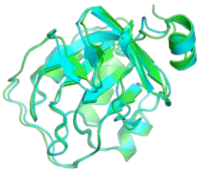	28.1	1	45	95.54	✓ fold:c.8.7.1; e-value:3e-36

237	Rv2793c	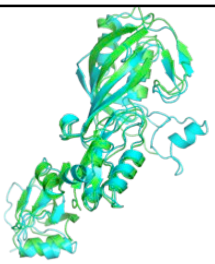	24.2	2.1	34	95.97	✓ fold:d.265.1.2; e-value:1e-80
238	Rv1484	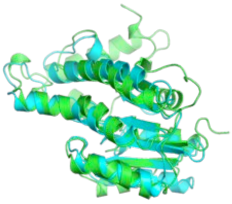	20	2.3	15	72.12	✓ fold:c.2.1.2; e-value:e-123
239	Rv2245	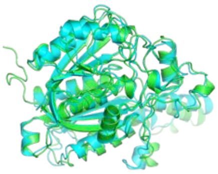	59.5	2.2	40	96.88	fold:c.95.1.1; e-value:e-129
240	Rv3307	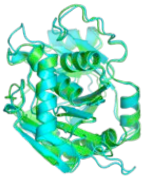	37.6	1.7	58	99.63	✓ fold:c.56.2.1; e-value:e-146
241	Rv0491	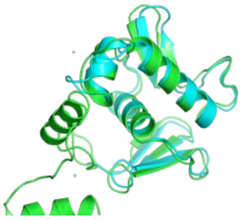	16.5	1.5	45	44.05	✓ fold:c.23.1.1; e-value:8e-48
242	Rv2465c	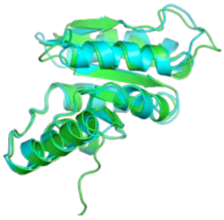	24.4	1.5	36	91.98	✓ fold:c.121.1.1; e-value:1e-32

**243**	**Rv0760c**	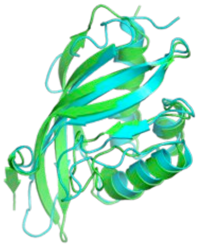	**17**	**2.2**	**24**	**96.40**	✔ fold:d.17.4.3; e-value:2e-10
**244**	**Rv2945c**	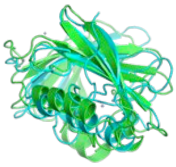	**17.6**	**2.8**	**31**	**88.41**	✔ fold:b.125.1.3; e-value:2e-33
**245**	**Rv2925c**	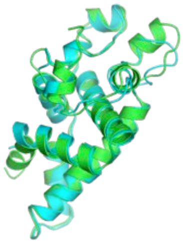	**23.3**	**1.2**	**44**	**60.83**	✔ fold:a.149.1.1; e-value:3e-29
**246**	**Rv1900c**	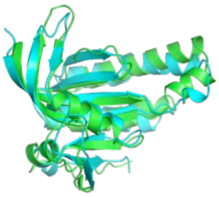	**23.5**	**2.2**	**29**	**36.15**	
**247**	**Rv2217**	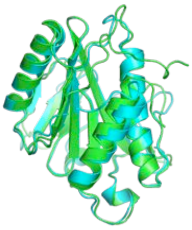	**29.4**	**1.7**	**43**	**88.70**	
**248**	**Rv3356c**	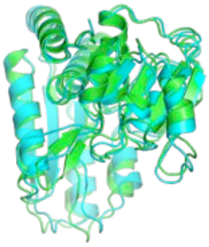	**39.1**	**1.7**	**43**	**99.64**	✔ fold:c.2.1.7; e-value:5e-58

249	Rv2543	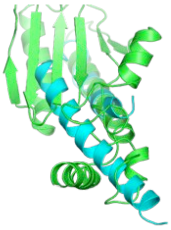	0.2	4	25	25.11	
250	Rv2118c	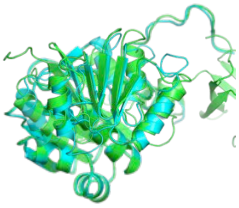	13.5	2.5	14	51.79	✓ fold:c.66.1.13; e-value:6e-09
251	Rv2711	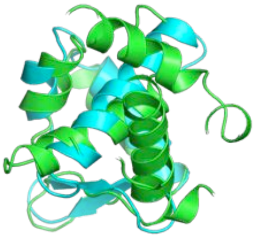	7.6	2.7	23	30.00	✓ fold:a.76.1.1; e-value:9e-32
252	Rv0548c	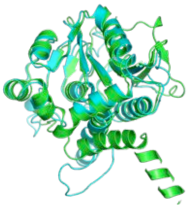	25.2	1.7	34	74.20	✓ fold:c.14.1.3; e-value:4e-98
253	Rv2470	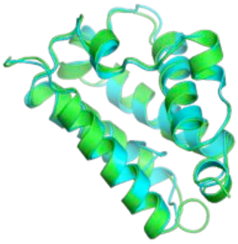	21.7	0.8	59	96.88	✓ fold:a.1.1.1; e-value:3e-39
254	Rv3710	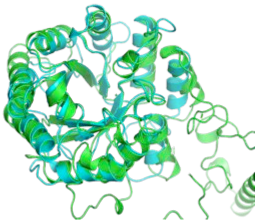	33.2	2.1	25	44.88	✓ fold:c.1.10.5; e-value:3e-71

**255**	**Rv2995c**	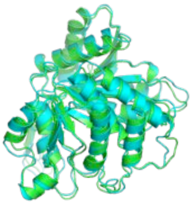	**45.4**	**1.9**	**41**	**99.70**	✓ fold:c.77.1.1; e-value:e-147
**256**	**Rv2150c**	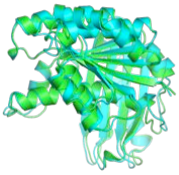	**48.1**	**1.2**	**66**	**80.47**	✓ fold:c.32.1.1; e-value:8e-23
**257**	**Rv1267c**	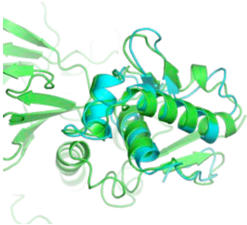	**8.2**	**2.2**	**16**	**22.68**	
**258**	**Rv0903c**	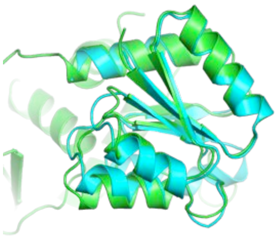	**19.3**	**1.6**	**29**	**51.07**	✓ fold:c.23.1.1; e-value:6e-63
**259**	**Rv3303c**	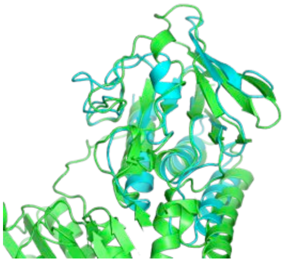	**18.6**	**2.2**	**23**	**32.25**	✓ fold:c.3.1.5; e-value:2e-39
**260**	**Rv0733**	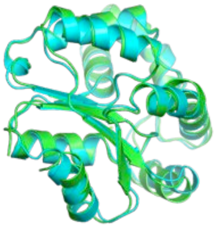	**29.6**	**1.2**	**45**	**100.00**	✓ fold:c.37.1.1; e-value:3e-80

**261**	**Rv2225**	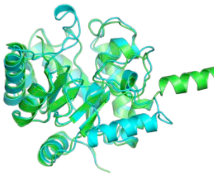	**33.9**	**1.8**	**48**	**93.59**	
**262**	**Rv2498c**	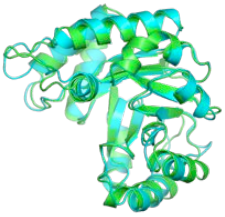	**29.6**	**1.6**	**32**	**77.66**	
**263**	**Rv2773c**	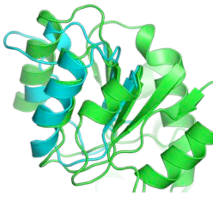	**2.7**	**2.5**	**21**	**24.90**	✓ fold:d.81.1.3; e-value:4e-34
**264**	**Rv0440**	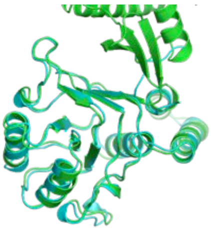	**30.5**	**1.3**	**64**	**35.93**	✓ fold:i.16.1.1; e-value:0.0
**265**	**Rv2991**	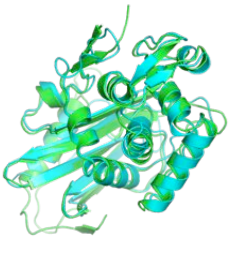	**14.5**	**2**	**24**	**80.98**	✓ fold:b.45.1.1; e-value:4e-27
**266**	**Rv3628**	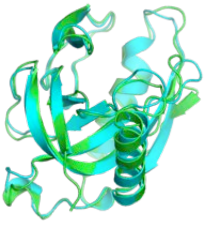	**26.7**	**1.3**	**47**	**96.30**	✓ fold:b.40.5.1; e-value:2e-73

267	Rv1700	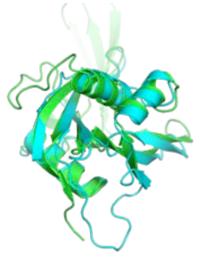	16	1.7	29	66.18	fold:d.113.1.1; e-value:9e-40
268	Rv3855	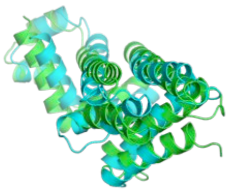	14.1	3.5	17	83.80	fold:a.121.1.1; e-value:3e-47
269	Rv2911	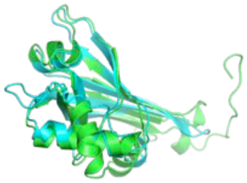	35.6	1.5	32	83.85	
270	Rv0467	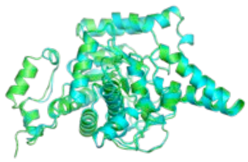	60.1	0.6	67	95.56	fold:c.1.12.7; e-value:0.0
271	Rv3392c	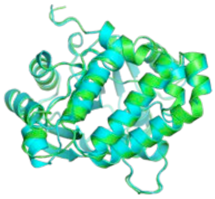	46.7	1.3	74	99.30	fold:c.66.1.18; e-value:e-166
272	Rv0009	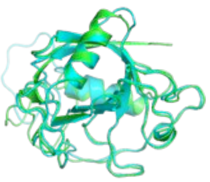	27.6	1.4	51	91.21	fold:b.62.1.1; e-value:5e-63

273	Rv0642c	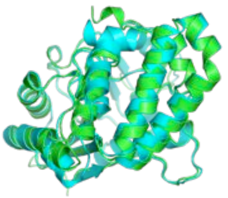	40.1	1.7	57	92.69	✓ fold:c.66.1.18; e-value:2e-18
274	Rv1293	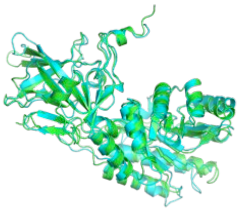	45.5	1.9	31	96.64	✓ fold:c.1.6.1; e-value:4e-44
275	Rv3240c	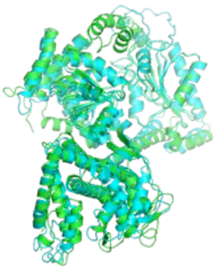	44.7	2.9	51	88.20	✓ fold:c.37.1.19; e-value:e-112
276	Rv0216	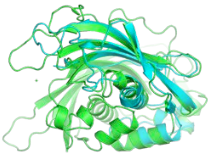	10.4	2.7	17	44.21	
277	Rv3846	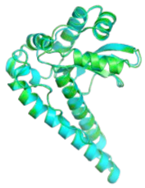	32	0.8	64	94.69	✓ fold:d.44.1.1; e-value:1e-45
278	Rv2276	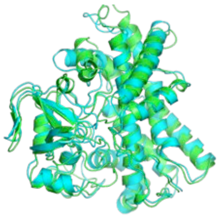	42.9	2.2	25	95.45	✓ fold:a.104.1.1; e-value:e-104

279	Rv0819	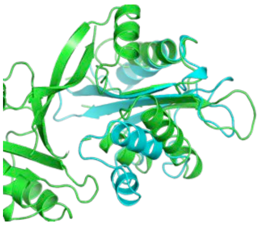	6.3	2.5	16	36.83	✓ fold:d.108.1.1; e-value:6e-34
280	Rv1347c	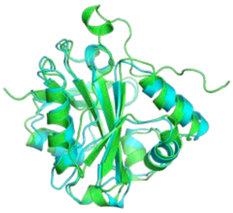	23.2	1.6	25	81.43	✓ fold:d.108.1.1; e-value:5e-16
281	Rv1908c	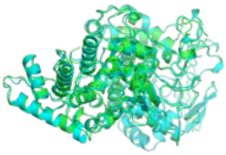	67.7	1	68	95.68	✓ fold:a.93.1.3; e-value:0.0
282	Rv3465	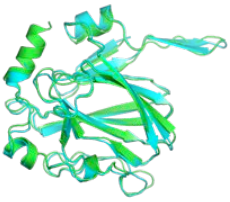	31.4	1.2	49	95.54	✓ fold:b.82.1.1; e-value:e-108
283	Rv1932	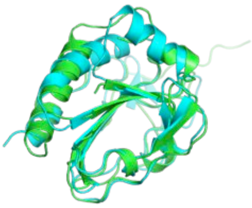	22	1.8	52	98.18	✓ fold:c.47.1.10; e-value:8e-44
284	Rv3913	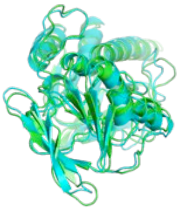	38.4	1.7	38	90.45	✓ fold:c.3.1.5; e-value:6e-16

**285**	**Rv3607c**	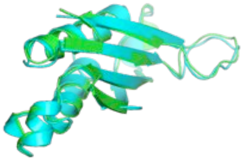	**20.1**	**1**	**42**	**87.97**	
**286**	**Rv3423c**	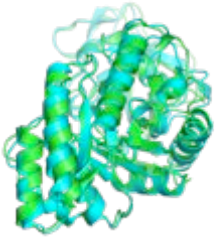	**46.2**	**1.5**	**48**	**94.56**	✓ fold:c.1.6.1; e-value:1e-83
**287**	**Rv2714**	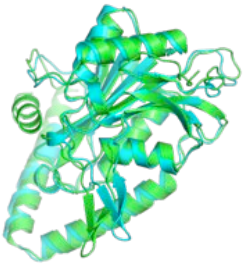	**34.3**	**2**	**46**	**81.79**	✓ fold:c.56.8.1; e-value:3e-87
**288**	**Rv1379**	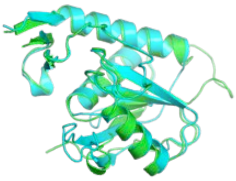	**24.2**	**1.5**	**53**	**93.26**	✓ fold:c.61.1.1; e-value:1e-17
**289**	**Rv1094**	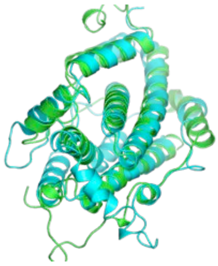	**20.7**	**2.3**	**23**	**92.00**	✓ fold:a.25.1.2; e-value:1e-84
**290**	**Rv0489**	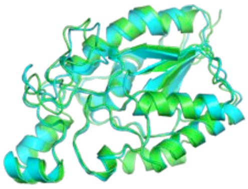	**33.7**	**1.4**	**51**	**89.56**	✓ fold:c.60.1.1; e-value:e-126
