# Supplementary material for: Structural Annotation of Mycobacterium tuberculosis Proteome
Source: PLoS One. 2011 Oct 31;6(10):e27044. doi: 10.1371/journal.pone.0027044 (PMC3205055; doi:10.1371/journal.pone.0027044)
Supplement: Table S3 — The various criteria and cut-offs used in the pipeline have been mentioned along with the statistics. (DOC) [file pone.0027044.s013.doc]

| **Sl. No** | **METHODOLOGY** | **STATISTICS** |
| --- | --- | --- |
| 1. | **SELECTION OF MODELS**  Selection of one protein models from MODBASE per gene using the score obtained from “Sequence coverage x Sequence Identity”  +  PDB structures from RCSB PDB  +  54 additional models using MODPIPE, after identifying nearest homologue through PSI-BLAST having a e-value cut-off of 0.0001 and coverage of 70 residues. | 2511 proteins  +  312 proteins  +  54 proteins  Total = 2877 proteins |
| 2. | **QUALITY OF PROTEIN MODELS**  Normalized DOPE score < 0  ERRAT Score > 60  ProQ with LGscore > 1.5 and MaxSub > 0.1  Ramachandran Plot > 90% in allowed region  Protein Models with atleast one of the above criteria satisfied. | 2555 proteins  1775 proteins  2398 proteins  2846 proteins  2877 (include crystal structures as such) |
| 3. | **FOLD BASED FUNCTION ANNOTATION**  Fold was determined for each of the protein models and corresponding superfamily based function annotation was determined (Christian Vogels data) | 2364 proteins |
| 4. | **BINDING SITE DETECTION AND LIGAND ASSOCIATION**  Every protein was subjected to Binding Site Detection through consensus of PocketDepth (success rate of 82%) and LigSite CSC (success rate of 75%).  Ligand Associations for predicted pockets were obtained through PocketMatch by comparing it against MOAD database. Only those having score of more than 0.6 was chosen. (Score of 0.4 is significant)  ProFunc  (Has its own E-value associated with the hits which is displayed in the database)  Ligand Binding Templates  Enzyme Templates  DNA Binding Templates | 1728 Ligand Associations  1842 Hits  647 Hits  741 Hits |
