# Supplementary material for: Structural Annotation of Mycobacterium tuberculosis Proteome
Source: PLoS One. 2011 Oct 31;6(10):e27044. doi: 10.1371/journal.pone.0027044 (PMC3205055; doi:10.1371/journal.pone.0027044)
Supplement: Table S6 — Of the 312 crystal structures taken as the test set for validation, 105 of them are complexes with biologically relevant ligands, forming 179 different binding sites. 154 of these sites in 97 of the proteins were predicted correctly by PocketDepth (as shown in Column 2) and 46 ligand associations that could be predicted using PocketMatch alone (above the threshold used) were all found to be correct. There were of course many other ligand associations that appear correct, but fall below the stringent thresholds chosen in this study and hence have not been counted. (PDF) [file pone.0027044.s016.pdf]

Sl. No	Figure	Protein	PDBID	Chain	Pocket ID	Lig ID	PMIN Score	PMAX Score
1	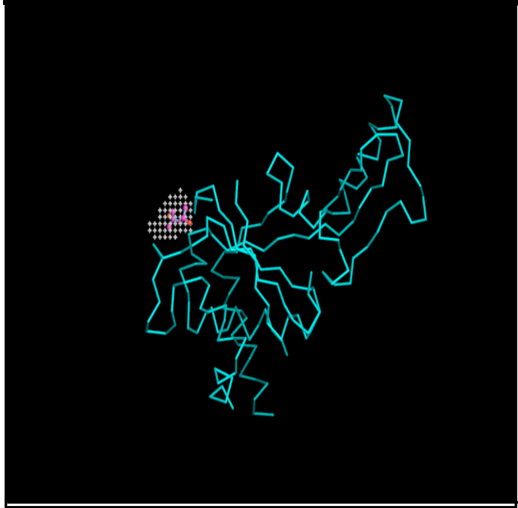	Rv0005	3m4i	A	r1	MPD	1	0.387
2	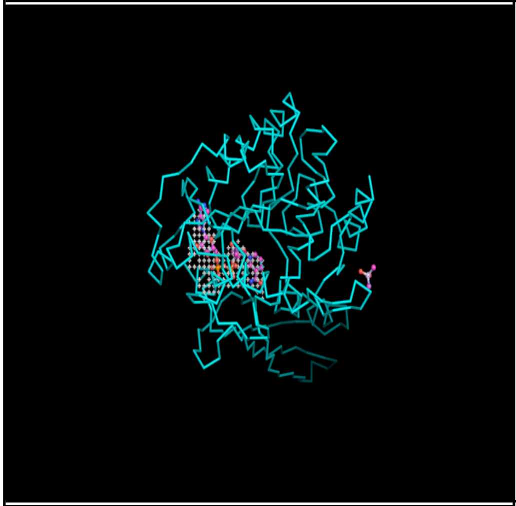	Rv0046c	1gr0	A	r1	NAD	0.967	0.517
3	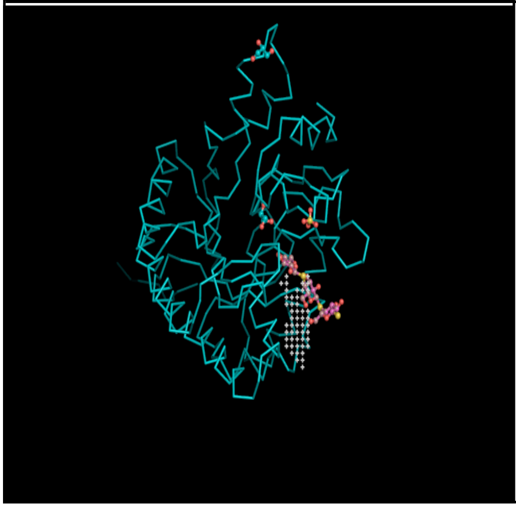	Rv0062	1uoz	A	r17	GLC	0.507	0.255

4	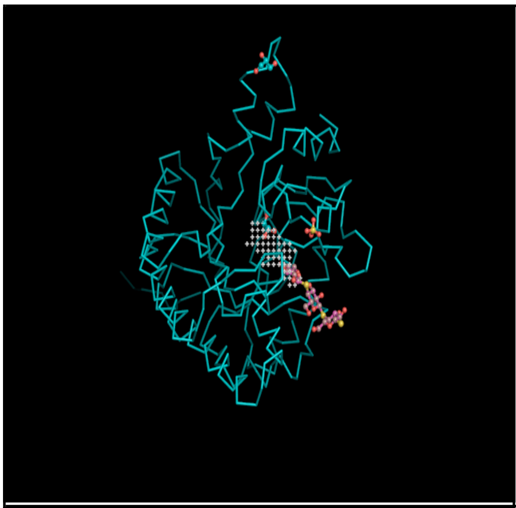	Rv0062	1uoz	A	r3	GLC	0.903	0.164
5	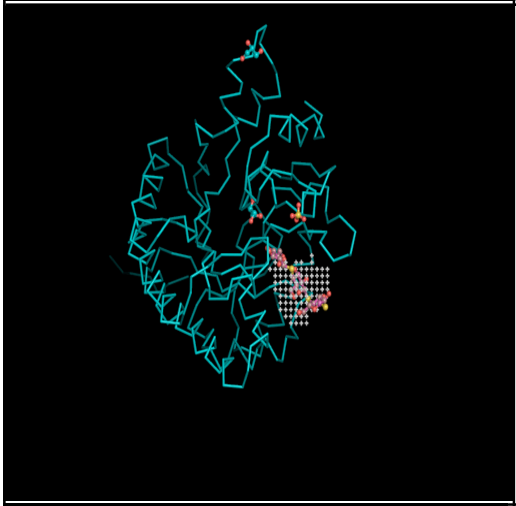	Rv0062	1uoz	A	r5	GLC	0.823	0.261
6	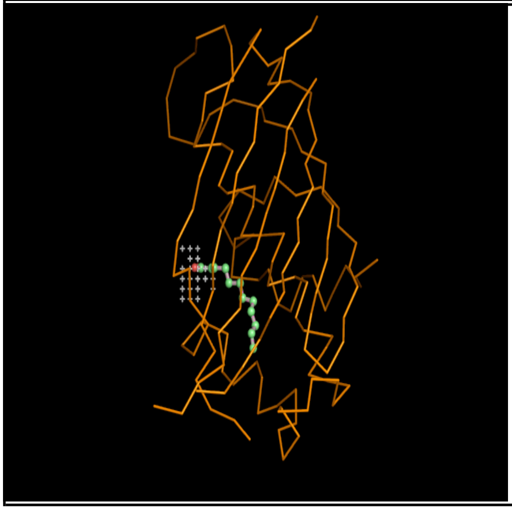	Rv0098	2pfc	A	r5	PLM	0.64	0.429

7	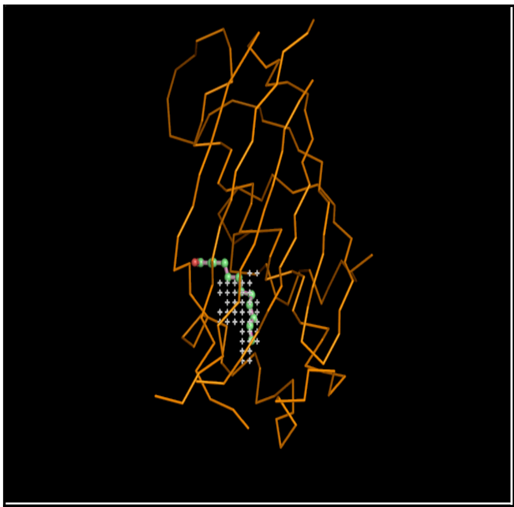	Rv0098	2pfc	A	r7	PLM	0.955	0.262
8	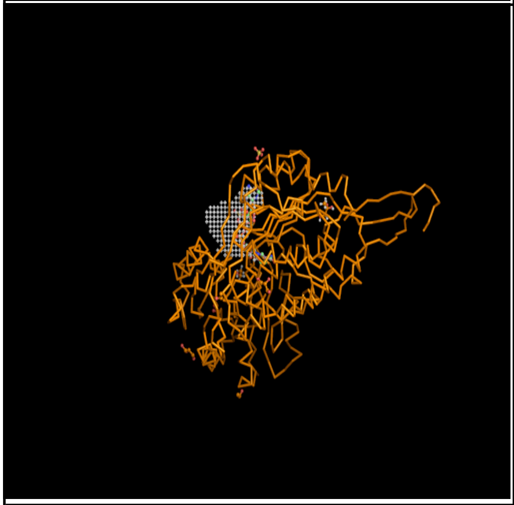	Rv0223c	3b4w	A	r2	NAD	0.973	0.352
9	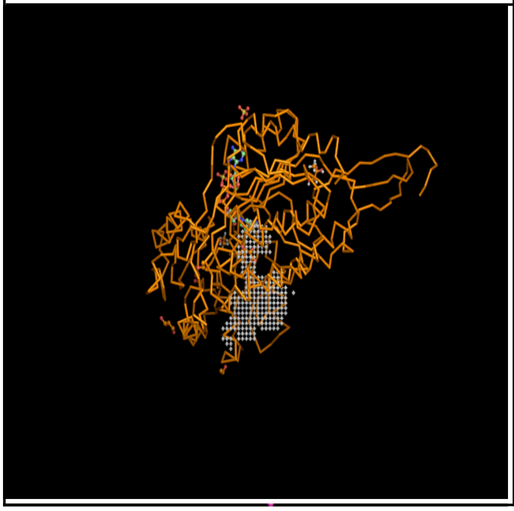	Rv0223c	3b4w	A	r3	NAD	0.857	0.27

10	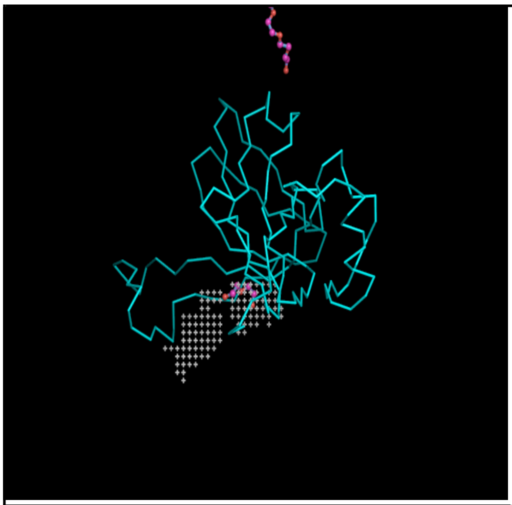	Rv0321	2qlp	A	r4	TTP	0.404	0.265
11	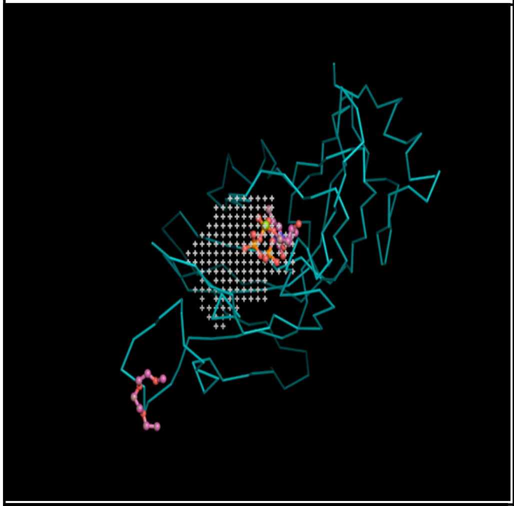	Rv0321	2qxx	A	r2	TTP	1	0.173
12	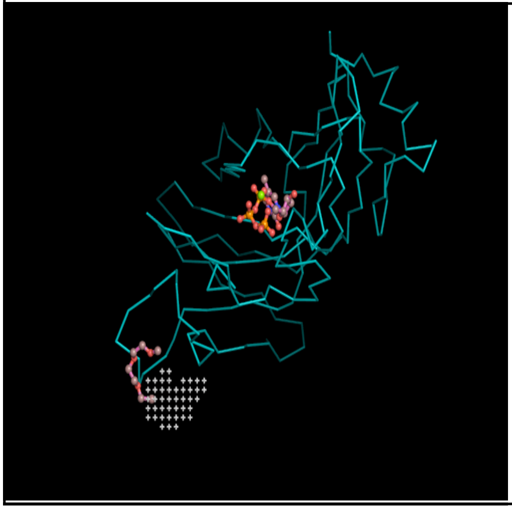	Rv0321	2qxx	A	r4	TTP	0.375	0.069

13	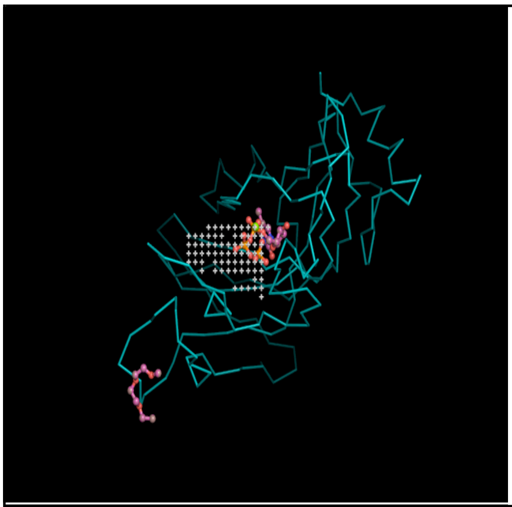	Rv0321	2qxx	A	r8	TTP	0.468	0.205
14	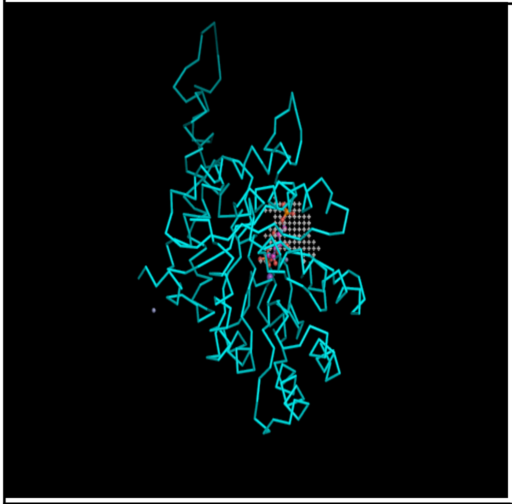	Rv0363c	3elf	A	r4	2FP	0.802	0.48
15	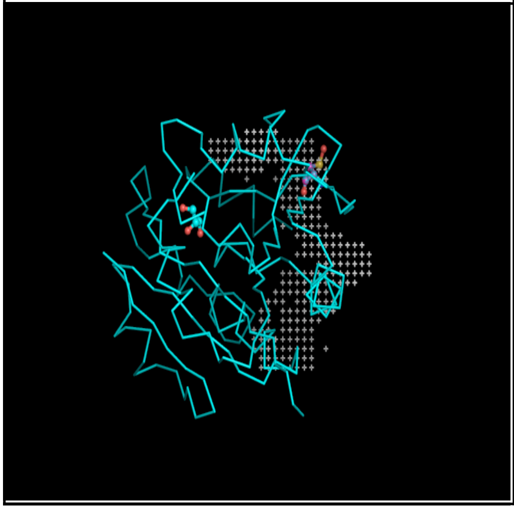	Rv0371c	2wee	A	r10	CSO	1	0.011

16	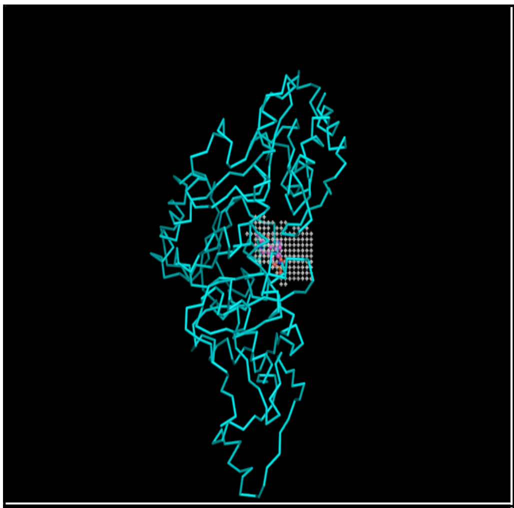	Rv0391	3ndn	A	r3	LLP	0.898	0.288
17	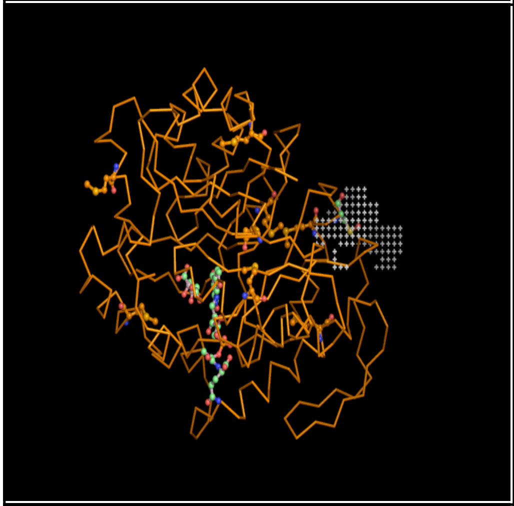	Rv0407	3b4y	A	r17	F42	0.456	0.398
18	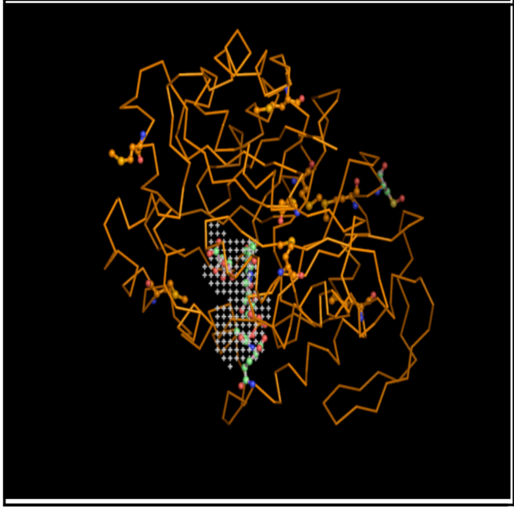	Rv0407	3b4y	A	r4	F42	0.904	0.391

19	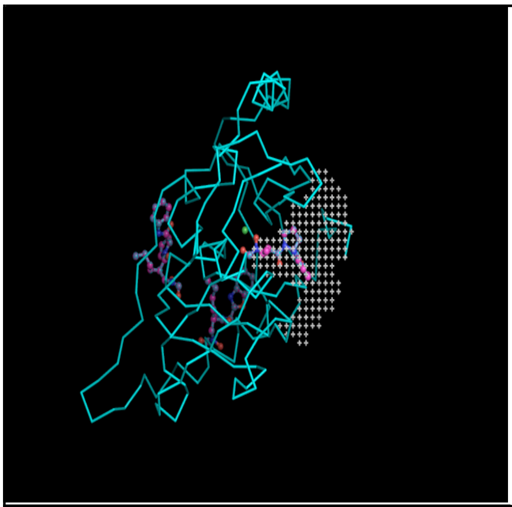	Rv0429c	3e3u	A	r2	NVC	0.685	0.435
20	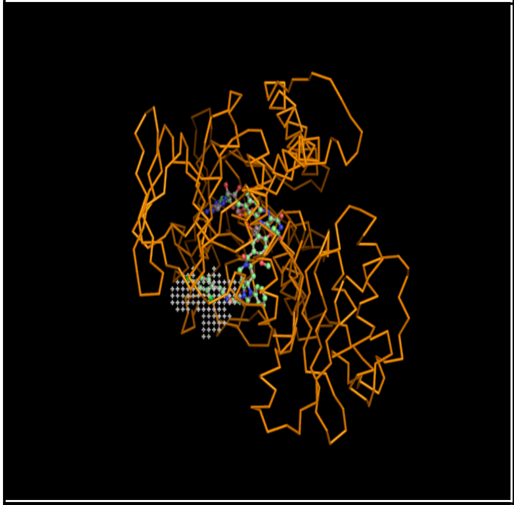	Rv0462	3ii4	A	r3	3II	0.721	0.368
21	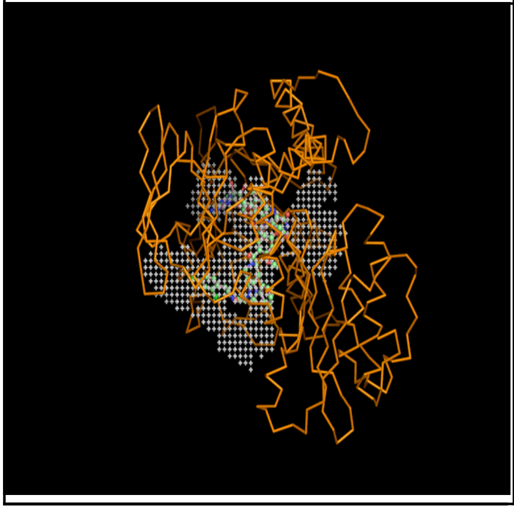	Rv0462	3ii4	A	r4	FAD	1	0.086

22	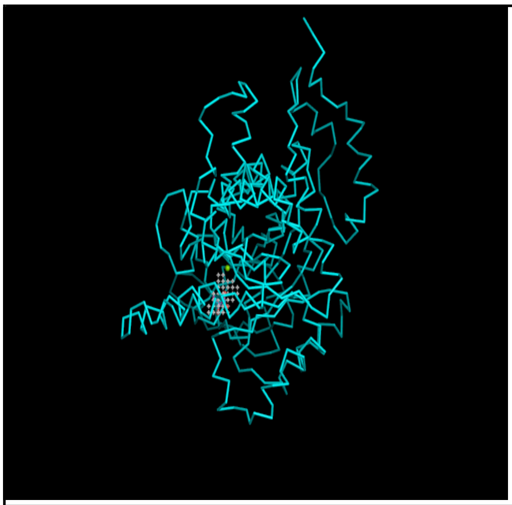	Rv0467	1f8m	A	r3	PYR	1	0.193
23	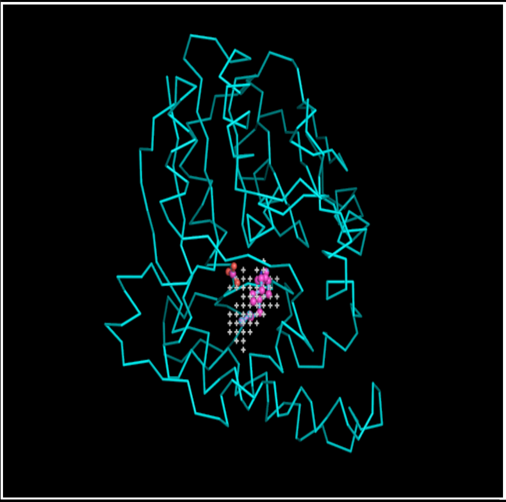	Rv0503c	3hem	A	r2	D22	1	0.24
24	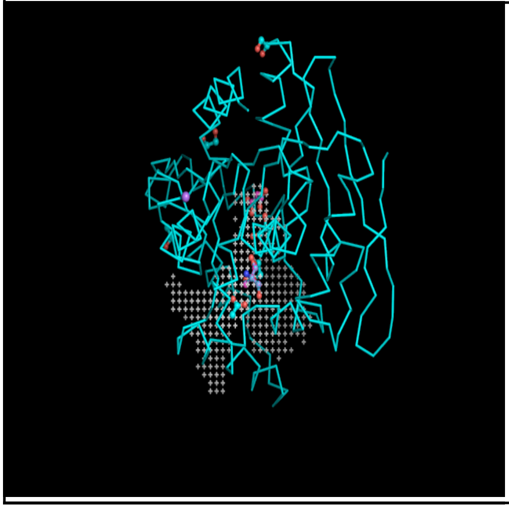	Rv0554	3hss	A	r1	TRS	1	0.02

25	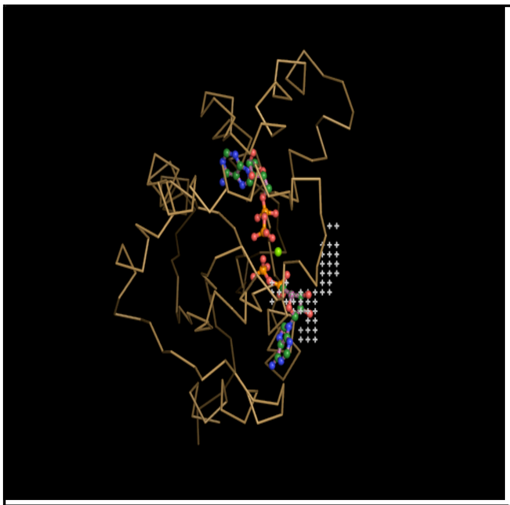	Rv0733	2cdn	A	r13	ADP	0.913	0.112
26	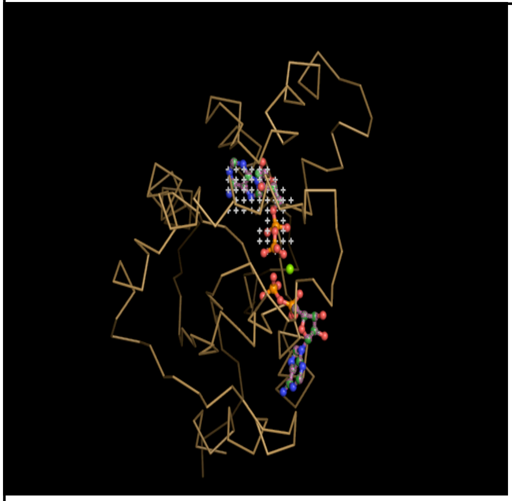	Rv0733	2cdn	A	r2	ADP	0.693	0.468
27	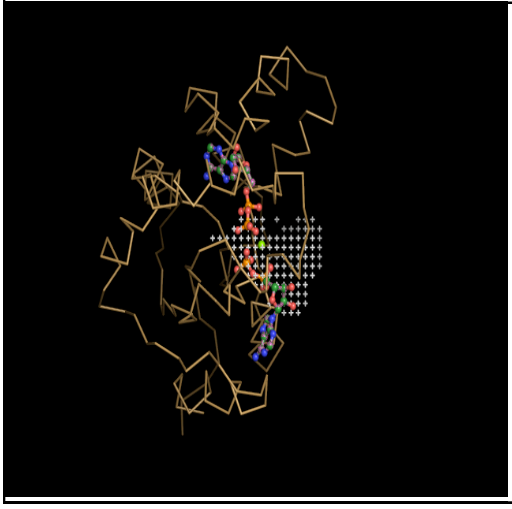	Rv0733	2cdn	A	r3	ADP	0.653	0.414

28	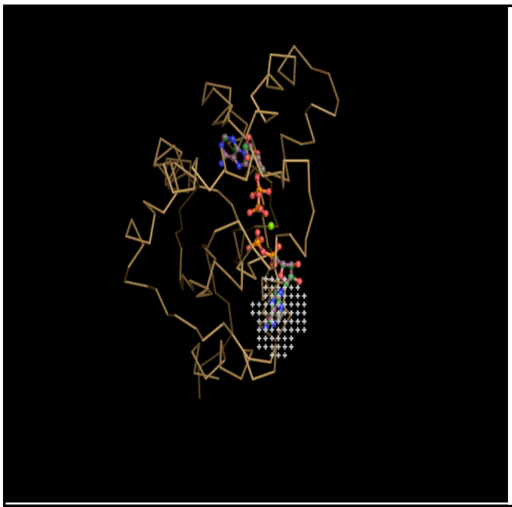	Rv0733	2cdn	A	r4	ADP	0.824	0.141
29	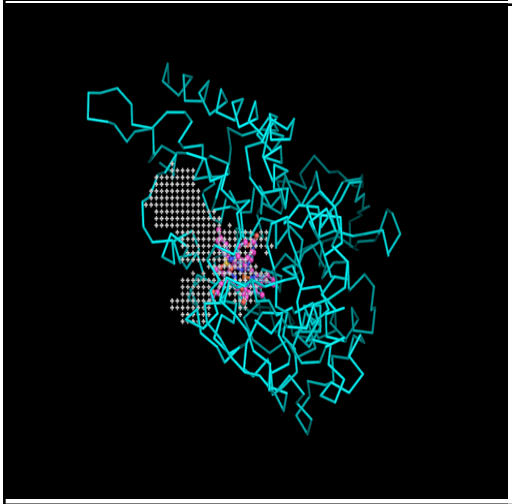	Rv0764c	2w0b	A	r2	HEM	0.997	0.166
30	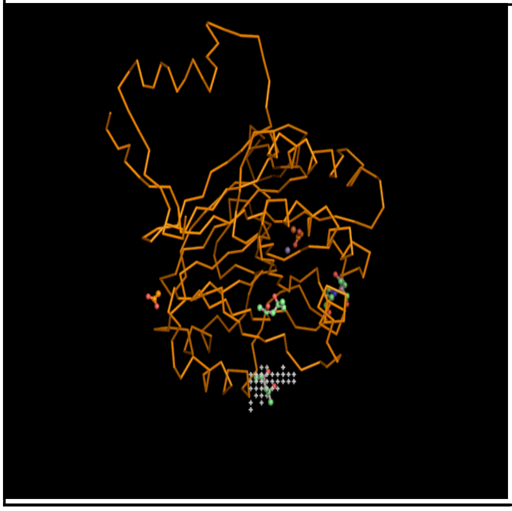	Rv0805	3ib7	A	r17	MPD	0.48	0.29

31	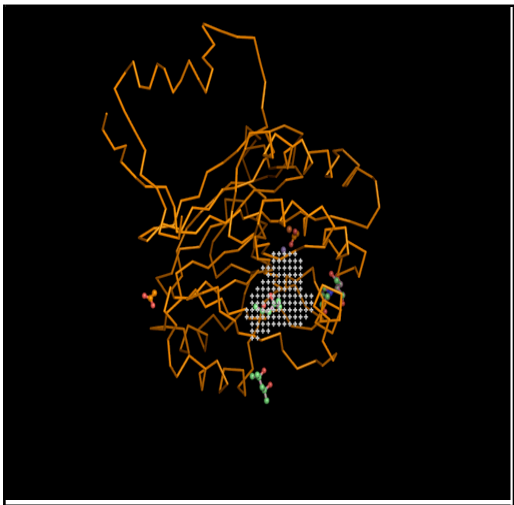	Rv0805	3ib7	A	r3	MPD	0.556	0.216
32	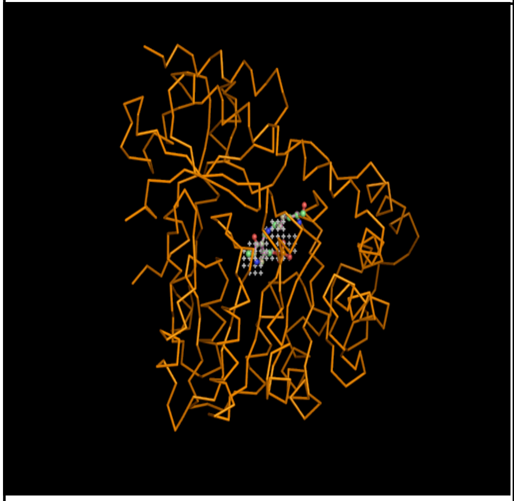	Rv0858c	2o0r	A	r21	LLP	0.692	0.665
33	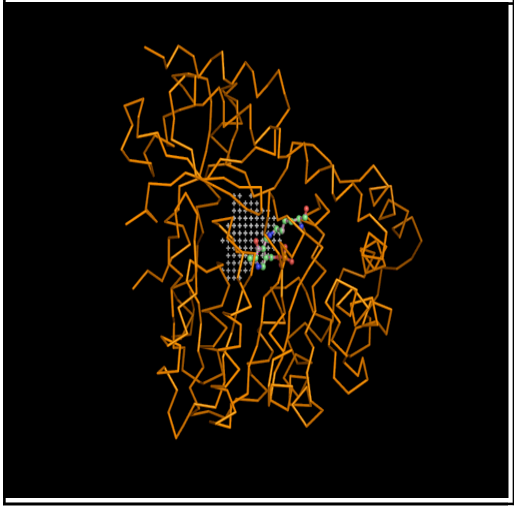	Rv0858c	2o0r	A	r4	LLP	0.474	0.456

34	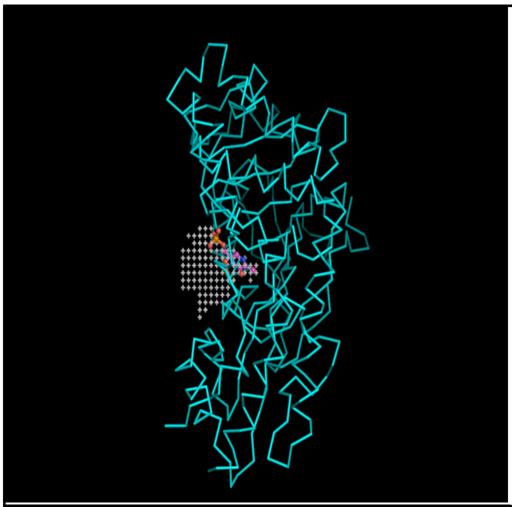	Rv0884c	2fyf	A	r11	PLP	0.957	0.235
35	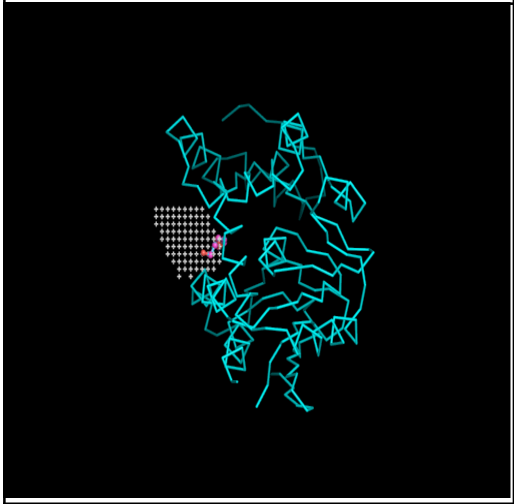	Rv0905	3he2	A	r1	PGE	1	0.201
36	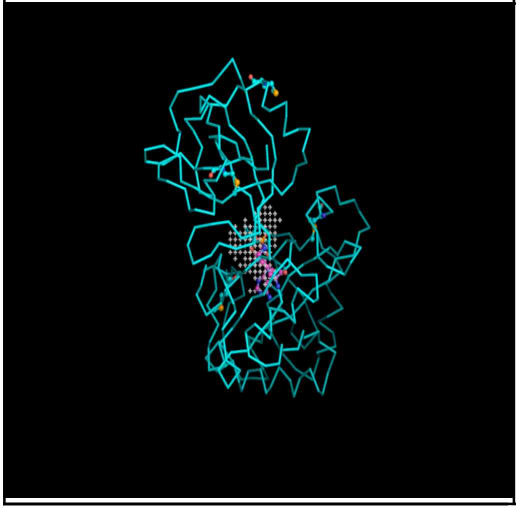	Rv0938	1vs0	A	r1	APK	0.929	0.565

37	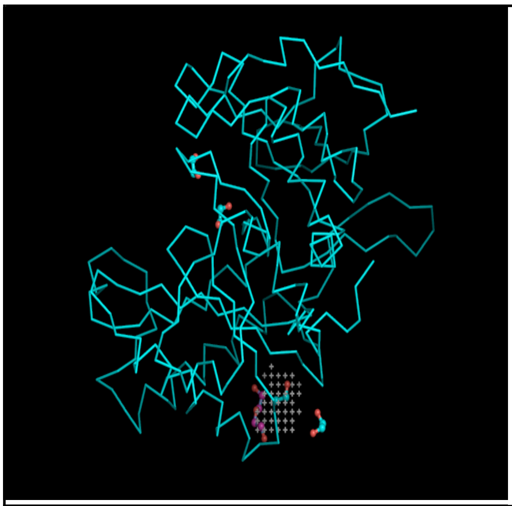	Rv0938	2r9l	A	r16	APK	0.443	0.147
38	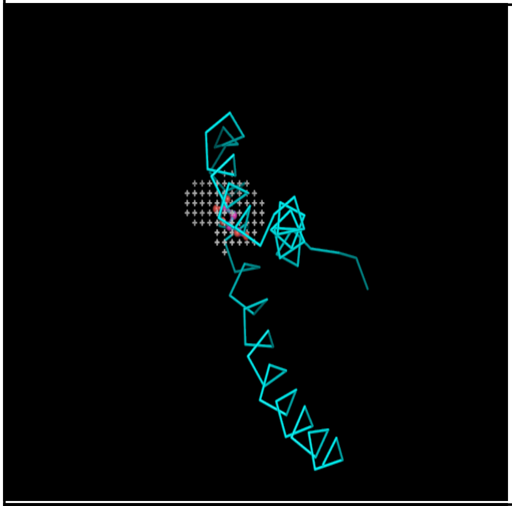	Rv0948c	2vkl	A	r1	MLT	1	0.092
39	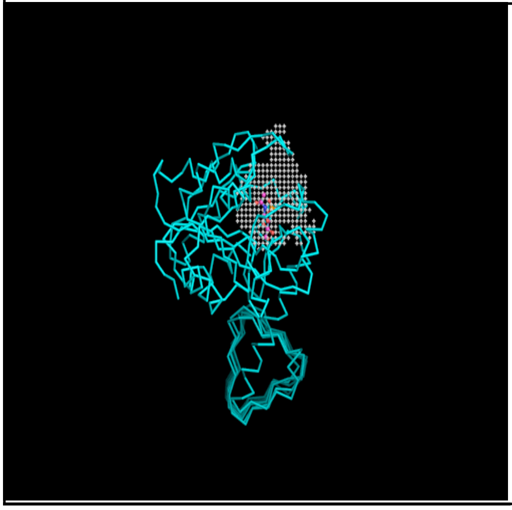	Rv1018c	2qkx	A	r1	UD1	0.984	0.188

40	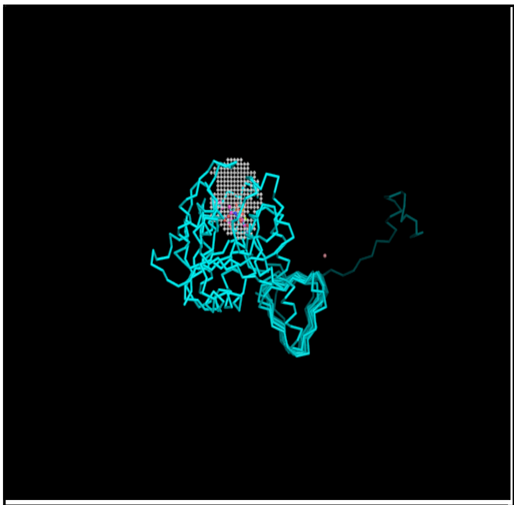	Rv1018c	3dj4	A	r1	UD1	1	0.23
41	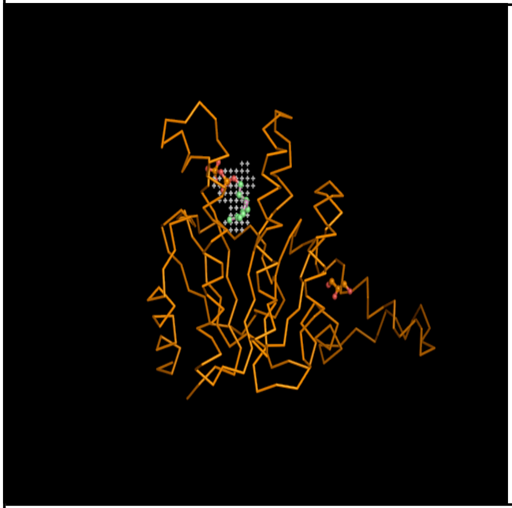	Rv1086	2vg0	A	r1	GPP	0.63	0.489
42	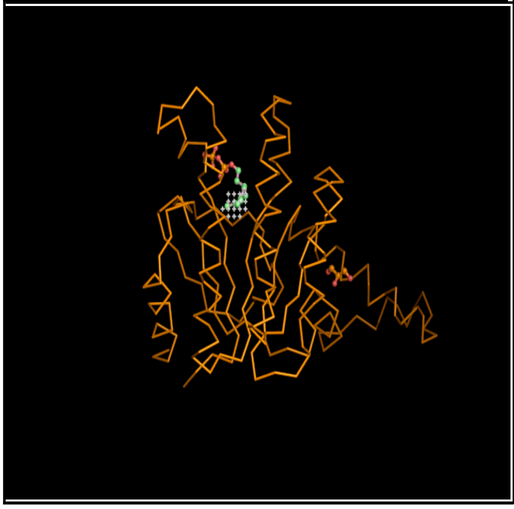	Rv1086	2vg0	A	r3	GPP	0.721	0.433

43	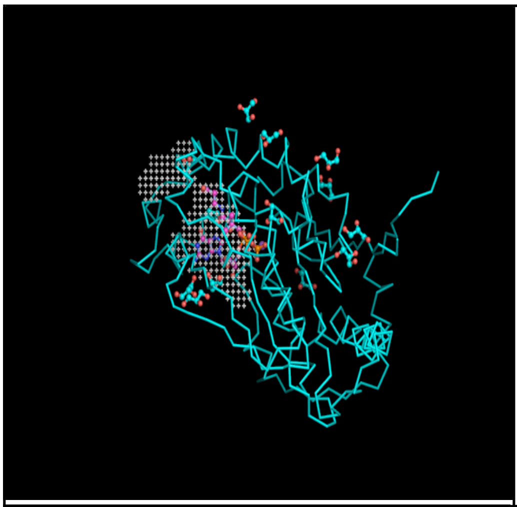	Rv1092c	3aez	A	r1	GDP	0.972	0.08
44	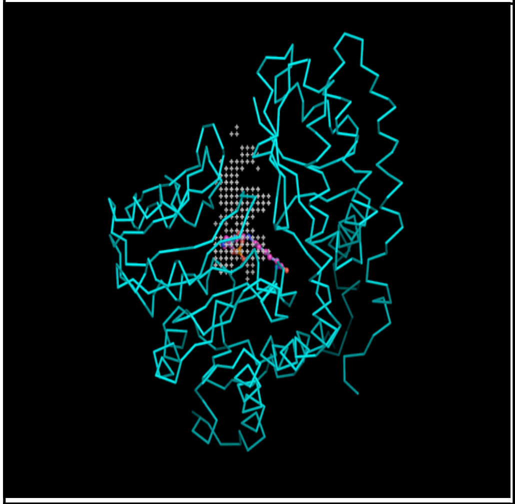	Rv1093	3h7f	A	r12	LLP	0.852	0.253
45	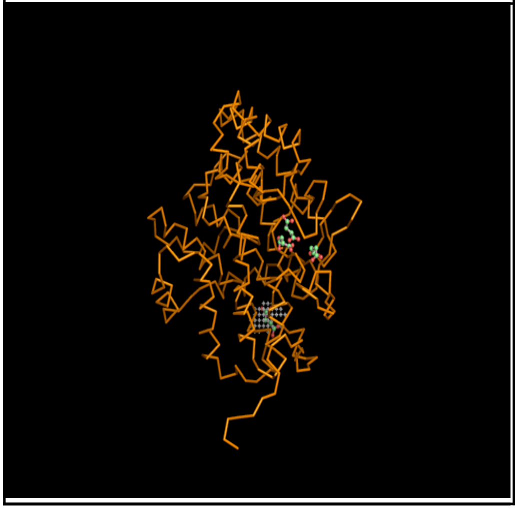	Rv1131	3hwk	A	r14	SIN	0.776	0.076

46	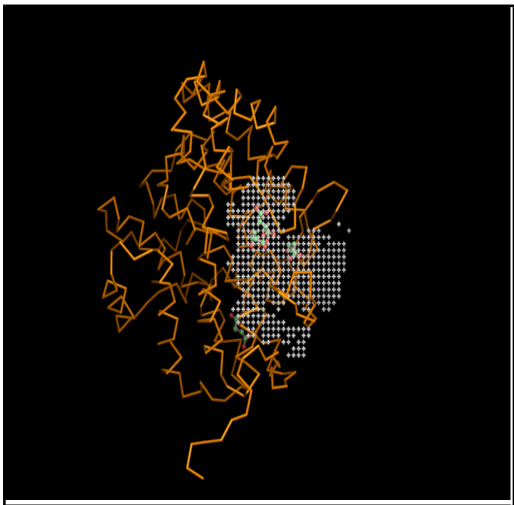	Rv1131	3hwk	A	r3	SIN	0.848	0.133
47	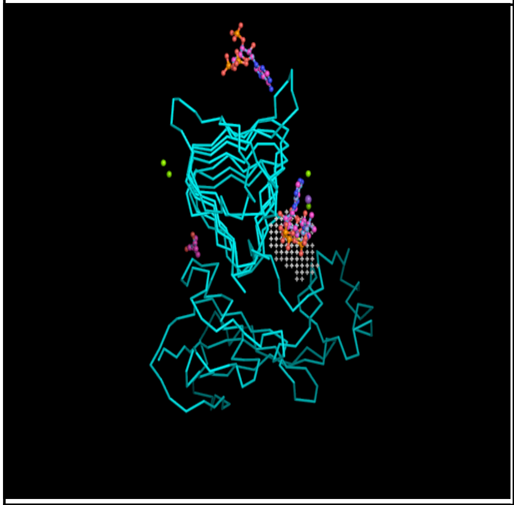	Rv1201c	3fsy	A	r12	SCA	0.537	0.191
48	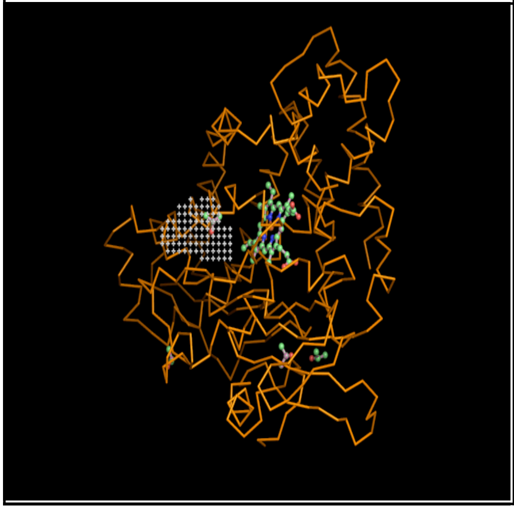	Rv1256c	2uuq	A	r2	IPA	0.35	0.186

49	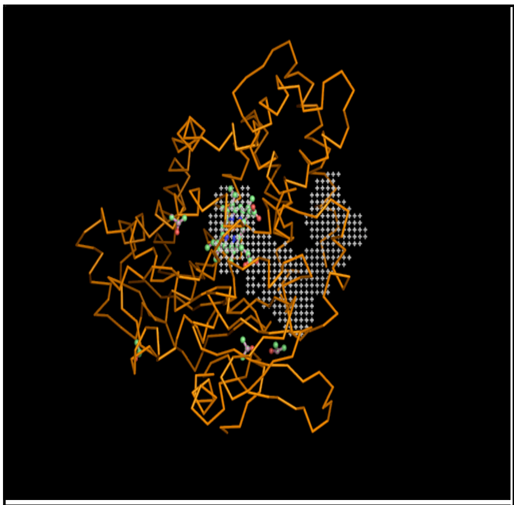	Rv1256c	2uuq	A	r3	HEM	0.964	0.14
50	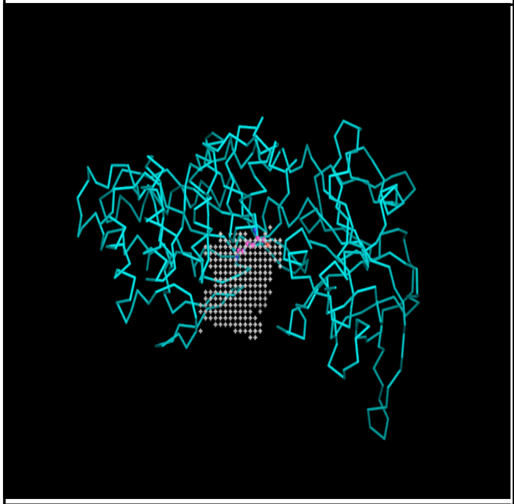	Rv1293	2o0t	A	r3	LLP	1	0.024
51	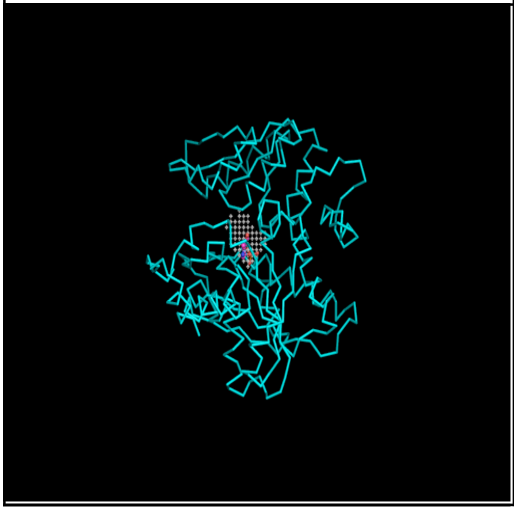	Rv1295	2d1f	A	r6	PLP	0.744	0.383

52	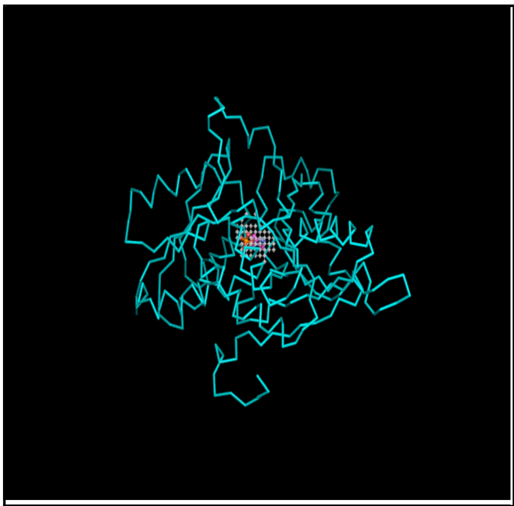	Rv1335	3dwg	A	r3	PLP	0.533	0.362
53	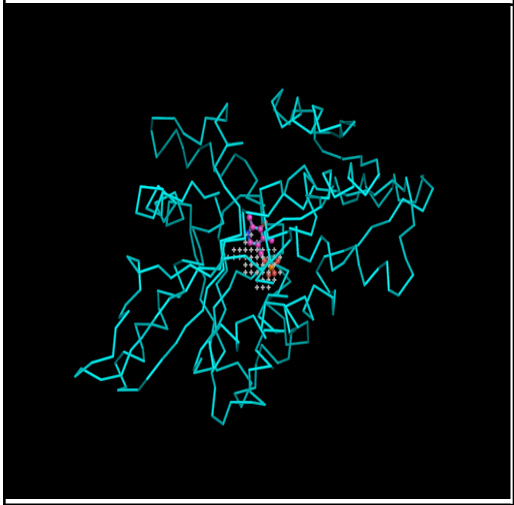	Rv1335	3dwg	B	r2	PLP	0.554	0.289
54	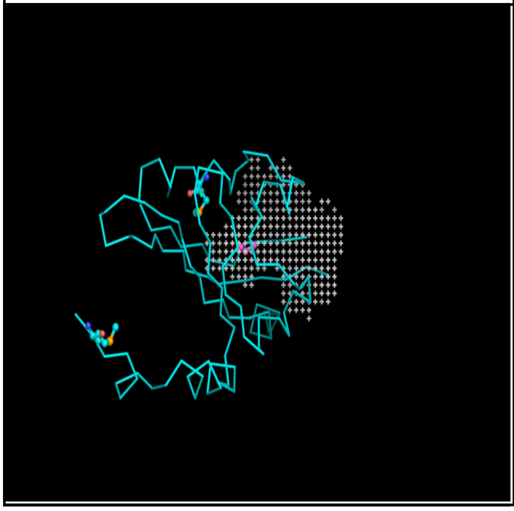	Rv1364c	3kx0	X	r2	PLM	0.917	0.124

55	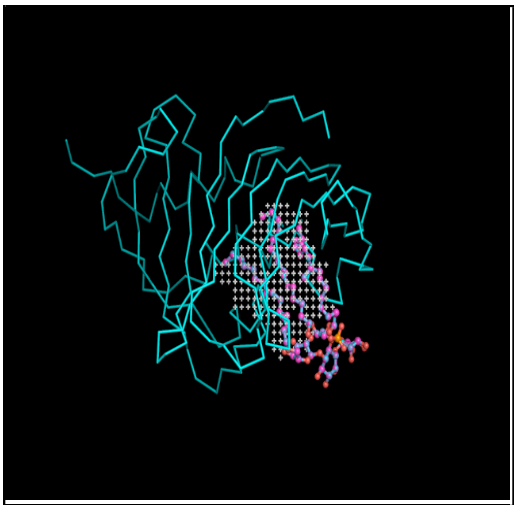	Rv1411c	3mha	A	r1	Z69	1	0.447
56	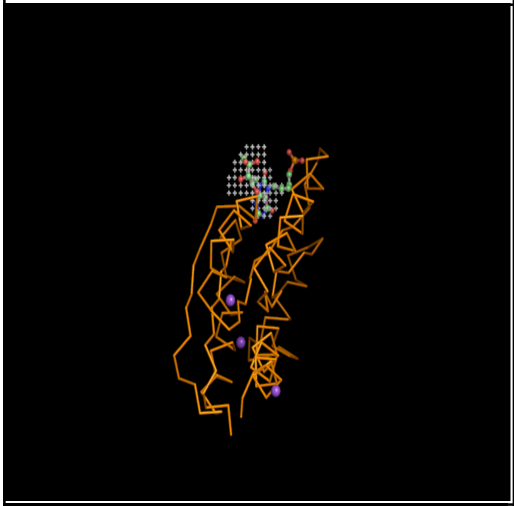	Rv1416	2c92	A	r2	TP6	0.655	0.619
57	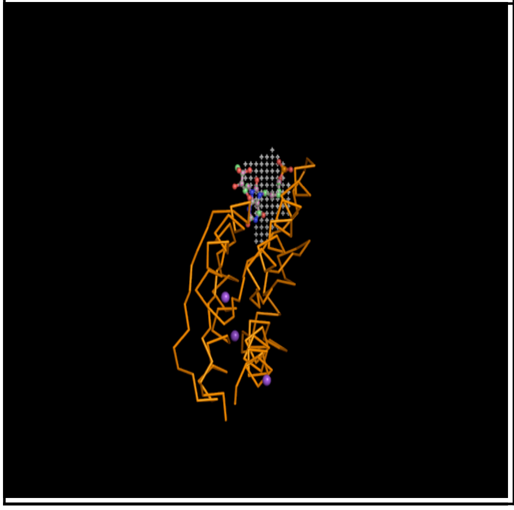	Rv1416	2c92	A	r9	TP6	0.549	0.379

58	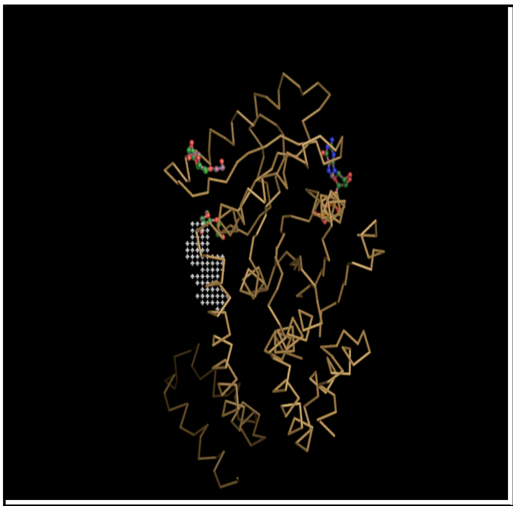	Rv1496	3p32	A	r10	PGE	0.405	0.264
59	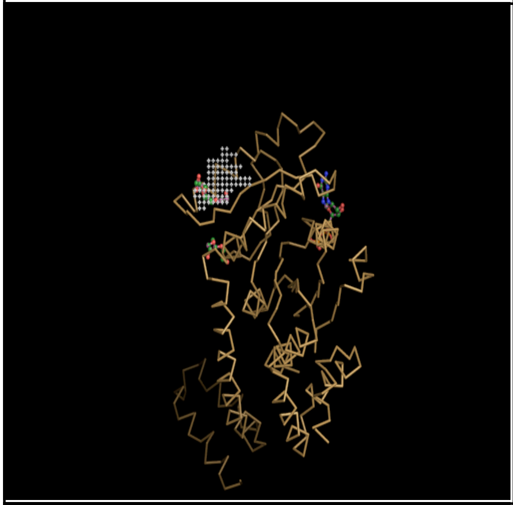	Rv1496	3p32	A	r11	PGE	0.407	0.37
60	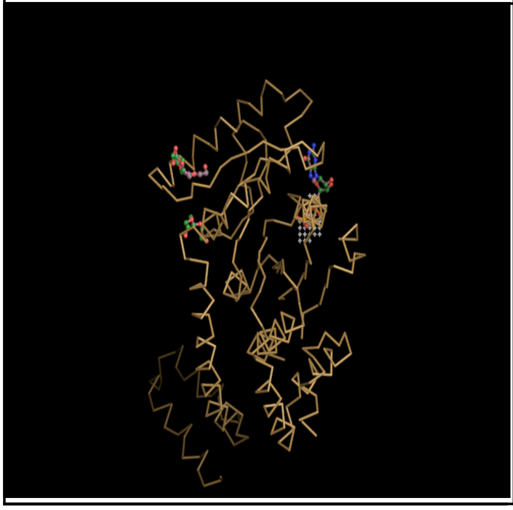	Rv1496	3p32	A	r21	GDP	0.895	0.351

61	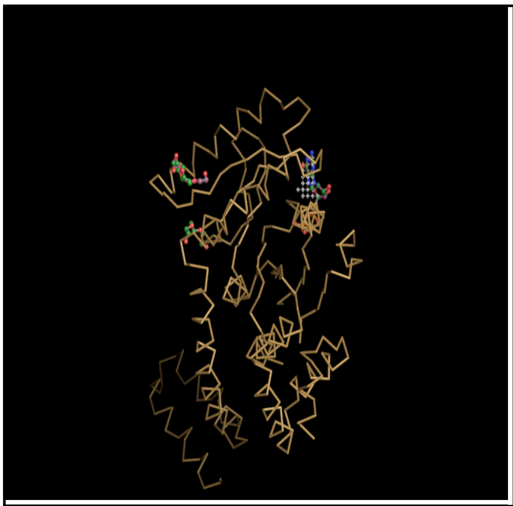	Rv1496	3p32	A	r22	GDP	1	0.393
62	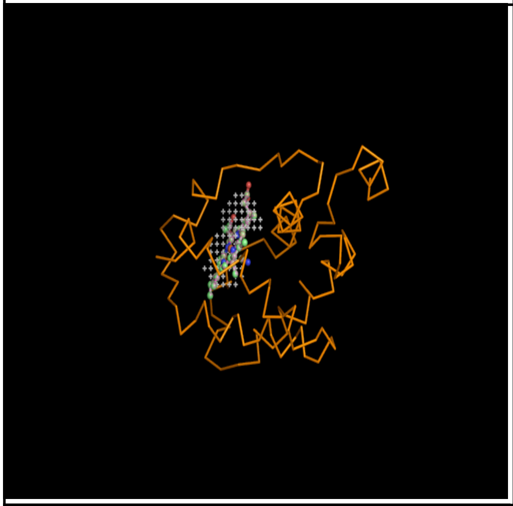	Rv1542c	2gkm	A	r1	HEM	0.853	0.746
63	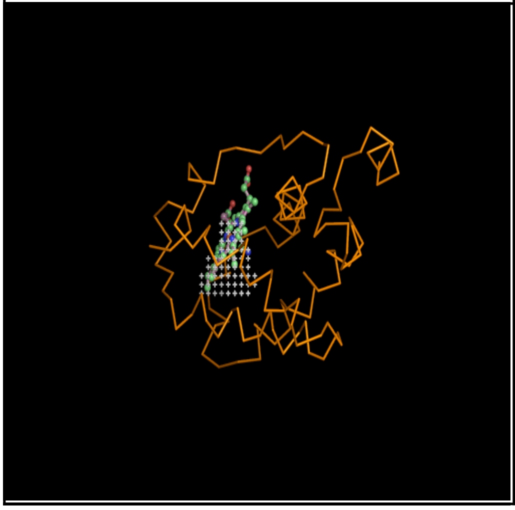	Rv1542c	2gkm	A	r2	HEM	0.673	0.651

64	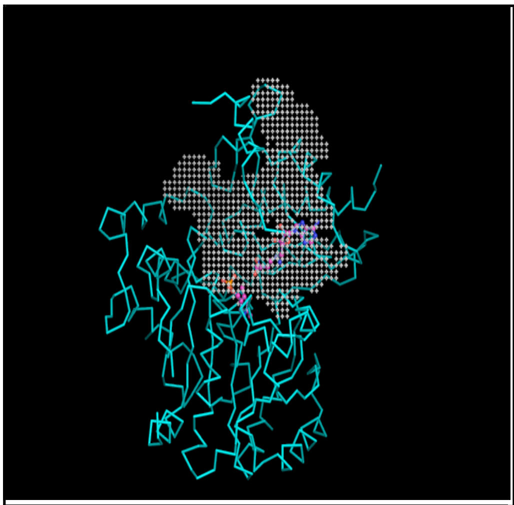	Rv1568	3lv2	A	r1	SFG	1	0.018
65	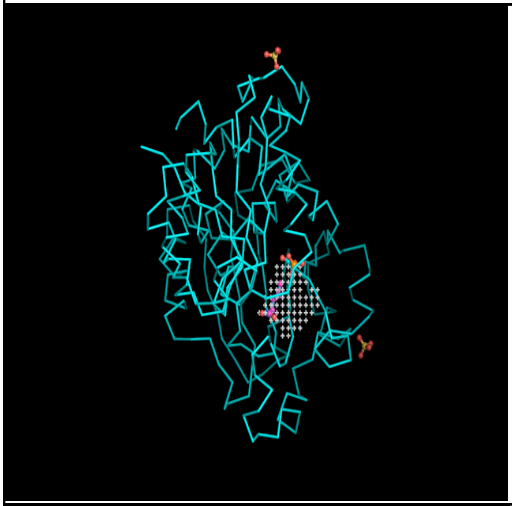	Rv1656	2i6u	A	r4	NVA	1	0.19
66	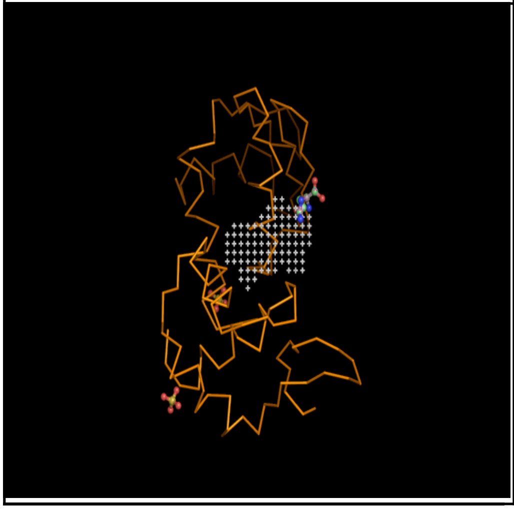	Rv1657	3lap	A	r1	GGB	0.8	0.137

67	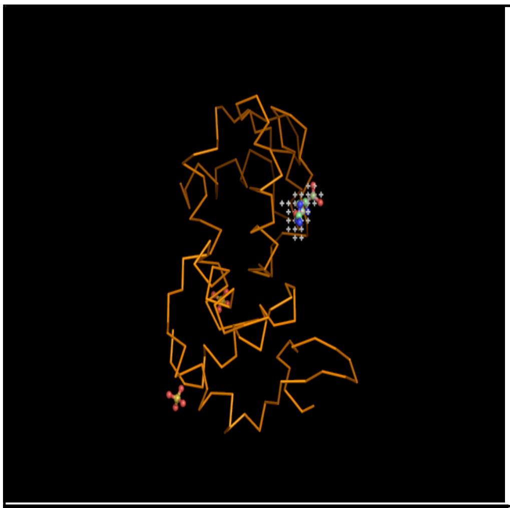	Rv1657	3lap	A	r9	GGB	1	0.761
68	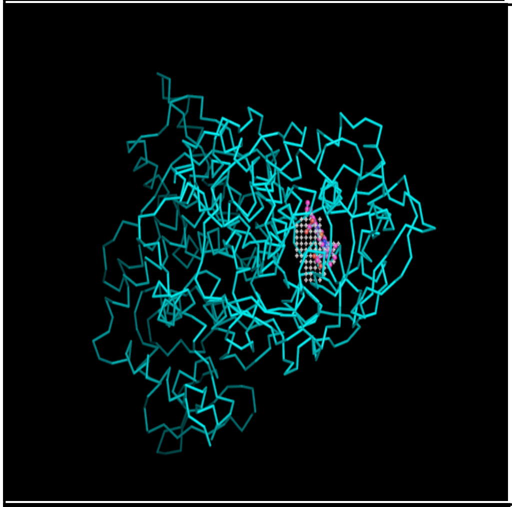	Rv1908c	2cca	A	r16	HEM	0.953	0.475
69	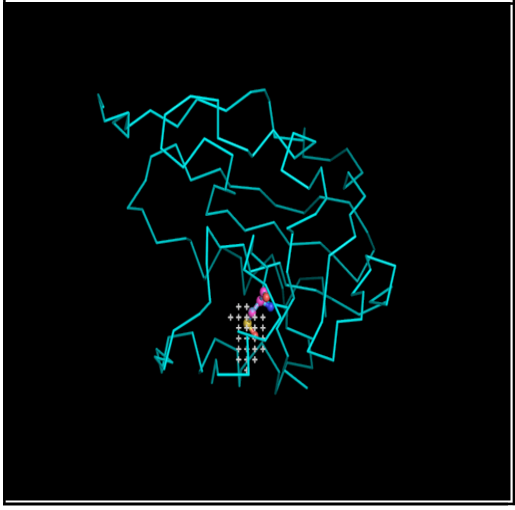	Rv1932	1xvq	A	r2	CSX	0.892	0.515

70	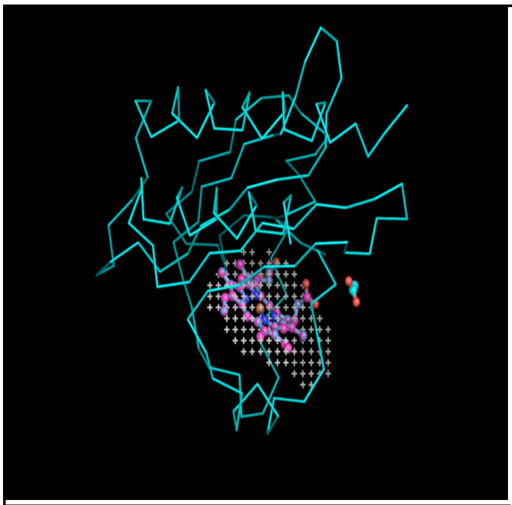	Rv2027c	2vzw	A	r1	HEM	1	0.811
71	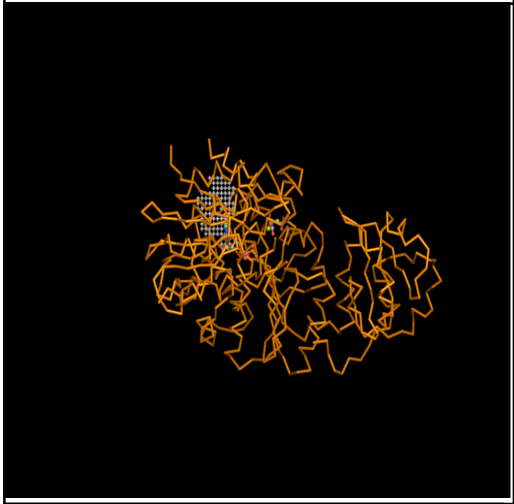	Rv2158c	2xja	A	r2	ADP	0.724	0.417
72	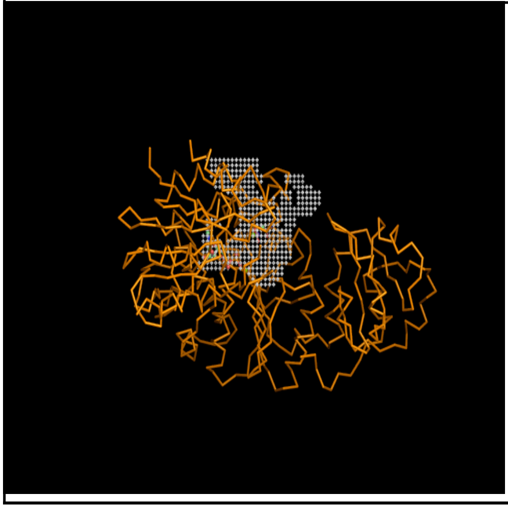	Rv2158c	2xja	A	r3	ADP	1	0.097

73	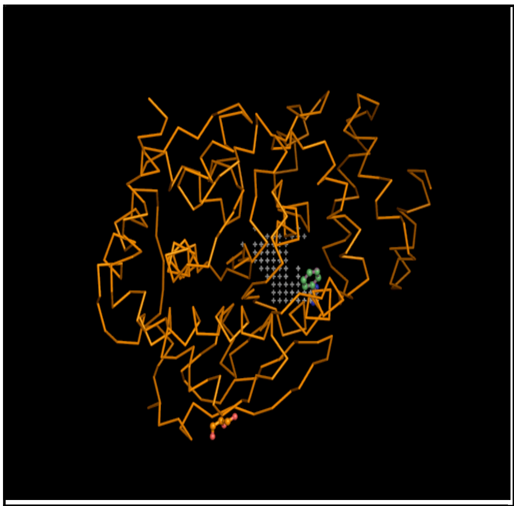	Rv2192c	2bpq	A	r16	BEN	0.516	0.259
74	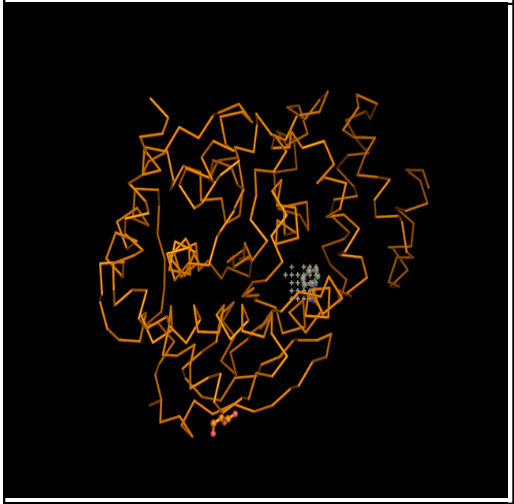	Rv2192c	2bpq	A	r3	BEN	0.853	0.498
75	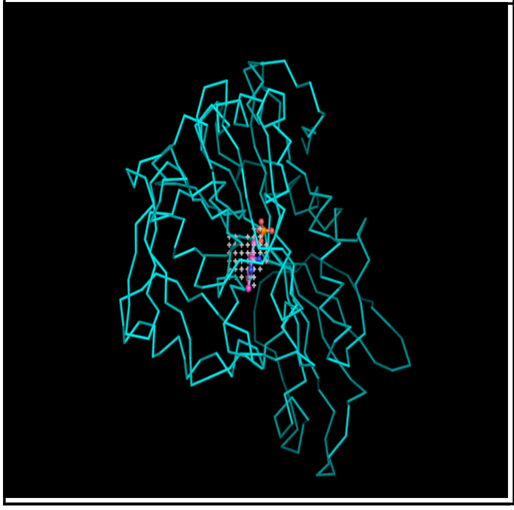	Rv2210c	3ht5	A	r1	PMP	0.874	0.551

76	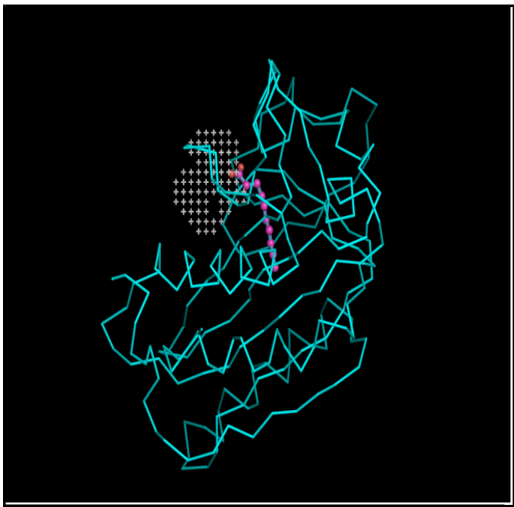	Rv2217	1w66	A	r2	DKA	0.513	0.317
77	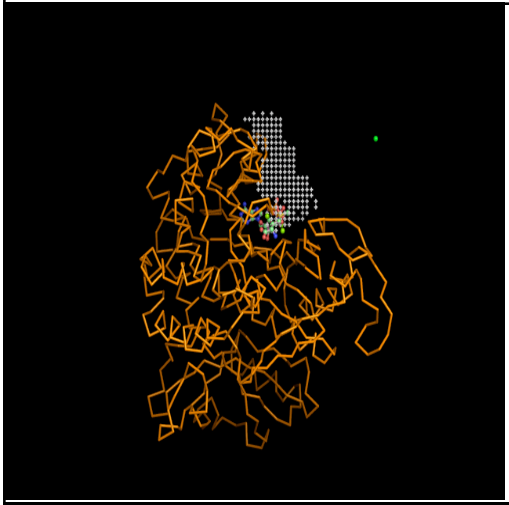	Rv2220	2bvc	A	r25	ADP	0.914	0.2
78	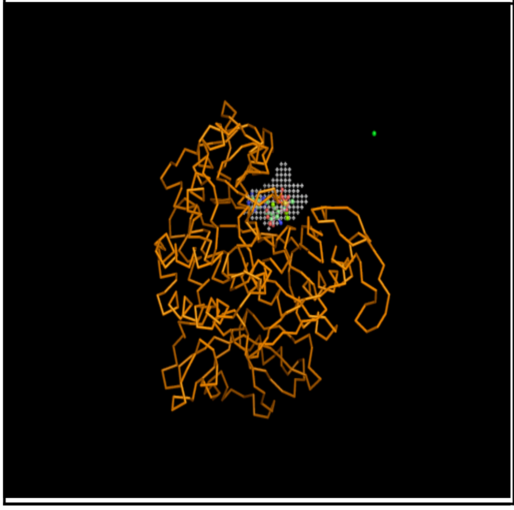	Rv2220	2bvc	A	r3	ADP	1	0.34

79	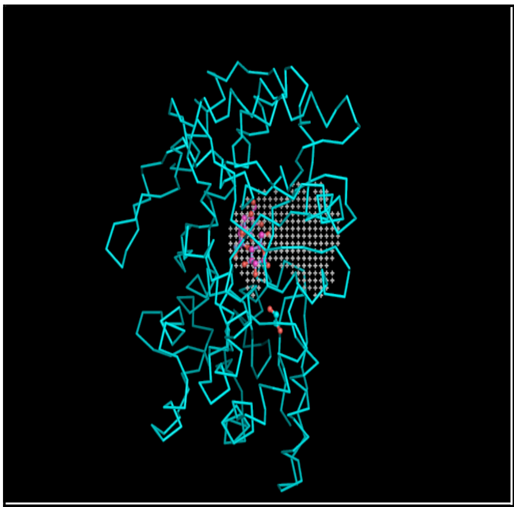	Rv2228c	3hst	A	r2	MLR	0.572	0.445
80	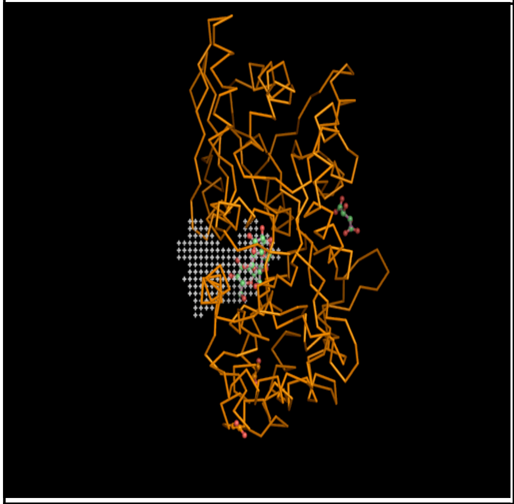	Rv2228c	3hst	C	r1	MLR	0.616	0.369
81	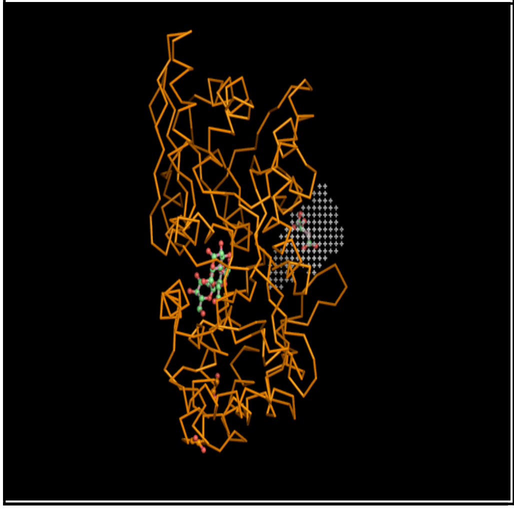	Rv2228c	3hst	C	r5	MLR	0.473	0.124

82	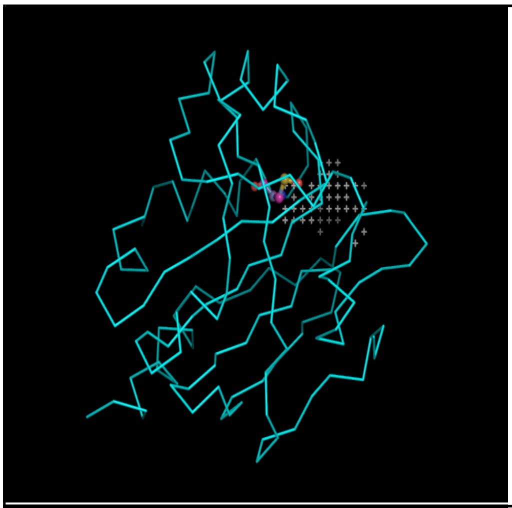	Rv2238c	1xvw	A	r8	CSO	0.783	0.444
83	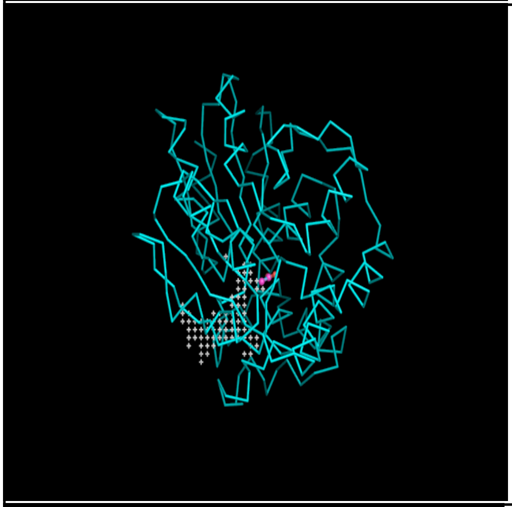	Rv2243	2qc3	A	r8	ACY	0.794	0.068
84	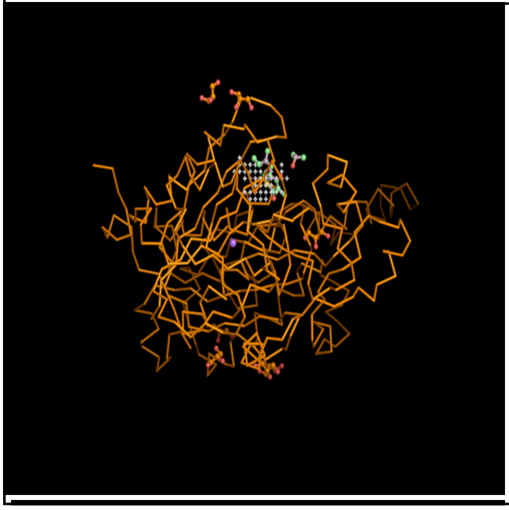	Rv2245	2wge	A	r1	TLM	0.736	0.463

85	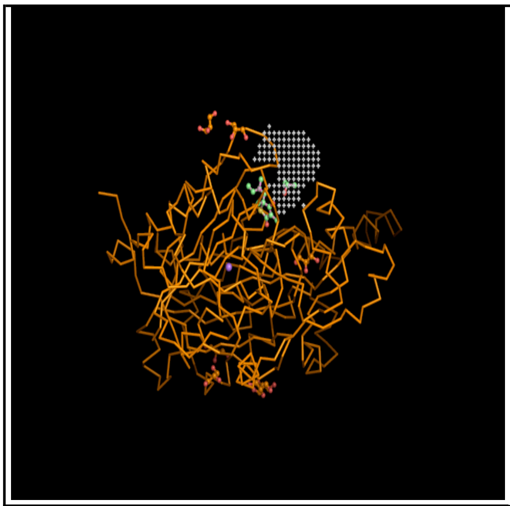	Rv2245	2wge	A	r3	TLM	0.62	0.268
86	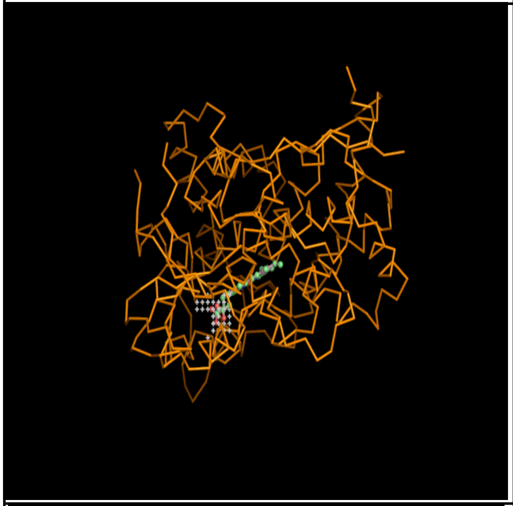	Rv2266	2wm5	A	r13	HEM	0.832	0.299
87	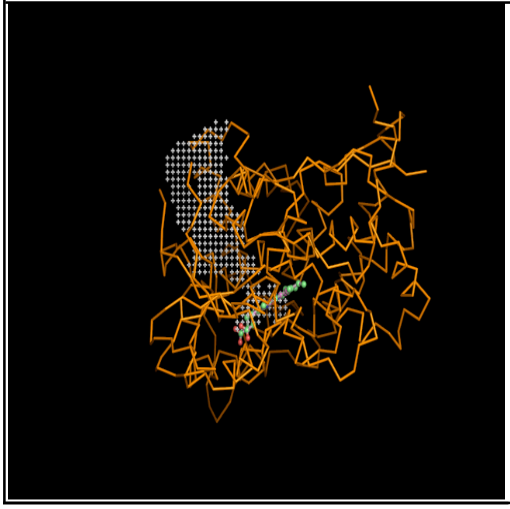	Rv2266	2wm5	A	r2	HEM	0.968	0.145

88	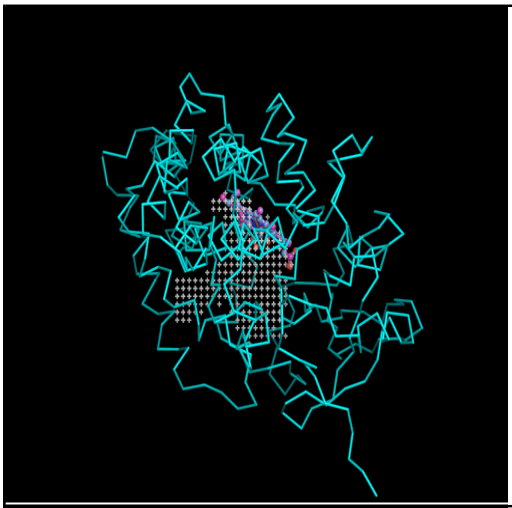	Rv2276	1n40	A	r2	HEM	0.992	0.161
89	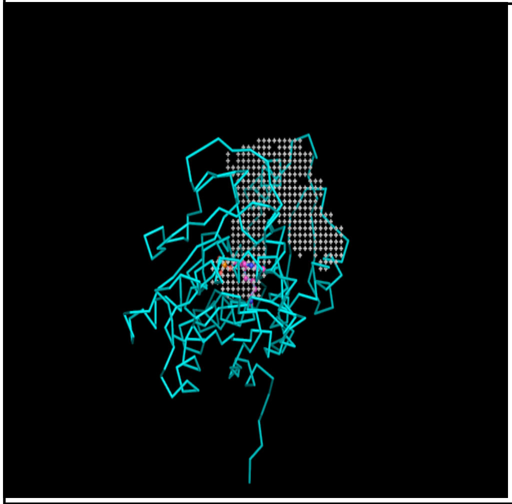	Rv2334	2q3b	A	r2	LLP	1	0.137
90	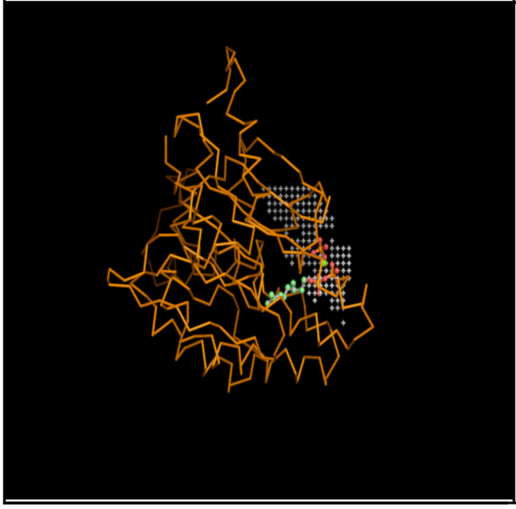	Rv2361c	2vg3	A	r21	GPP	0.694	0.268

91	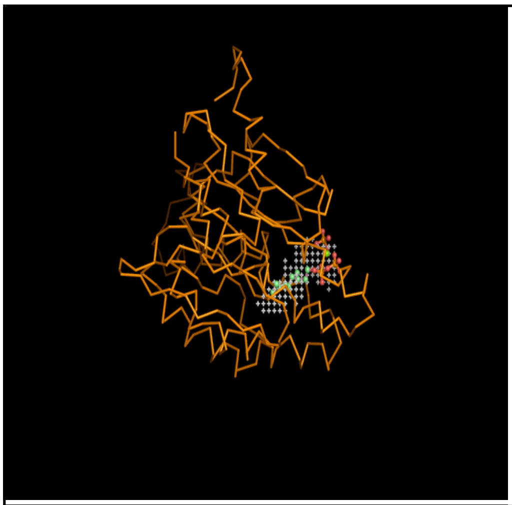	Rv2361c	2vg3	A	r3	GPP	0.913	0.264
92	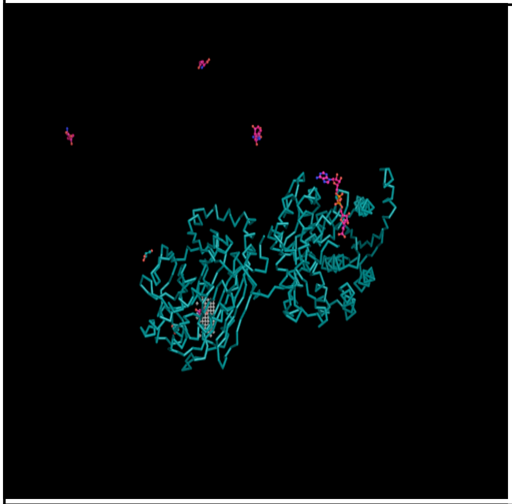	Rv2438c	3dla	A	r11	NXX	0.292	0.244
93	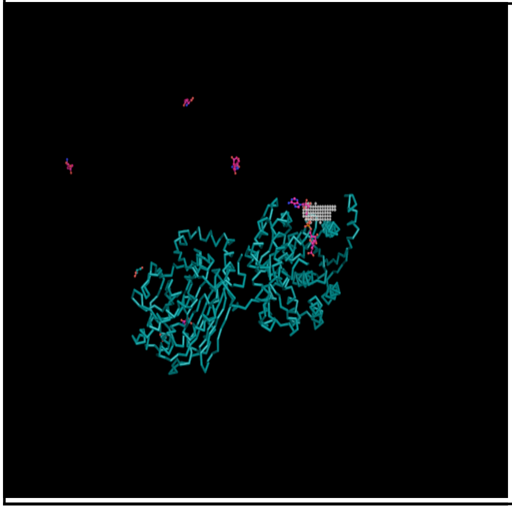	Rv2438c	3dla	A	r1	NXX	0.427	0.259

94	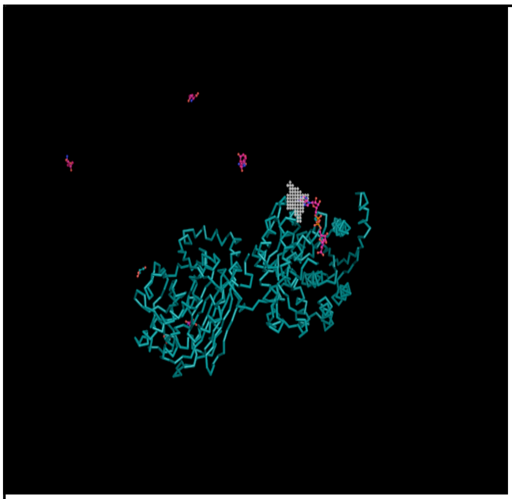	Rv2438c	3dla	A	r32	NXX	0.365	0.365
95	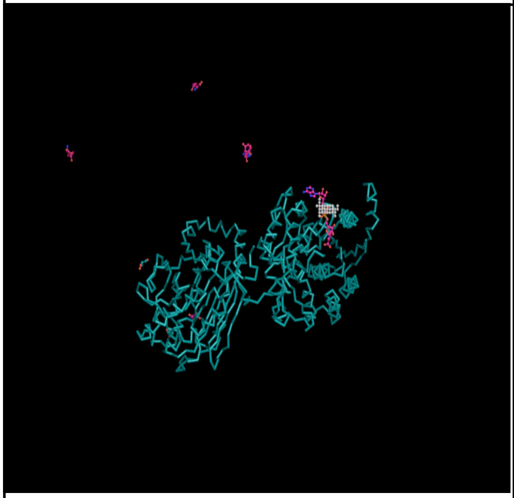	Rv2438c	3dla	A	r36	NXX	0.604	0.111
96	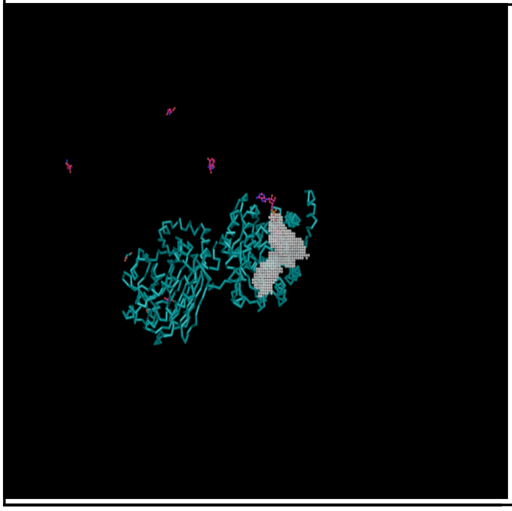	Rv2438c	3dla	A	r9	NXX	0.984	0.028

97	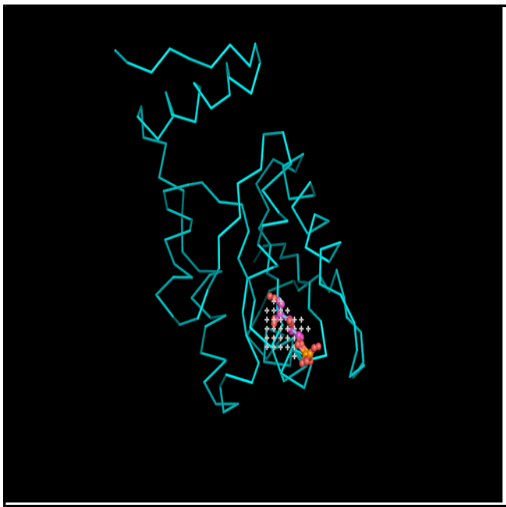	Rv2465c	2vvp	A	r8	5RP	1	1
98	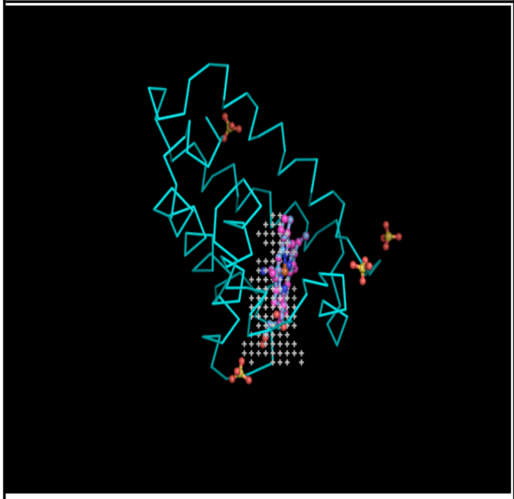	Rv2470	2qrw	A	r1	HEM	0.936	0.541
99	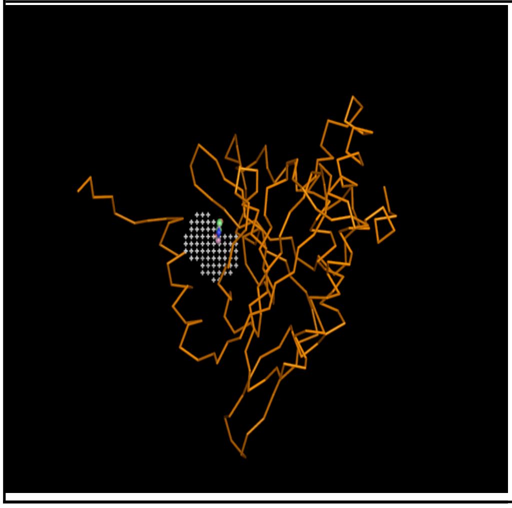	Rv2495c	3l60	A	r2	UNL	0.509	0.015

100	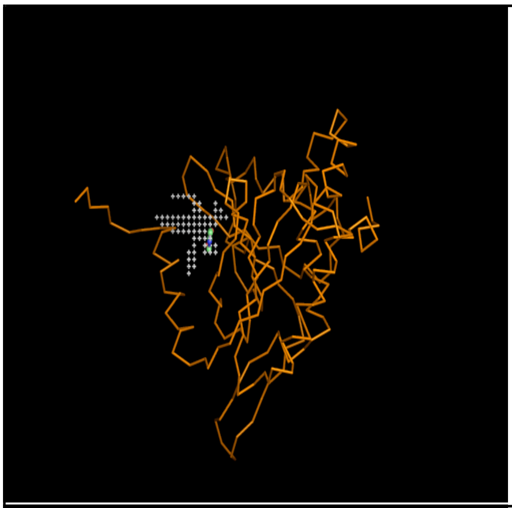	Rv2495c	3l60	A	r8	UNL	1	0.061
101	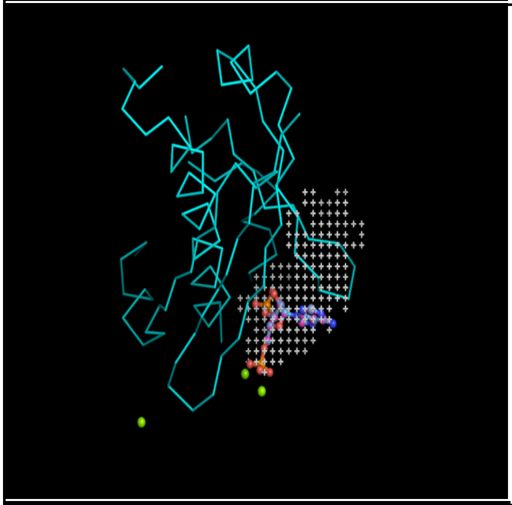	Rv2523c	3hqj	A	r6	COA	1	0.137
102	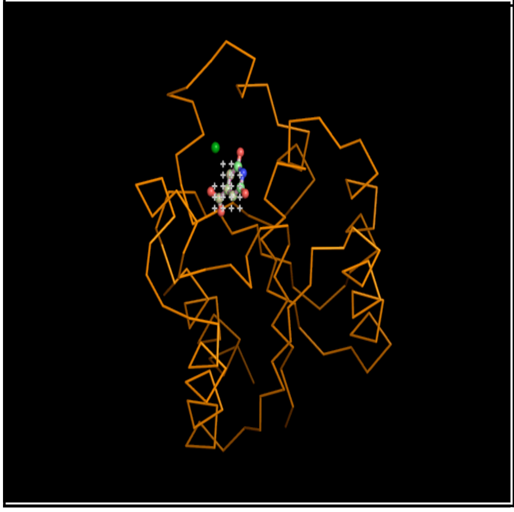	Rv2537c	3n8k	A	r1	D1X	0.914	0.638

103	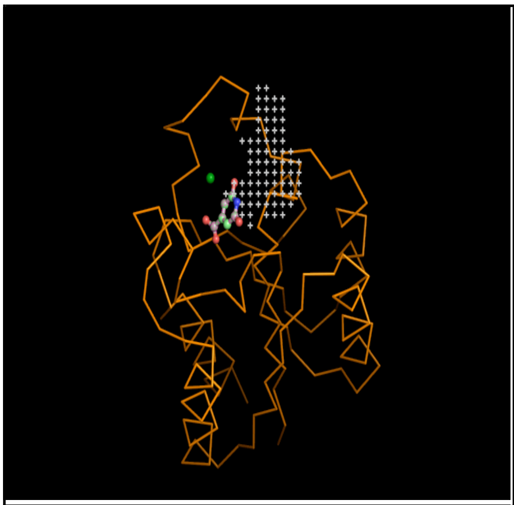	Rv2537c	3n8k	A	r4	D1X	0.578	0.509
104	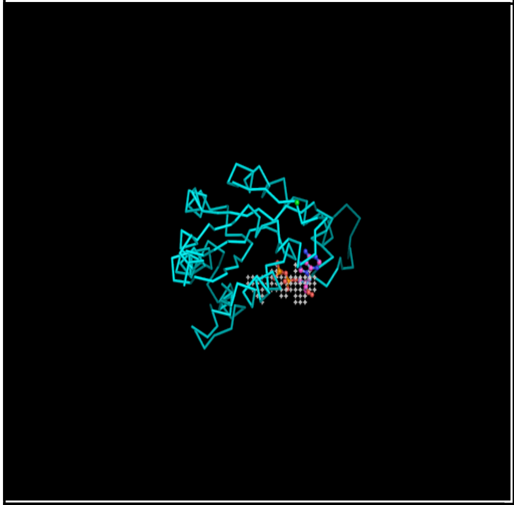	Rv2539c	2iyv	A	r9	ADP	0.696	0.521
105	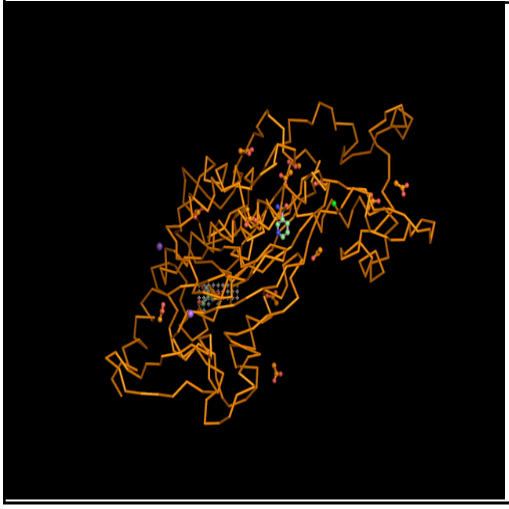	Rv2540c	2qhf	A	r21	TRS	0.516	0.392

106	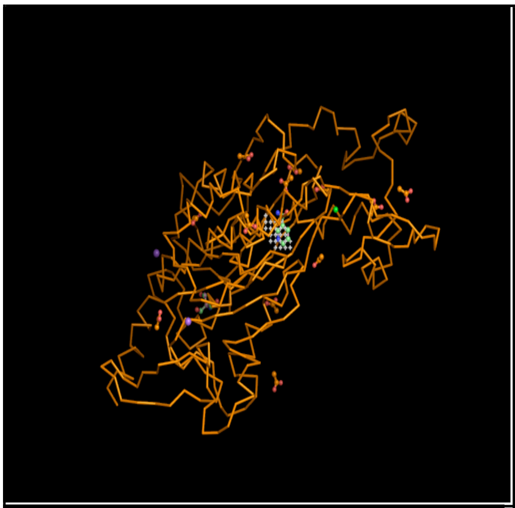	Rv2540c	2qhf	A	r7	NCA	0.849	0.797
107	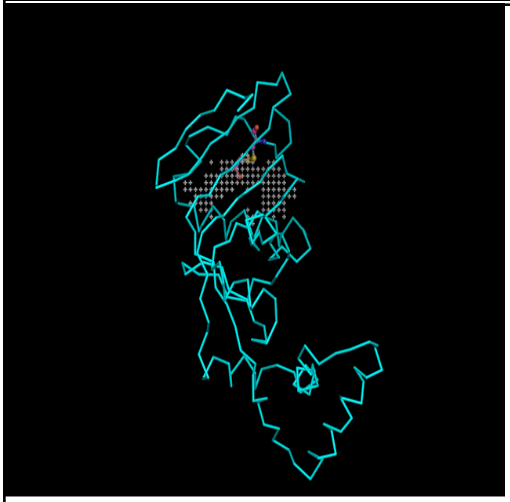	Rv2607	2a2j	A	r9	CME	0.775	0.159
108	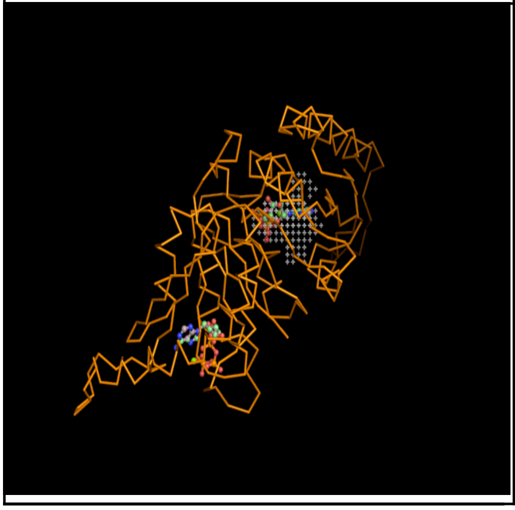	Rv2623	3cis	A	r19	ATP	0.881	0.42

109	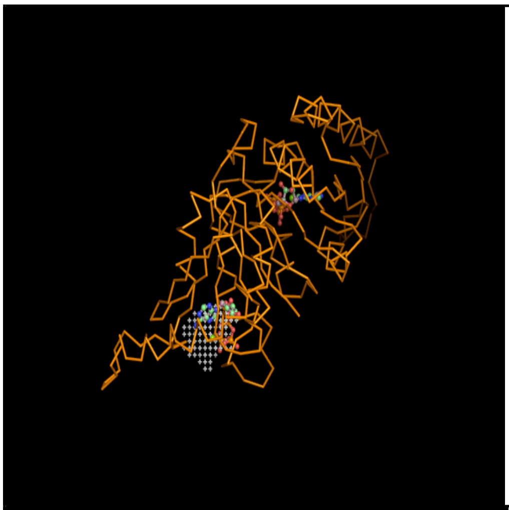	Rv2623	3cis	A	r2	ATP	0.818	0.131
110	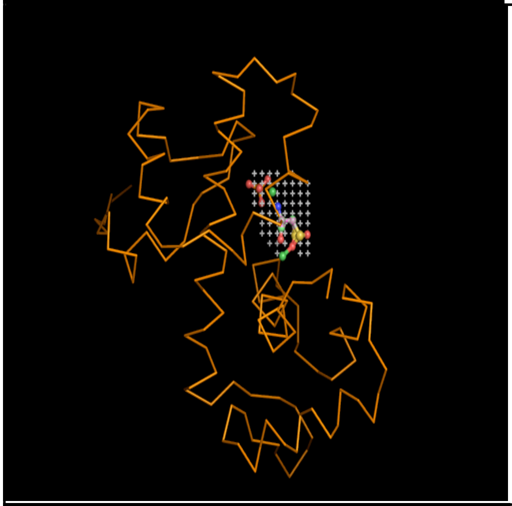	Rv2711	2isy	A	r2	CSO	0.552	0.293
111	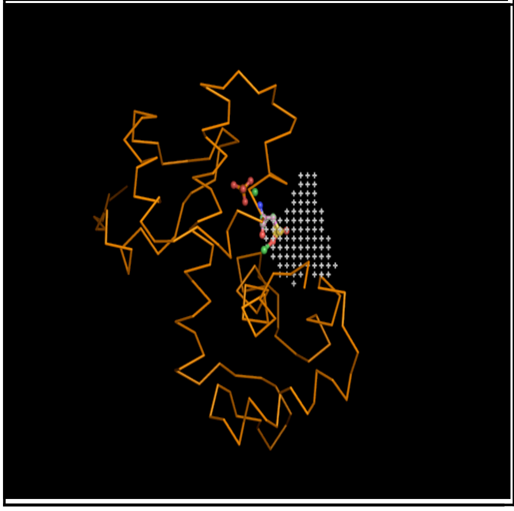	Rv2711	2isy	A	r3	CSO	0.807	0.275

112	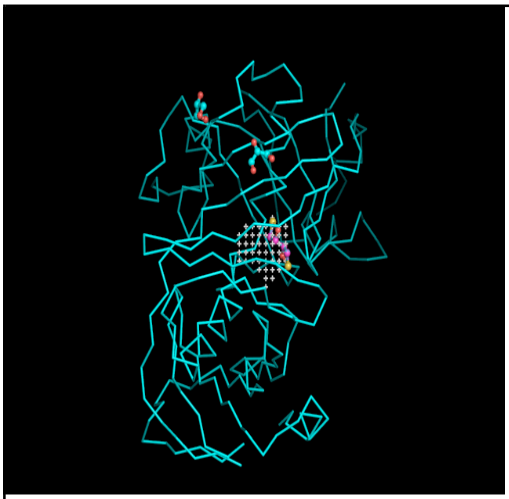	Rv2726c	3fve	A	r1	DTT	0.973	0.229
113	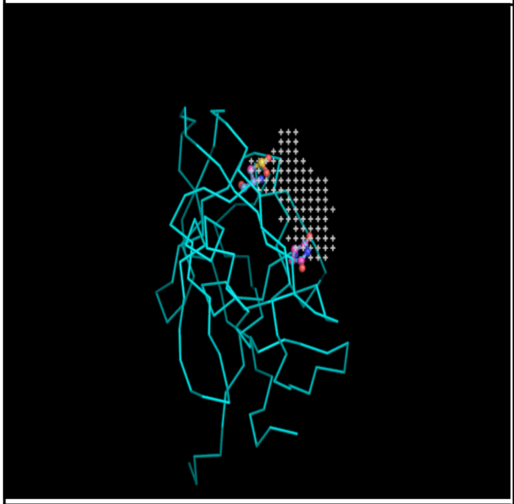	Rv2737c	2imz	A	r1	CSW	0.903	0.251
114	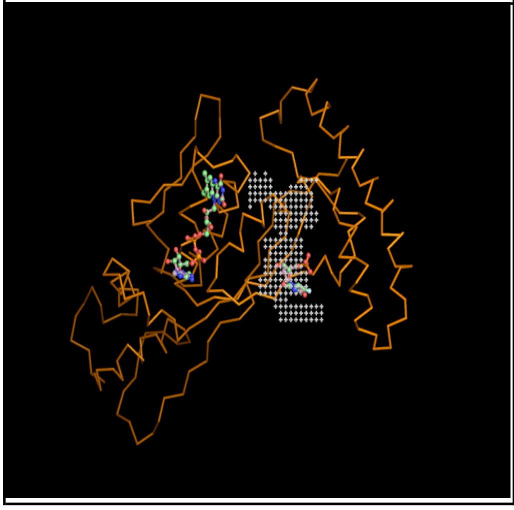	Rv2754c	3gwc	A	r11	UFP	1	0.096

115		Rv2754c	3gwc	A	r6	FAD	1	0.08
116	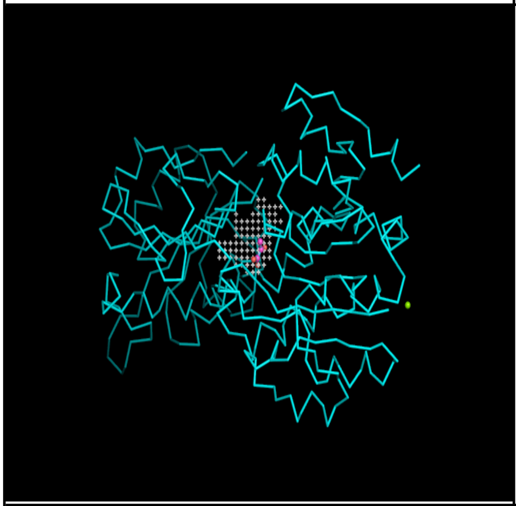	Rv2780	2vhx	A	r5	PYR	1	0.135
117	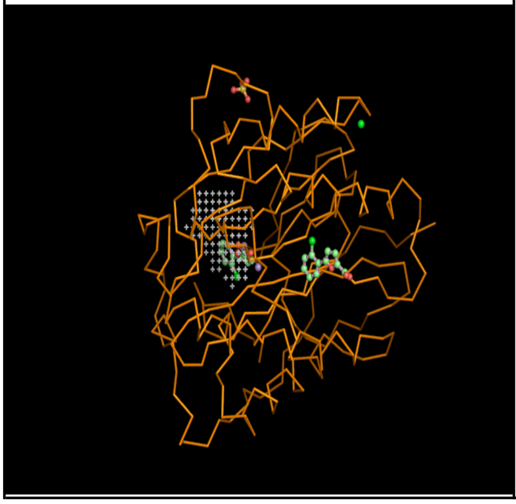	Rv2861c	3iu7	A	r1	FCD	0.657	0.565

118	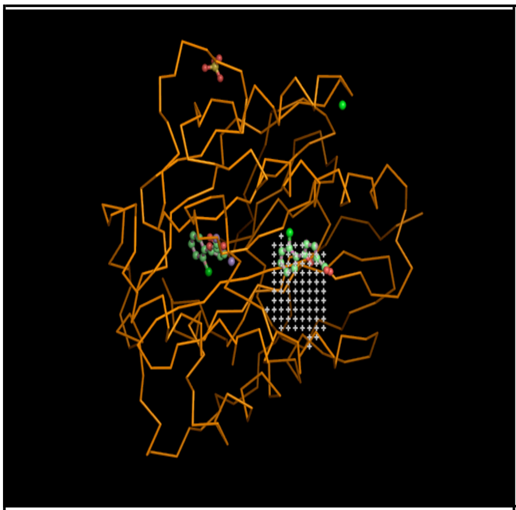	Rv2861c	3iu7	A	r2	FCD	0.403	0.354
119	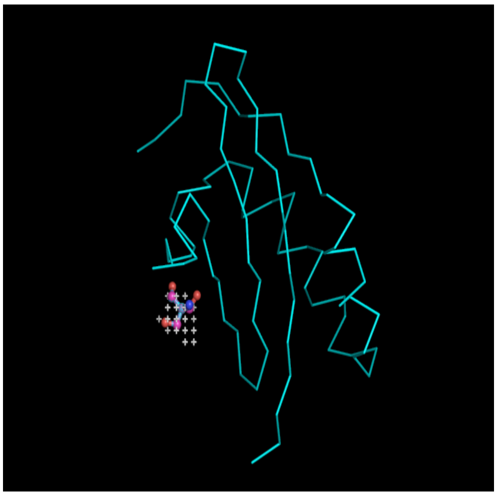	Rv2865	3g5o	C	r3	TRS	0.725	0.629
120	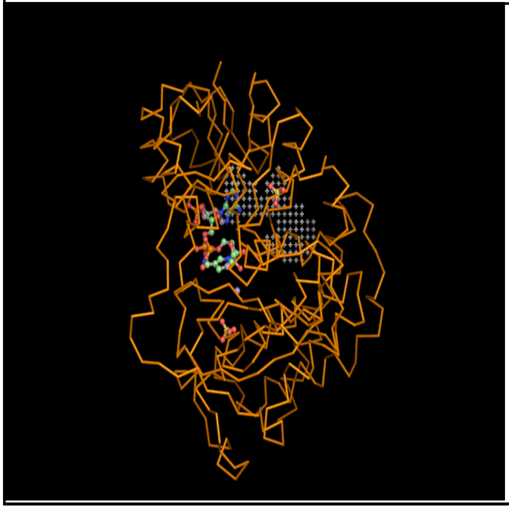	Rv2870c	2jd1	A	r17	NDP	0.683	0.66

121	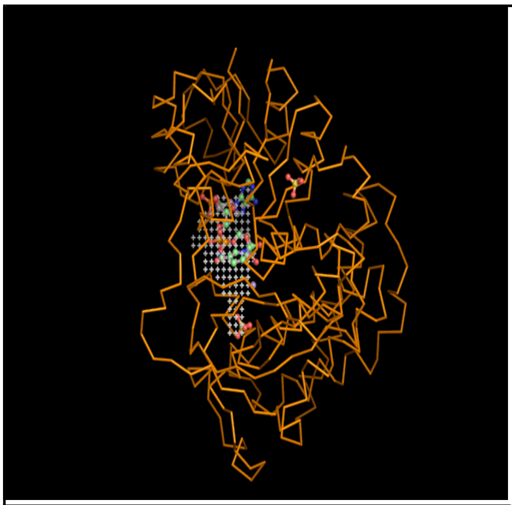	Rv2870c	2jd1	A	r3	NDP	0.991	0.336
122	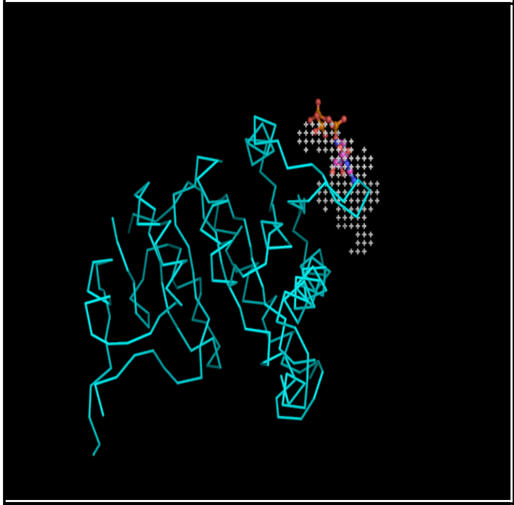	Rv2883c	3nwy	A	r14	GTP	0.529	0.159
123	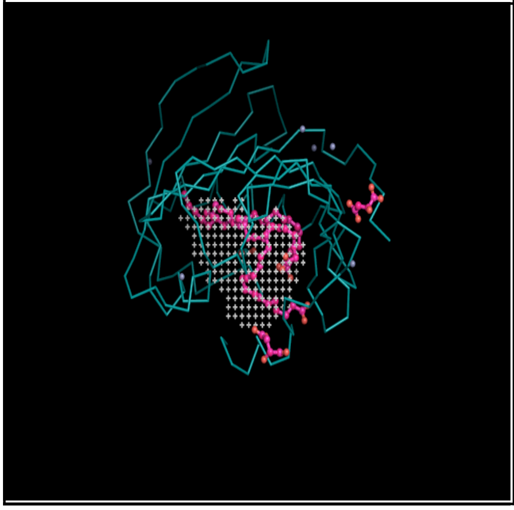	Rv2945c	2byo	A	r12	HXA	0.43	0.297

124	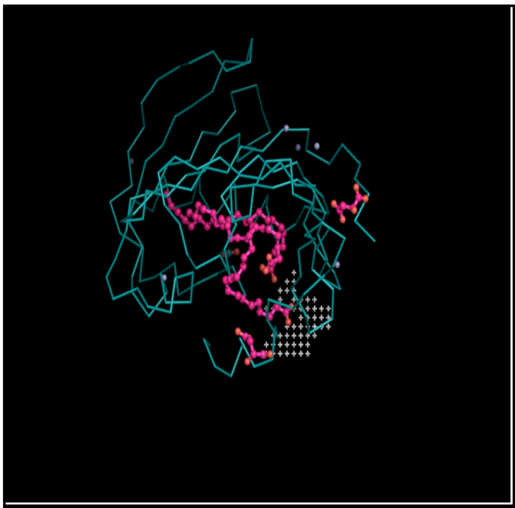	Rv2945c	2byo	A	r15	OAA	0.743	0.168
125	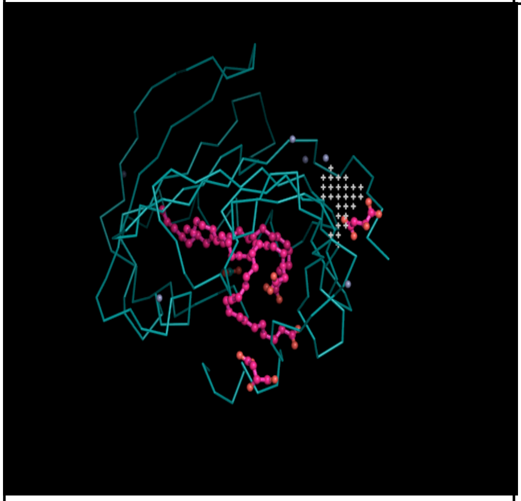	Rv2945c	2byo	A	r1	LNL	1	0.076
126	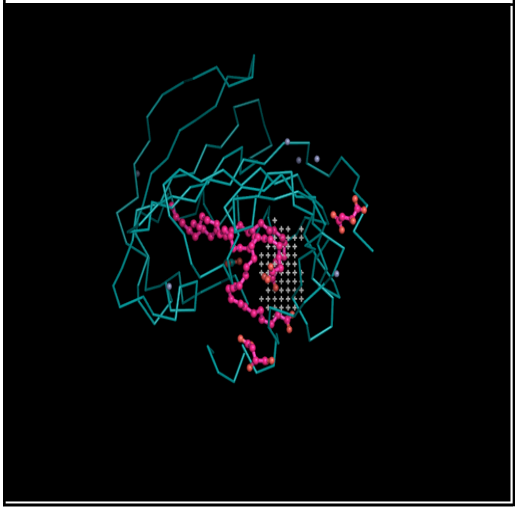	Rv2945c	2byo	A	r2	HXA	0.674	0.555

127	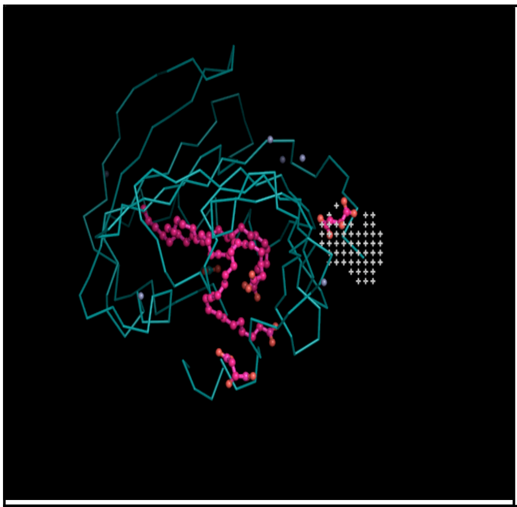	Rv2945c	2byo	A	r4	OAA	1	0.241
128	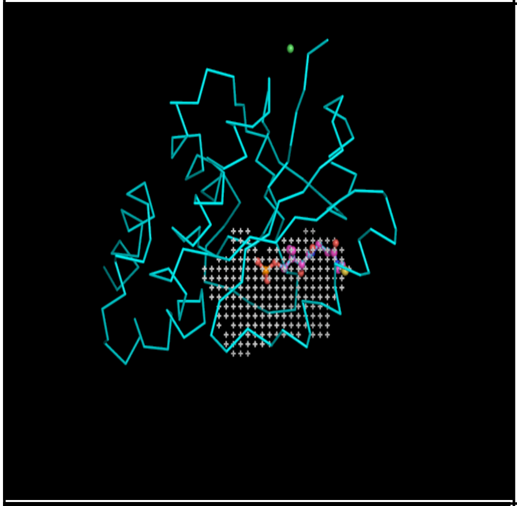	Rv2965c	3nbk	A	r1	PNS	1	0.046
129	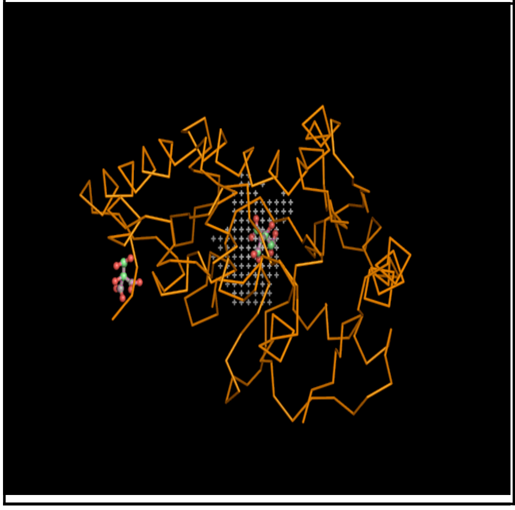	Rv2976c	3a7n	A	r2	FLC	0.6	0.283

130	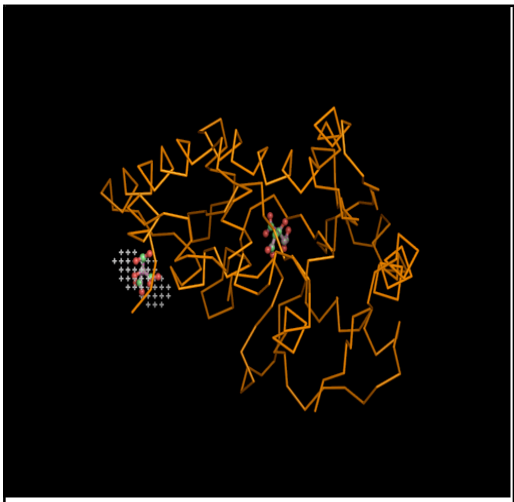	Rv2976c	3a7n	A	r4	FLC	0.403	0.131
131	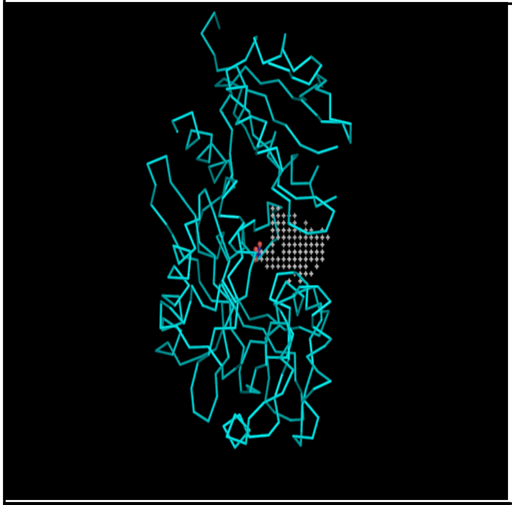	Rv2981c	3lwb	A	r4	NO3	1	0.065
132	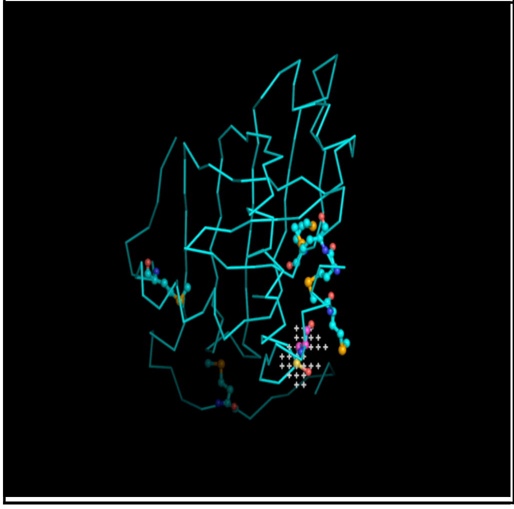	Rv2991	1rfe	A	r15	CSX	0.763	0.573

133	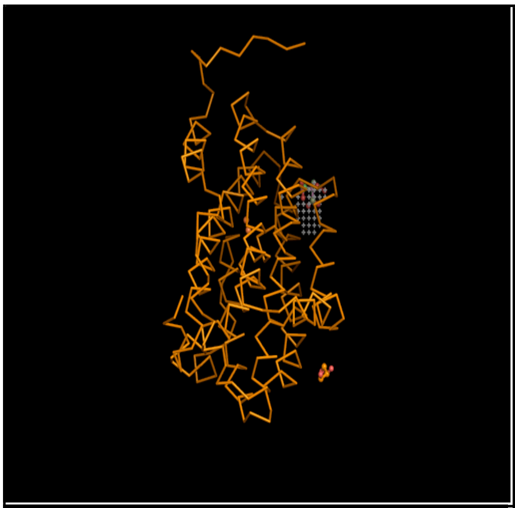	Rv3048c	1uzr	A	r17	CIT	1	0.383
134	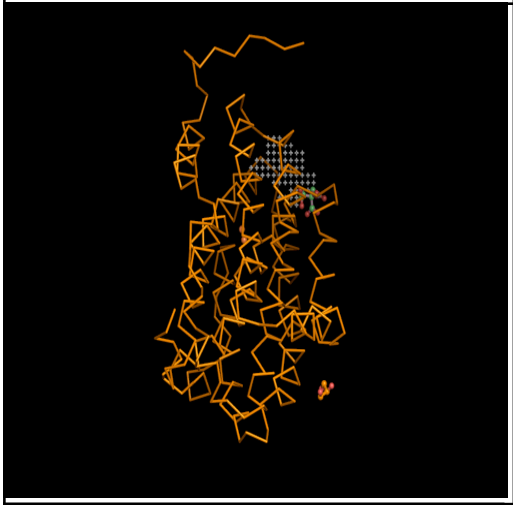	Rv3048c	1uzr	A	r3	CIT	0.684	0.151
135	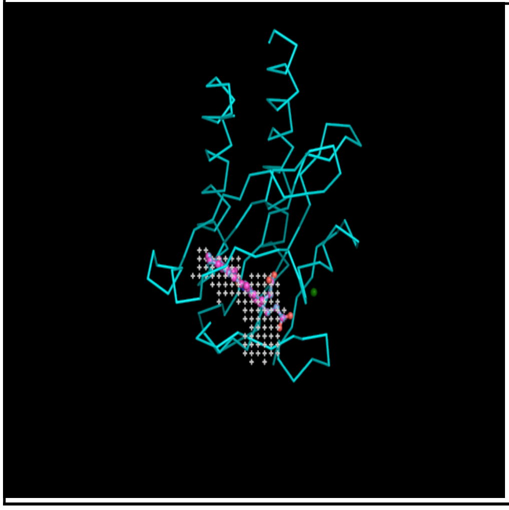	Rv3132c	2w3g	A	r1	HEM	1	0.501

136	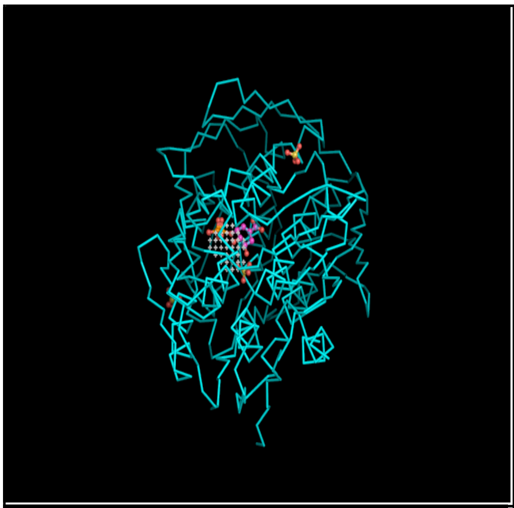	Rv3227	2o0b	A	r2	S3P	0.654	0.4
137	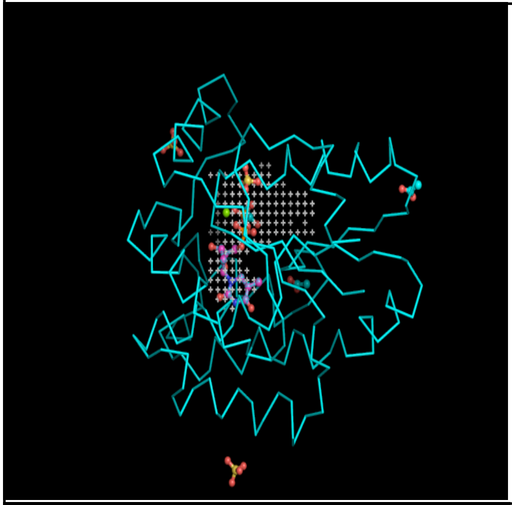	Rv3247c	1gtv	A	r2	TYD	1	0.173
138	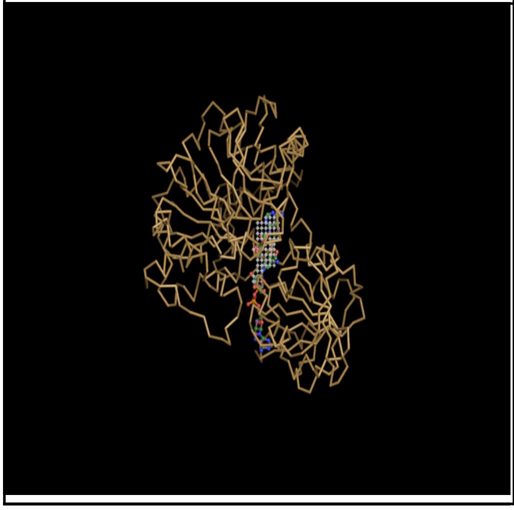	Rv3248c	3ce6	A	r12	NAD	0.695	0.598

139	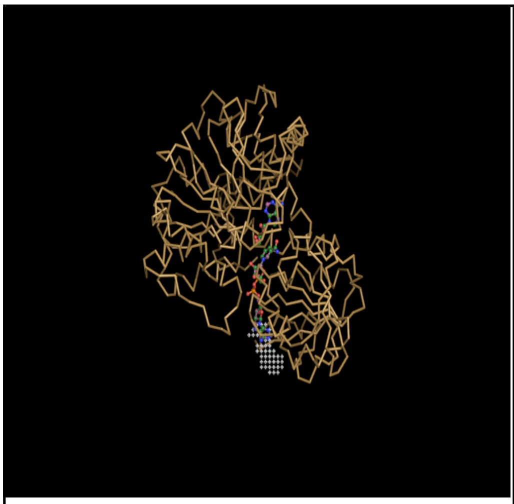	Rv3248c	3ce6	A	r36	ADN	0.555	0.375
140	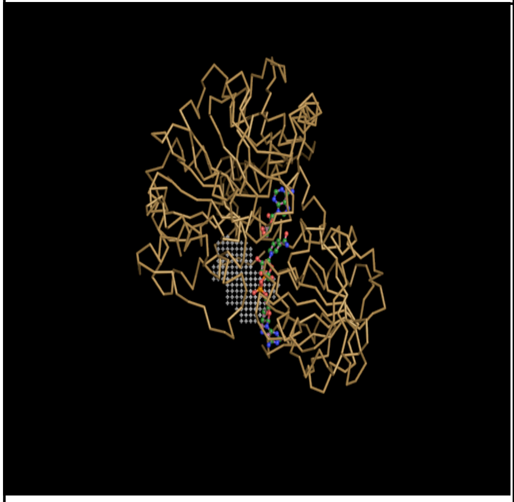	Rv3248c	3ce6	A	r4	NAD	0.446	0.395
141	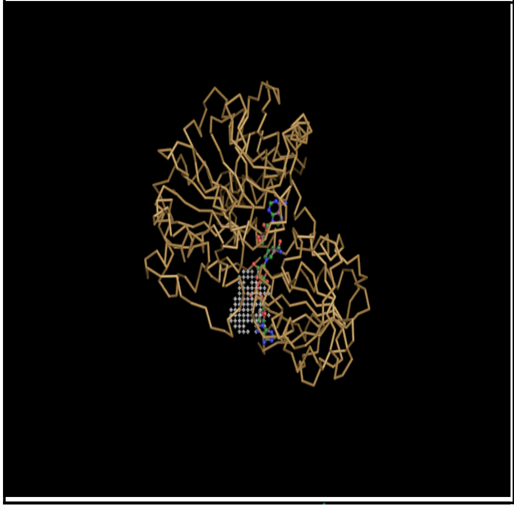	Rv3248c	3ce6	A	r8	ADN	0.513	0.363

142	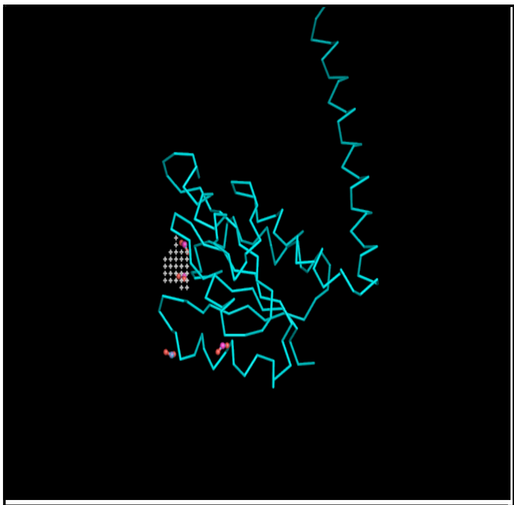	Rv3275c	3lp6	A	r1	FMT	0.665	0.537
143	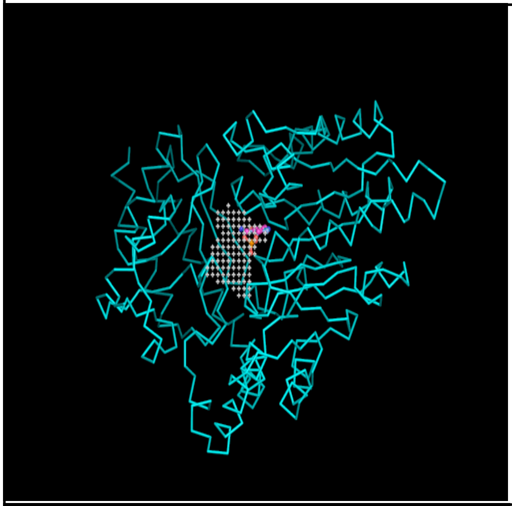	Rv3290c	2cjg	A	r5	PMP	0.84	0.273
144	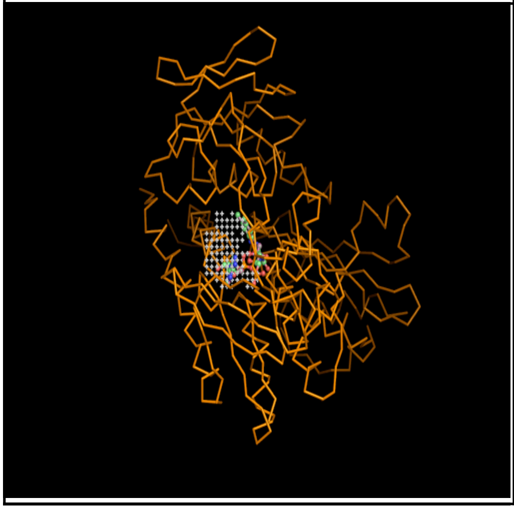	Rv3303c	1xdi	A	r3	FAD	0.742	0.705

145	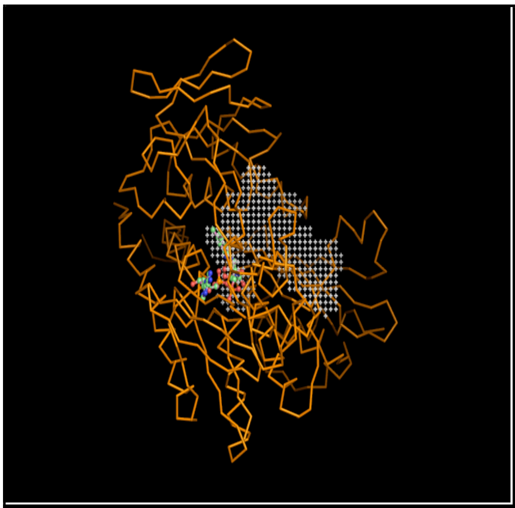	Rv3303c	1xdi	A	r6	FAD	0.97	0.166
146	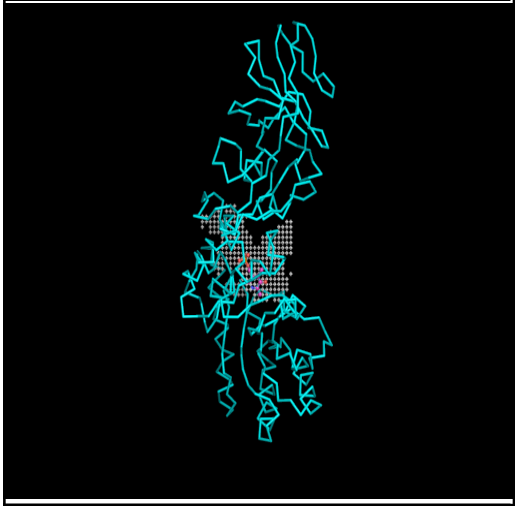	Rv3423c	1xfc	A	r3	PLP	1	0.071
147	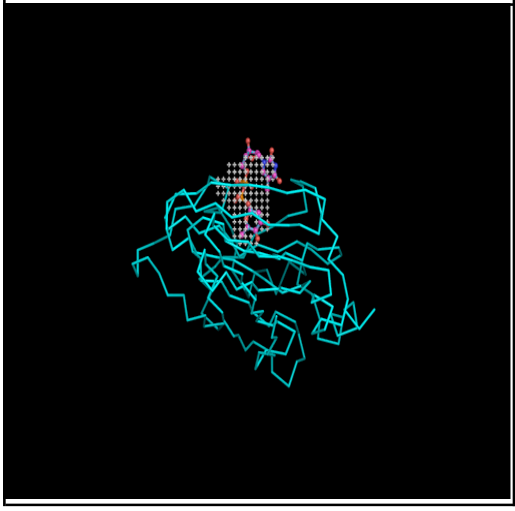	Rv3465	2ixc	A	r2	TRH	1	0.424

148	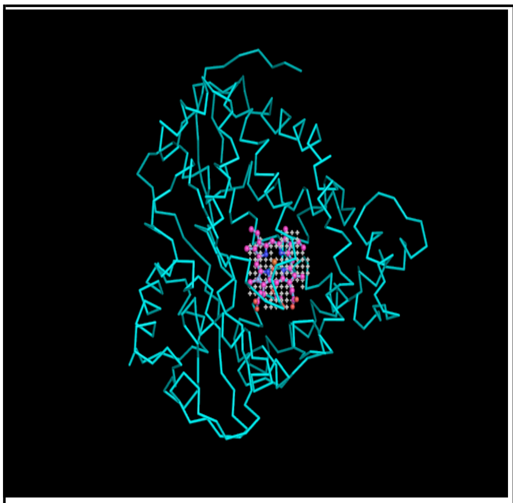	Rv3518c	2xkr	A	r5	HEM	0.68	0.68
149	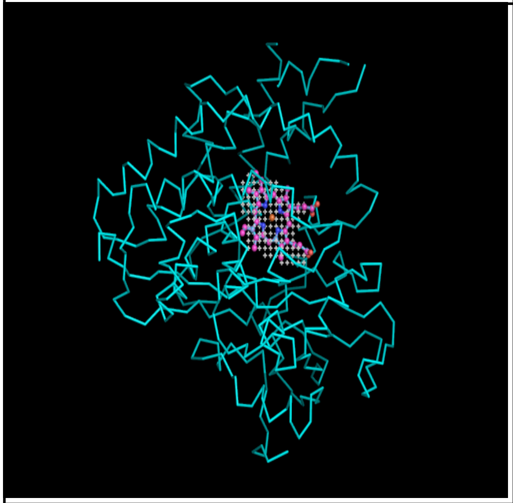	Rv3545c	3ivy	A	r6	HEM	0.863	0.758
150	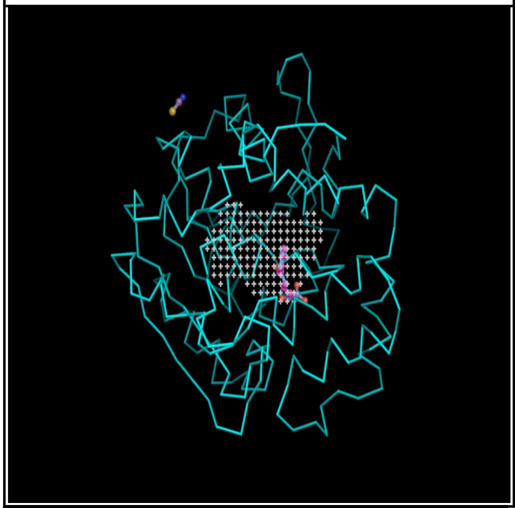	Rv3569c	2wug	A	r2	HPK	0.897	0.092

151	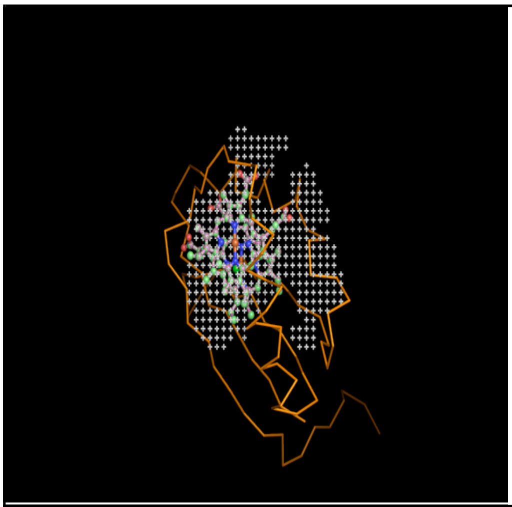	Rv3592	3hx9	A	r2	HEM	0.927	0.286
152	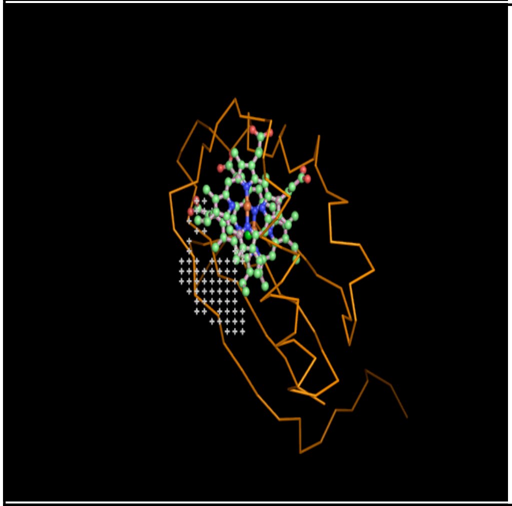	Rv3592	3hx9	A	r4	HEM	0.686	0.223
153	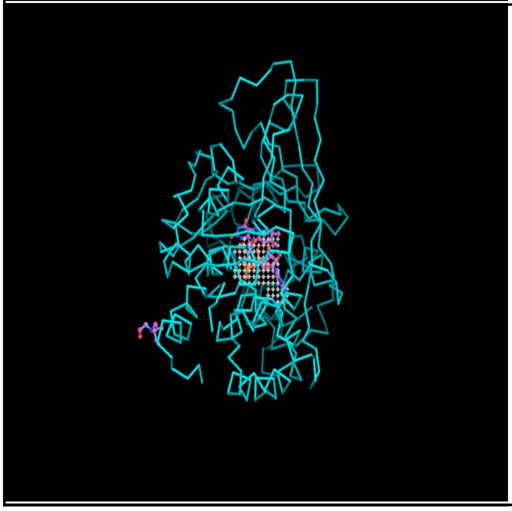	Rv3809c	1v0j	A	r1	FAD	0.855	0.682

154	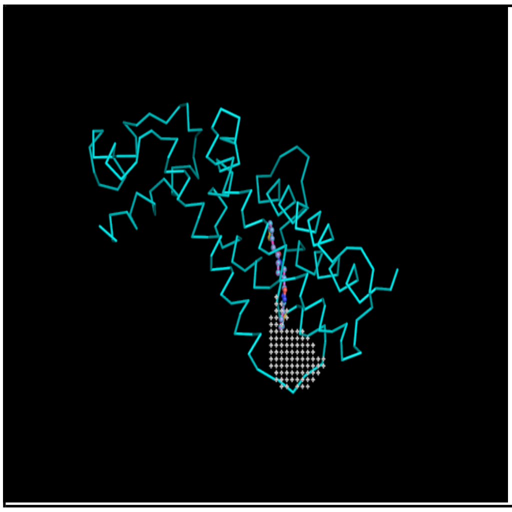	Rv3855	3g1m	A	r2	RF3	0.398	0.326
